# Insights into the demographic history of Asia from common ancestry and admixture in the genomic landscape of present-day Austroasiatic speakers

**DOI:** 10.1186/s12915-021-00981-x

**Published:** 2021-03-29

**Authors:** Debashree Tagore, Farhang Aghakhanian, Rakesh Naidu, Maude E. Phipps, Analabha Basu

**Affiliations:** 1 National Institute of Biomedical Genomics, Kalyani, 741251 India; 2 Oklahoma Medical Research Foundation, Genes and Human Disease Program, 825 NE 13th Street, Oklahoma City, OK 73104 USA; 3 Genomics Facility, School of Science, Monash University Malaysia, Jalan Lagoon Selatan, 47500 Bandar Sunway, Selangor Malaysia; 4 Jeffrey Cheah School of Medicine and Health Sciences, Monash University Malaysia, Jalan Lagoon Selatan, 47500 Bandar Sunway, Selangor Darul Ehsan Malaysia

**Keywords:** Austroasiatic, Linguistic group, Genetic ancestry, Admixture, Migration, Ancient DNA

## Abstract

**Background:**

The demographic history of South and Southeast Asia (S&SEA) is complex and contentious, with multiple waves of human migration. Some of the earliest footfalls were of the ancestors of modern Austroasiatic (AA) language speakers. Understanding the history of the AA language family, comprising of over 150 languages and their speakers distributed across broad geographical region in isolated small populations of various sizes, can help shed light on the peopling of S&SEA. Here we investigated the genetic relatedness of two AA groups, their relationship with other ethno-linguistically distinct populations, and the relationship of these groups with ancient genomes of individuals living in S&SEA at different time periods, to infer about the demographic history of this region.

**Results:**

We analyzed 1451 extant genomes, 189 AAs from India and Malaysia, and 43 ancient genomes from S&SEA. Population structure analysis reveals neither language nor geography appropriately correlates with genetic diversity. The inconsistency between “language and genetics” or “geography and genetics” can largely be attributed to ancient admixture with East Asian populations. We estimated a pre-Neolithic origin of AA language speakers, with shared ancestry between Indian and Malaysian populations until about 470 generations ago, contesting the existing model of Neolithic expansion of the AA culture. We observed a spatio-temporal transition in the genetic ancestry of SEA with genetic contribution from East Asia significantly increasing in the post-Neolithic period.

**Conclusion:**

Our study shows that contrary to assumptions in many previous studies and despite having linguistic commonality, Indian AAs have a distinct genomic structure compared to Malaysian AAs. This linguistic-genetic discordance is reflective of the complex history of population migration and admixture shaping the genomic landscape of S&SEA. We postulate that pre-Neolithic ancestors of today’s AAs were widespread in S&SEA, and the fragmentation and dissipation of the population have largely been a resultant of multiple migrations of East Asian farmers during the Neolithic period. It also highlights the resilience of AAs in continuing to speak their language in spite of checkered population distribution and possible dominance from other linguistic groups.

**Supplementary Information:**

The online version contains supplementary material available at 10.1186/s12915-021-00981-x.

## Background

The peopling of South and Southeast Asia (S&SEA) is complex and contentious. Occupying about 5% of the total landmass, S&SEA is home to over 26% of the world population [1] of diverse ethnolinguistic groups. This region has witnessed multiple waves of anatomically modern human (AMH) migration [2, 3]; the earliest footfalls, as estimated from uniparental genetic data, are as ancient as the first wave of AMH migration out of Africa, ~ 60,000 years ago (60 KYA) [4–6]. Some of the earliest footfalls here were of the ancestors to the present-day Austroasiatic (AA) language speakers [7, 8]. Although their contemporary population sizes are negligible compared to other population groups of the region, independent genetic studies reveal that the ancestors of extant AAs are the earliest inhabitants of both S&SEA [7–11].

It has been estimated that the Austroasiatic language family consists of 150 languages with approximately 65 million speakers [12]. AAs are geographically widespread and their language is spoken throughout mainland Southeast Asia (MSEA), central, northeastern and eastern parts of the Indian peninsula as well as the Nicobar Islands [13]. However, throughout this entire geographical span, AAs have a disjunctive distribution separated by regions where languages from language families such as Indo-European, Hmong-Mien, Tai-Kadai, Austronesian, Sino-Tibetan, and Dravidian are spoken. AA language family comprises of three major subfamilies: Munda, Mon-Khmer, and Khasi-Khmuic [14]. The Munda languages are spoken in central and eastern India while Mon-Khmer and Khasi-Khmuic speakers are scattered from northeast India to the Mekong river basin in MSEA, Malaysian peninsula, and islands of Nicobar. There is also substantial phenotypic diversity among AA subgroups. Such differences are observed both among Indian Austroasiatic speakers [15, 16] as well as Austroasiatic speakers of SEA [17, 18]. The geographical spread of the AAs is enormous: apparently considered a hallmark of successful population expansion, yet locally they mostly live in small isolated populations. Barring a few exceptions, AAs irrespective of their habitat, are exclusively tribal populations [11], collectively embodying an enormous diversity of culture [19–21]. Such scattered geographic distribution has led to two rival hypotheses regarding their origins and migratory routes. The first hypothesis postulates an Indian origin of AA and later eastward dispersal into MSEA [7, 9, 22, 23], while the second hypothesis suggests that AA may have originated in MSEA or southern China and later migrated to India [24, 25]. The spread of AA language and its people has also been related with Neolithic expansion accompanied by the spread of rice cultivation [14, 26]. Yet, to date, almost all AAs across S&SEA, such as Malaysian Negritos, Mlabri of Thailand, Nicobarese from the Nicobar Islands, and Munda speakers from India remain predominantly hunter-gatherers or partial and primitive agriculturists who hardly depend on agriculture for sustenance [10, 19, 20, 27, 28].

Most previous studies on AAs of S&SEA have focused on studying uniparental markers [8, 9, 23, 24, 29] while a few on genome-wide variations [30]. In this paper, we provide insights into the tribal AA groups from two distinct geographical regions: Central India (Munda speakers) and Peninsular Malaysia (Mon-Khmer speakers); we call them AAs of India (AAI) and AAs of Malaysia (AAM) respectively. Using genome-wide genotype data, we target our analysis to answer the following three unresolved inter-related questions: (1) Why in spite of being regarded as the autochthones of S&SEA are their census presence so limited? (2) Why in spite of a wide geographical presence, which is considered a hallmark of successful expansion, are the AA populations fragmented, isolated and small? (3) If the widespread distribution of the AA language is a result of technological advantage and if it has happened post-agriculture, why is it that almost all extant AA population groups are tribal hunter-gatherers, or primitive agriculturists?

To address these questions, we investigated the genetic relatedness of the AA groups (AAI and AAM) along with their relationship with other ethno-linguistically distinct populations in South, Southeast and East Asia. Subsequently, we studied the relationship of these groups with genomes of 43 archaic individuals living in S&SEA at different time period (200 to 8000YA) to infer about the demographic history of this region.

## Results

### Population structure and admixture in S&SEA

The AAs are scattered discontinuous population isolates, surrounded by many other populations. In order to study the genetic relationship of the AAs, with respect to their geographical neighbors, we looked into the population genetic structure that exists among them. We pooled common SNPs genotyped in three different platforms (details in “Materials and Methods”) and ended up with nearly 0.3 million SNPs. We performed a principal components analysis (PCA), as implemented in EIGENSOFT [31], on 939 individuals’ genomes, 92 of which belonged to AAI, 97 to AAM, and 750 individuals from the neighboring Indian, Malaysian, and other Eurasian populations (details of the datasets used are in the “Materials and Methods” section and Supplementary Table 1a-c in Additional file 2).

**Fig. 1.**
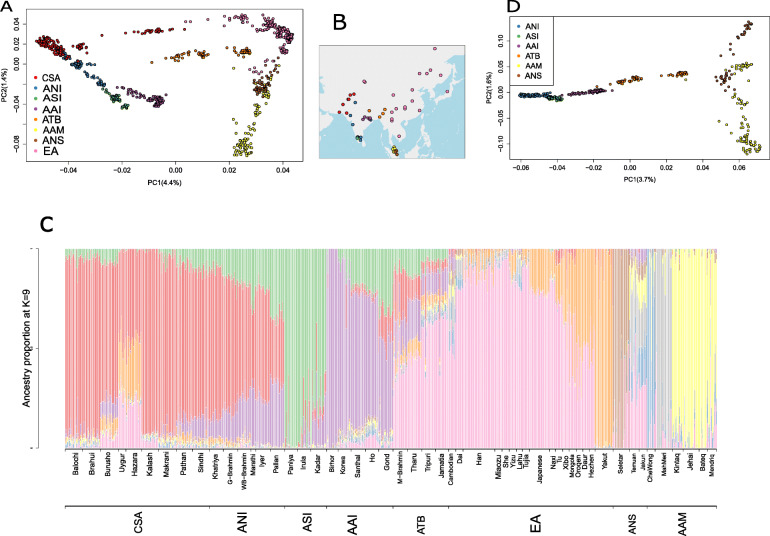
Population structure within Asia. **a** Principal component analysis (PCA) on subgroups belonging to four Indian mainland populations (ANI, ASI, ATB, and AAI), two Malaysian populations (AAM, ANS), and two populations from HGDP dataset (CSA and EA). Each dot represents an individual and each color represents a population. **b** The geographic location of the subgroups of the different populations used in the PCA. Each dot is a subgroup and each color of dots corresponds to the population to which they belong. The population color codes are the same as used in PCA. **c** ADMIXTURE analysis on the same set of populations as used in **a**. The bottom most *x*-axis label represents the populations as in **a**. The other *x*-axis label represents the different subgroups belonging to these populations. **d** Principal component analysis on a subset of those used in **a**, comprising only the four Indian mainland populations (ANI, ASI, ATB, and AAI) and two Malaysian populations (AAM, ANS). The color codes remain the same as used in **a**

In the PC1-PC2 space, individuals belonging to the major population groups (group details and abbreviations in the **“**Materials and Methods,” Supplementary Table 1a-c and Abbreviation section) form unique clusters (Fig. 1a) and their distribution shows approximate correspondence to their current geographic location (Fig. 1b). The AAI adjacent to the “ANI-ASI” cline [32] appears close to ASI, recapitulating previous findings [8, 32]. On the other extreme of the PC1-axis are individuals belonging to AAM, Austronesians (ANS), some Tibeto-Burman speaking (ATB) subgroups, and most East Asian (EA) groups. The AAM formed a distinct cluster along PC2 axis and this cluster was close to ANS and some ATB subgroups.

We estimated the genomic ancestries and admixture proportions at an individual level using model-based clustering analysis as implemented in ADMIXTURE [33] (Fig. 1c, Supplementary Figure 1a-c in Additional file 1). We ran ADMIXTURE multiple times and found the cross-validation error was minimum at *K* = 9 (both for unfiltered as well as the LD pruned at *r*^2^ = 0.1 and *r*^2^ = 0.5 subset of SNPs) (details in “Materials and Methods,” Supplementary Figure 1a in Additional file 1)**.** At *K* = 9 the root mean square error of the ancestry proportion estimates, using bootstrapping implemented in ADMIXTURE, was 0.256. ADMIXTURE recapitulates the previous observation of four ancestral components in mainland Indian populations [8] and shows very little shared ancestry with the Malaysian population. While sequentially increasing “K” to reach the optimum number of ancestral components, the Indian and Malaysian populations clustered distinctly at *K* = 3 while the ANI and ASI separated at *K* = 4 (this pattern was consistent over several independent runs of the program). We also observe distinct ancestries being identified for some subgroups: like Seletar and MahMeri [Seletar as a separate subgroup from other ANS when we increase *K* from 4 to 5 (brown in color, Supplementary Figure 1b in Additional file 1) and MahMeri separate from the remaining AAM when we increase *K* from 6 to 7 (gray)]. Identifying such small isolated populations as separate ancestries (brown for Seletar and gray for MahMeri in the figure) is probably because of the large impact of random genetic drift upon their small effective population sizes, resulting in a quick change to their allele frequency distribution. The impact of drift is also evident in the elevated identical by state (IBS) segments between individuals of these subgroups and a higher proportion of Runs of homozygosity (ROH) within the genome of each individual of these subgroups. (Supplementary Figure 2a-b in Additional file 1)**.** In our ADMIXTURE-based cluster analysis (Fig. 1c, Supplementary Figure 1b), the AAI and the ASI-related populations separate late, at *K* = 6 (purple and green respectively), indicating similarity among the groups. The individuals classified as ANI are genetically similar to individuals of populations from Central South Asia (CSA; colored red), while the individuals classified as ATB are similar to population groups from EA (pink). We also observe two distinct ancestries among the EA based on their geographic location: ancestry predominant among the “Southern” East Asian (pink) and increasing proportion of “Northern” East Asian ancestry (orange), as we move northwards.

For a closer perspective, we conducted a PCA (Fig. 1d) on all mainland Indian populations (i.e., all populations except those belonging to “Island ancestry” as in Supplementary Table 1a) and the Malaysian populations. The first PC captures the systematic variation, separating the “ANI-like” populations on one extreme and the Malaysian populations on the other. The separation of the mainland Indian population into four distinct clusters, namely ANI, ASI, AAI, and ATB, is in agreement with our previous report of four mainland Indian ancestries [8]. The second PC separates the Malaysian population into two major clusters, namely AAM and Austronesian (ANS), who have distinct linguistic identities. Again consistent with our previous findings, we find the populations within the AAM and the ANS form separate clusters [34]. Although previous studies have postulated that language is the best proxy for identifying genetic clusters in the subcontinent [35], our analysis shows that AAI and AAM, who belong to the same linguistic group (Austroasiatic), do not cluster together, nor are they the closest clusters in either Fig. 1a or Fig. 1d. Instead, AAI is apparently closer to the geographically proximal ASI-related populations. Conversely, Tibeto-Burman speakers of India (ATB), especially individuals belonging to the Jamatia and Tripuri populations, who among the ATB are least admixed with other Indian population groups, cluster close to the geographically distant Malaysian populations.

We investigated whether the clustering pattern observed in our PCA analyses is robust if we take linkage disequilibrium (LD) and haplotype structure into consideration. Using fineSTRUCTURE [36], the haplotype-based clustering method, we find two major superclusters/clades (Supplementary Figure 3 in Additional file 1). One contains all individuals from the Malaysian populations (brown colored nodes) and additionally a sub-clade of the mainland Indian population ATB (orange-colored nodes), whereas the other contains mainland Indians namely individuals belonging to AAI, ASI-related, ANI-related (denoted by purple, green and blue colored labels respectively) reiterating our PCA- and ADMIXTURE-derived finding.

To investigate substructure at the population level, on the same set of populations (as in Fig. 1d and Supplementary Figure 3), we surveyed the allele frequencies and calculated pairwise *F*_st_ (Weir and Cockerham [37]) between them using PLINKv1.9 [38] (Supplementary Figure 4 in Additional file 1). Among the mainland Indians, F_st_ between the subgroups of the major ancestral groups (as classified under Column 1 of Supplementary Table 1a) was low (ranging from 0.0005 between Marathi and Pallan to 0.077 between Jamatia and Paniya) compared to *F*_st_ between AAM and ANS subgroups (ranging from 0.025 between Jakun and Temuan to 0.131 between Bateq and Seletar). Populations like Seletar, MahMeri, and CheWong, exhibiting high overall *F*_st_ with all populations, strengthen the possibility of these populations being strongly drift affected (also observed in Supplementary Figure 2). The average *F*_st_ between populations within mainland India is 0.031 and within mainland Malaysia is 0.063. whereas the average *F*_st_ between populations, one chosen from mainland India and another chosen from Malaysia is 0.082. Overall, populations that are geographically distant show higher *F*_st_ as expected. The TB speaking populations which appeared in the same cluster with AAM and in a different cluster to the geographically closer AAI in our fineSTRUCTURE analysis, however, showed lower *F*_st_ with AAI (mean = 0.042) compared to AAM (mean = 0.065).

We further quantified the concordance between genetic relatedness and geographic location of these populations. The scatterplot of pairwise genetic and geographic distance between the populations reveal an overall positive correlation (Supplementary Figure 5a) and a formal mantel test [39] (Mantel statistic *r*: 0.572, *p* = 1e−04) shows an overall concordance of genetics and geography. As we are primarily interested in exploring the diversity and evolutionary history of the AA speakers, we repeated the same with the AA populations (AAI + AAM). When we look into the correlation by choosing each population from AAM group compared pairwise with each population from the AAI group, the correlation turns out to be negative (Correlation coefficient = − 0.446); in agreement with our previous claim that the genetics-geography concordance is not uniform. We repeated the same analysis by masking non-AAI ancestry within the AAI subgroups (as observed in Fig. 1c) and non-AAM ancestry within the AAM subgroup (details in “Materials and Methods”), and we found the correlation increasing (correlation coefficient = 0.125) (Supplementary Figure 5b-c in Additional file 1). This increase in the genetics-geography correlation after removing the genetic components which these populations have acquired through admixture with other populations indicates towards a deep underlying common ancestry between the two groups.

### Tibeto-Burman speakers are genetically closer to the AAM compared to the physically proximal AAI

**Fig. 2.**
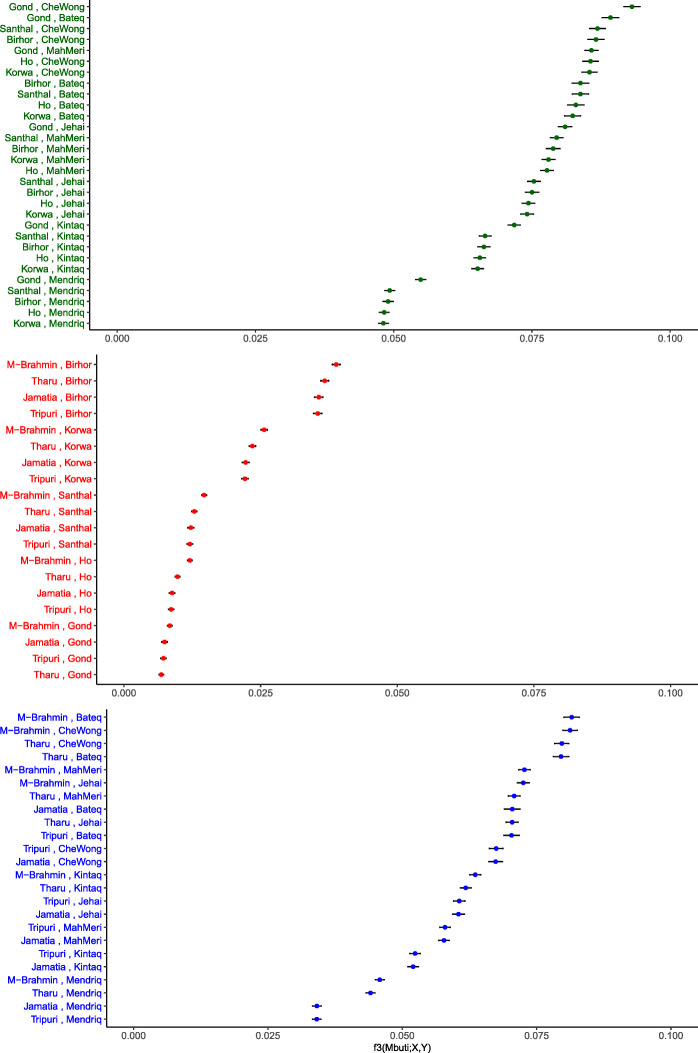
Distribution of Outgroup *f3* (Mbuti; X,Y) values compared between subgroups of different populations X and Y. The *x*-axis represents the *f3* value and *y*-axis represents the pair of populations belonging to X and Y, where X and Y interchangeably belong to AAI, AAM, and TB subgroups. The *y*-axis labels and data points are colored dark green, red, and blue for (X,Y) being subgroups of (AAI,AAM), (TB,AAI), and (TB,AAM) respectively

We recollect that in our PCA (Fig. 1d), the TB speaking individuals, who live physically close to AAI and other mainland Indians, were clustered along PC1 closer to the Malaysian populations (AAM and ANS). The allele frequency-based methods, including *F*_st_ being extremely sensitive to population drift, prompted the computing of outgroup *f3* test [40]. In our analysis, *f3* (Mbuti Pygmy; Y, X) we used the African Mbuti Pygmy from the HGDP dataset as the outgroup (the results are invariant to the choice of other African populations as outgroup and are not shown). We measured the *f3* values between AAM, AAI, and TB (details in “Materials and Methods”) and looked at their distribution (Fig. 2, and Supplementary Table 2a-c in Additional file 2). The mean *f3* values are highest (mean = 0.074) between AAM and AAI groups of populations, indicative of an exclusive and recent shared genetic history. The mean *f3* values were higher (mean = 0.063) for TB-AAM than between TB-AAI (mean = 0.018).

We also generated a phylogenetic tree with AAI, TB, and Malaysian populations using the program TreeMix v1.12 [41] (Supplementary Figure 6 in Additional file 1). To maintain consistency with our *f3* calculations, we used the Mbuti Pygmy population as an outgroup. The AAM populations, especially the MahMeri, and the Seletar among the ANS, with long branch lengths from the leaf to the preceding node, show strong evidence of being affected by random genetic drift. Comparatively, the effect of drift is much less apparent in the AAI populations. Consistent with our other analyses, we observe that the TB populations share clade with the Malaysian populations, while AAI formed a distinct cluster. To indicate possible admixture between populations, TreeMix adds migration edge to the phylogeny. Assuming 3 admixture events in the TreeMix model (which explained 98.95% of the variance), we found a migration edge joining Tharu and the ancestral population of Jamatia and Tripuri (indicating admixture within the TB subgroups). We also observed another migration edge originating in the branch common to Temuan and MahMeri and terminating at the node common to Jamatia and Tripuri. The third edge on this tree is between the node common to Birhor and Korwa (AAI) and the node common to Jehai, Bateq, Kintaq, and Mendriq (AAM). The position of this edge suggests that this gene flow happened prior to the complete separation of all the AAI and AAM lineages, indicative of deep common ancestry.

### Deep common ancestry between AAI, AAM, and TB

Our outgroup *f3* analysis provided evidence of shared genetic history between AAM, AAI, and TB. Using Beagle FastIBD (v 4.1) [42], we estimated genomic segments which are identity by descent (IBD) between each pair of individuals belonging to different subgroups of AAI, AAM, and TB**.** (details in “Materials and Methods”). In Table 1, for all pairwise comparisons, we report the minimum and maximum number of IBD bases along with minimum and maximum number of contiguous IBD segments**.** The AAI and AAM, who apparently are genetically distinct (*F*_st_ = 0.05), share as low as 253 Mb and as high as 1745 Mb (Table 1) of IBD while a single IBD segment is as long as 12.2 Mb (between Ho and Jehai, Supplementary Table 3a in Additional file 2). Additionally, we also see a large number of IBD segments shared between AAI and AAM subgroups. For instance, the two of the most homogenous populations (as inferred from Supplementary Figure 2b) namely MahMeri (AAM) and Birhor (AAI) share a total number of 1032 segments corresponding to 1203 Mb. (Supplementary Table 3a in Additional file 2, Supplementary Figure 7a-e in Additional file 1)**.** Individuals from AAI and TB subgroups share as much as 1024 Mb (between Tripuri and Santhal, Supplementary Table 3b in Additional file 2, Supplementary Figure 8a-e in Additional file 1) and as many as 921 segments (Manipuri-Brahmin and Santhal). On the other hand, individuals from AAM and TB share as much as 2609 Mb and as many as 2078 segments (between MahMeri and Jamatia; Supplementary Table 3c in Additional file 2, Supplementary Figure 9a-d in Additional file 1). This suggests a deep common ancestry between the ancestors of present-day AAI, AAM, and TB.

**Table 1:** Summarized IBD sharing between populations: Each individual of a subgroup within Population 1 is compared with each individual of a subgroup of Population 2 for total number of IBD bases and the total number of discrete IBD segments. For all such pairwise comparison, the minimum and maximum number of bases and the minimum and maximum number of discrete segments is reported

Population 1	Population 2	Minimum bases shared between subgroups (in Mb)	Maximum bases shared between subgroups (in Mb)	Minimum no of IBD segments shared between subgroups	Maximum no of IBD segments shared between subgroups
AAI	AAM	253	1745	214	1385
AAI	TB	348	1024	363	921
TB	AAM	134	2609	107	2078

### Admixture between neighboring populations

Populations which currently live in geographical proximity, show a lot of sharing of genetic components as is evident in our ADMIXTURE analysis (Fig. 1c). The presence of nearly 16.7% ASI-related ancestry (green component in Fig. 1c) in AAI and 7.5% of AAI-related ancestry (purple component in Fig. 1c) in ASI is indicative of significant yet asymmetric admixture between them (Supplementary Table 4a in Additional file 2). The inferred ancestry of the TB populations is similar to the East Asians, particularly those residing near SEA (such as Cambodian and Dai). We designate this commonality as the “Southern EA ancestry” component (pink color in Fig. 1c) and it accounts for ~ 68% (Supplementary Table 4a) of the genomes of all EA populations taken together and 53% of the genomes of TB speakers of India with variable proportions within the subgroups (Supplementary Table 4b in Additional file 2). This Southern EA ancestry is a strong component (5.4%) of the AAM populations, particularly CheWong and MahMeri, but is absent in the AAI.

**Fig. 3.**
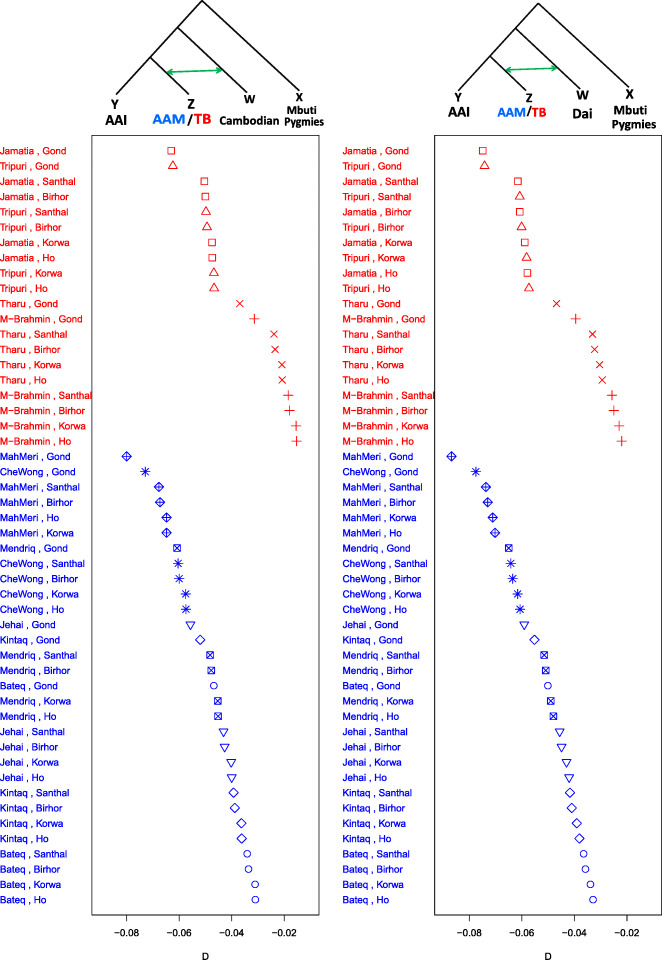
Distribution of D statistics values of the form D(((Z,Y)W) Mbuti Pygmies). The *x*-axis represents the D values. The *y*-axis represents the pair of populations belonging to Z and Y. For the left panel, W is Cambodian while for the right panel W is Dai. In each panel, Y is an AAI subgroup while Z is either TB subgroup (data points and labels colored red) or AAM subgroup (data points and labels colored blue). For each panel, the tree topology is provided at the top and green arrow connecting the two branches represent gene flow between those populations. The corresponding *Z* scores can be found in Supplementary Table 5 a,b in Additional file 2

Further evidence of gene flow between “Southern EA populations” (EA having substantial Southern EA ancestry), TB speakers of India, and AAM comes from D statistics [43], which measures the extent to which derived alleles are shared across populations. We computed *D(((Z, Y), W), X)*, for all subpopulations of AAI (Y), AAM, and ATB (Z), a southern EA subgroup (W=Cambodian and Dai) and Mbuti Pygmy (X, as an outlier), i.e., *D(((AAI, AAM/ATB), Southern EA), Mbuti Pygmies)* (Fig. 3 and Supplementary Table 5a, b in Additional file 2). D statistics reveal no evidence of admixture between Southern EA and AAI populations but shows significant geneflow (|*Z*-score| > 3) between Southern EA and both TB (average D statistic value = − 0.4) and AAM (average D statistic value = − 0.5). Among the TBs the highest evidence of geneflow from Southern EA was obtained for Jamatia and Tripuri (mean for each = − 0.06) and among AAM, for MahMeri (mean = − 0.07). Unlike the AAM who had similar D values when compared to both Cambodian and Dai (mean = − 0.05), the absolute D values were higher (*p* value ~ = 0.04) for TBs with Dai (mean = − 0.05) than with Cambodian (mean = − 0.04). This observation is in agreement to the fact that the Dai subgroup resides geographically more close to the TBs than the Cambodians do.

To further investigate East Asian admixture into TB and AAM subpopulations, we performed local ancestry estimation, using RFMix [44] (details in “Materials and Methods”). We identified regions within genomes of both AAM and TB individuals representing EA ancestry. We estimated the length of such regions and looked into their distributions in each of the subpopulations. We found long tracts of EA ancestry in both TB and AAM. However, the average length of EA ancestry genomic segments was larger in TB than in AAM (*p* < 2.2e−16) (Supplementary Fig. 10 in Additional file 1).

**Fig. 4.**
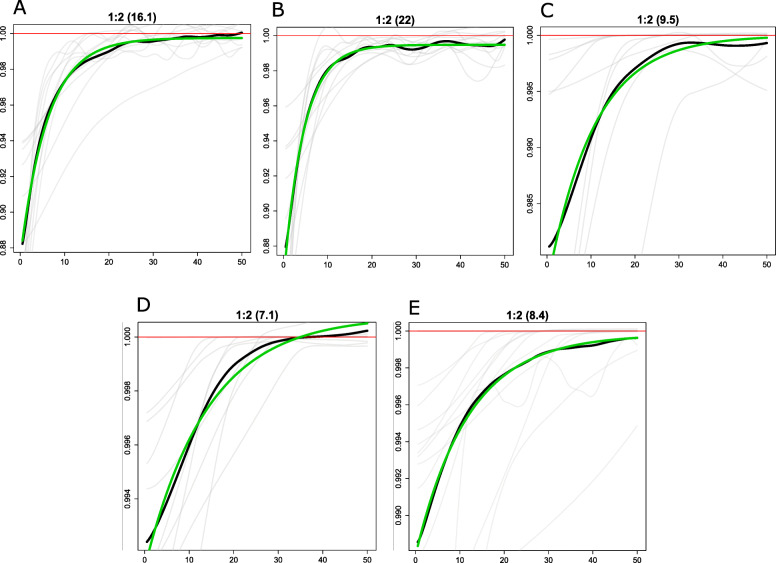
Admixture dating using coancestry curves. The *y*-axis is the relative probability of jointly copying two chunks from the donor populations and *x*-axis is the genetic distance in centimorgans. The green line represents the fitted curve, the black line represents the across targets observed ratios, and the gray lines represent the per target ratio. At the top of each panel is the index of the pair of ancestries being examined as a:b (where a and b represent the indices of the ancestries). The adjacent number inside parenthesis represents the number of generations since admixture. For the different panels, a:b represent **a** MahMeri:Yakut and Mendriq as admixed group, **b** MahMeri:Yakut and CheWong as admixed group, **c** MahMeri:Yakut and Cambodian as admixed group, **d** MahMeri:Yakut and Tu as admixed group, and **e** MahMeri:Yakut and Jamatia as admixed group

We dated these local admixture events using a method implemented in MOSAIC [45] that infers admixture time by fitting exponential decay coancestry curve (details in Materials and Methods). We chose two homogeneous source populations, Yakut for EA ancestry and MahMeri for AAM. We found that the last evidence of admixture between EA and AAM took place as early as 22 generations ago to as recent as 7.2 generations ago (Fig. 4 and Supplementary Table 6 in Additional file 2).

We repeated the analysis by choosing different East Asian populations with varying geographical distance from AAM as EA ancestry source and MahMeri and Jehai as AAM ancestry source (Supplementary Table 6). For the same recipient population (Cambodian), admixture dates ranged from 9.5 generations ago (MahMeri and Yakut) to 6.1 generations ago (Jehai and Daur).

This suggests that there has been multiple admixture events of EA populations with different AAM subgroups, and although each has a unique history, the last admixture event has been very recent.

We had also observed ASI like ancestry in AAI (Fig. 1c and Supplementary Table 4), and the last admixture event between them were dated to be in the range of 17.5–11.1 generations ago. Small proportion of EA-like ancestry that was observed in AAI was likely due to admixture between TB and AAI, though the genetic component of TB is comparable to many of the East Asian populations. However, we recollect that we did not find admixture between EA and AAI in D statistics analyses, (Fig. 3), rather we found admixture of TB and AAM with EA. We estimated the time for the last admixture event between these population groups to be as recent as 11.3 generations ago (details in “Materials and Methods,” Supplementary Table 6).

### Population separation and fate of the population subgroups post-separation

**Fig. 5.**
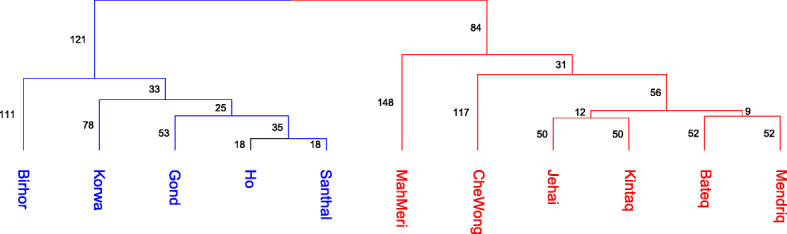
Separation of AAM and AAI using NeON. Red color represents AAM subgroups and blue represent AAI subgroup. The numbers represent branch length

We estimated the population divergence times, for each subgroup pairs belonging to AAI and AAM using NeON [46]. The estimated divergence time between populations belonging to AAI and populations belonging to AAM superclades was greater than that among the populations belonging to either AAI or AAM (Table 2). Using the matrix of the estimated divergence times, we constructed a UPGMA tree (Fig. 5) on population divergence time to infer about the chronology of the separation events and the phylogenetic relationship between the populations. AAI and AAM formed separate clusters and that the separation happened nearly 470 generations ago. Within the AAI branch, the first to separate were the Birhor followed by Korwa, Gond, Santhal, and Ho. In the AAM branch, the MahMeri initially separated from the rest of the AAM, followed by CheWong. A further split gave rise to Bateq and Mendriq on one hand and Jehai and Kintaq on the other. Subsequent splits led to the separation of Bateq from Mendriq and finally the separation of Jehai and Kintaq. We recollect here that both MahMeri and Birhor individuals have high IBD segments with populations from the different super clade, i.e., Birhor with AAM populations (MahMeri, CheWong, Bateq, Mendriq, Jehai, Kintaq) and MahMeri with AAI populations (Ho, Korwa, Santhal, Gond).

**Table 2:** Population divergence time: a matrix of the time of divergence between populations estimated as number of generations

	Bateq	Jehai	Mendriq	Kintaq	CheWong	MahMeri	Birhor	Gond	Ho	Korwa	Santhal
**Bateq**	0	140	104	136	252	318	369	597	506	406	491
**Jehai**	140	0	109	99	241	300	382	628	524	422	509
**Mendriq**	104	109	0	104	213	285	350	470	385	353	393
**Kintaq**	136	99	104	0	231	298	360	553	465	386	456
**CheWong**	252	241	213	231	0	274	371	594	493	404	484
**MahMeri**	318	300	285	298	274	0	391	656	534	435	525
**Birhor**	369	382	350	360	371	391	0	288	209	195	194
**Gond**	597	628	470	553	594	656	288	0	97	202	115
**Ho**	506	524	385	465	493	534	209	97	0	139	36
**Korwa**	406	422	353	386	404	435	195	202	139	0	127
**Santhal**	491	509	393	456	484	525	194	115	36	127	0

If a population experiences fission, it is expected that the resulting populations will show a decrease in the effective population size (*N*_e_) [47]. Using McEvoy et al’s method [48] as implemented in NeON, we calculated the *N*_e_ (Supplementary Figure 11 in Additional file 1). We observed a continuous decline in *N*_e_ among all AAM (Supplementary Fig. 11a) and in Birhor and Korwa among the AAI (who were among the first to separate from within the AAI branch). However, post AAI-AAM separation Gond, Ho and Santhal among the AAI showed an increase in *N*_e_ followed by a decline around 350 generations ago (Supplementary Figure 11b). We also estimated effective population sizes for ANS, TB and EA subgroups. In the three ANS subgroups, we observed a continuous decline in *N*_e_ (Supplementary Figure 11c). Among the TBs, all subgroups show an initial steep increase in *N*_e_ (except Tharu) followed by a gradual decrease from 400 generations onwards (Supplementary Figure 11d). Among the East Asians, with the exception of Japanese and Han, all subgroups show a decrease in *N*_e_, most of who are minority ethnic groups (Supplementary Figure 11e).

### Temporal variation in ancestry of SEA

**Fig. 6.**
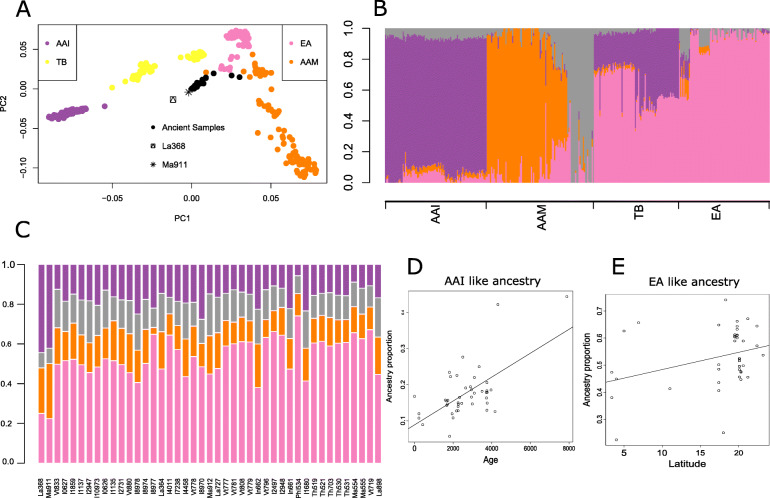
Ancient genomes. **a** PCA on the ancient genomes along with all subgroups of AAI, AAM, and TB and a few East Asian subgroups (Han, Dai, Naxi, Yizu, and Cambodians). The ancient genomes are colored in black with the two most ancient samples labeled differently in black. **b** ADMIXTURE analysis on the same set of population as in **a**. **c** Projection ADMIXTURE analysis on the ancient samples using the components inferred in **b** and colored accordingly. **d** Correlation graph of age of ancient samples vs proportion of AAI ancestry. **e** Correlation graph of latitudinal positions of ancient samples vs proportion of EA ancestry

In addition to genotypes of extant samples, we analyzed genotypes of 43 ancient DNA samples, excavated from different locations of SEA [49, 50], dating between 0.2 and 8 KYA (details in Supplementary Table 7 in Additional file 2). Lipson et al. [49] genotyped 18 ancient DNA collected from 5 different sites: Man Bac (Vietnam, Neolithic; 4.1–3.6 KYA), Nui Nap (Vietnam, Bronze Age; 2.1–1.9 KYA), Oakaie 1 (Myanmar, Late Neolithic/Bronze Age; 3.2–2.7 KYA), Ban Chiang (Thailand, Late Neolithic through Iron Age; 3.5–2.4 KYA), and Vat Komnou (Cambodia, Iron Age; 1.9–1.7 KYA). McColl et al. [50] did a low-coverage whole-genome sequencing of 26 ancient human genomes (25 of which used in this study were from mainland SEA spanning Malaysia, Thailand, the Philippines, Vietnam, Indonesia, and Laos, ranging from ~ 8 to 0.2 KYA; Age distribution of these samples in Supplementary Figure 12 in Additional file 1). To compare the genomes of extant populations to individuals who lived in the region at different time points, we ran a PCA on genomes from the extant AAI, AAM, TB, and neighboring EA populations of HGDP (Han, Dai, Naxi, Yizu, and Cambodians) (Fig. 6a) along with the genomes from ancient samples. The ancient samples (ANC) formed a separate cluster from the extant populations, with a few individuals very closely positioned to the AAM and EA individuals. We observe a cline along the PC1-PC2 scatter plot. The oldest ancient genomes from Pha Faen, Laos (La368; dated 7.950 to 7.7 KYA) and Gua Cha, Malaysia (Ma911; dated 4.4 to 4.1 KYA), though sampled from completely different geographical locations, were “outliers” among the ANC cluster and are the points closest to the AAI populations in the PCA plot (Fig. 6a).

Using the extant genomes of individuals included in the PCA, we did an ADMIXTURE analysis and found that 4 ancestries best explain the dataset (Fig. 6b and Supplementary Figure 13a-b in Additional file 1**)**. These ancestries roughly correspond to AAI (purple in color) and EA (pink in color) while the AAM populations are split into 2 ancestries, one comprising mainly of the MahMeri like ancestry (gray in color) and the other comprising of Bateq, Mendriq, Jehai, and Kintaq (orange in color). On this we projected the ancient genomes (Fig. 6c) and found that the oldest genomes (La368 and Ma911) had the highest proportion of AAI-like ancestry (purple in color) and the lowest proportion of EA-like ancestry (pink in color). We also found a significant positive correlation between the antiquity of the ANC samples and AAI-like ancestry (*r* = 0.62, *p* = 6.9 × 10^− 6^) (Fig. 6d) and a negative correlation between the antiquity of ANC samples and EA-like ancestry (*r* = − 0.59, *p* = 2.6 × 10^− 5^) (Supplementary Figure 14a in Additional file 1). Thus, the older ANC genomes were more similar to AAI genomes and had lesser EA-like ancestry compared to the newer ones. However, we did not find significant correlation between the antiquity of ANC samples and AAM-like ancestry. McColl et al. [50] suggested that ancient SEA hunter-gatherers (Hòabìnhian) share some ancestry with the Onge, Jehai, Papuan, and Indian populations. We therefore ran the ADMIXTURE analysis including the Jarawa, Onge, and the Papuans as possible founder populations in addition to the previous set of AAI, AAM, TB, and EA. Contrary to their claim, we found no evidence of Onge, Jarawa, and Papuan ancestries in the ANC samples (results of ADMIXTURE run hence not shown). We regressed the AAI ancestry (and the EA-like ancestry) of the ancient genomes jointly on the age of the sample and the latitude where these samples were found (Supplementary Table 7). While latitude was only marginally significant for the AAI-like ancestry, it was extremely significant for EA-like ancestry, showing a decreasing trend of EA-like ancestry as one moves from North to South (Fig. 6e, Supplementary Figure 14 in Additional file 1). This bolsters the hypothesis of the origin of EA-like ancestry in Southern China and a movement due south.

For a deeper understanding of how gene flow from East Asia influenced the genomic composition of MSEA over time, we selected 9 ancient genomes as representatives from five time periods. These were grouped as follows: (a) Group Anc_8K comprises the genome La368 which is nearly 8000 years old (8KYO), (b) Group Anc_4K comprise of ~ 4KYO genomes (I1859 and I1137), (c) Group Anc_3K comprise of ~ 3KYO genomes (I4458 and La364), (d) Group Anc_2K comprise of ~ 2KYO genomes (I1680 and In661), and (e) Group Anc_R comprise of ~ 250 years old genomes (Ma555 and Vt719). D statistics was computed for the five ancient genome groups with AAM subgroups, EA (Cambodian), and Mbuti Pygmies in separate branches (details in “Materials and Methods”). We observed significant negative *Z* scores (Supplementary Figure 15 in Additional file 1 and Supplementary Table 8 in Additional file 2) when Group Anc_8K was used for the analysis indicating no gene flow from East Asia in the oldest genome. However, subsequent analysis with the rest of the groups resulted in significant positive *Z*-scores, indicating East Asian admixture. The D values increased as the age of the ancient genomes decreased which suggests increasing gene flow from East Asia over time.

## Discussion

Geography has been the best proxy to genetic distance in genome-wide studies of variation across the world [51–53]. In studies that look deep into local histories; language, a proxy of the broad culture of a population, often explains a large proportion of the genetic variation [8, 54–58]. In the context of India and South Asia, it has been observed that linguistic differences of populations provide the best predictor of genetic differences [35]. The current study, however, suggests that neither language nor geography is sufficient in independently explaining the deep intricacies of population structure in S&SEA.

The contemporary physical presence of AAs (AAI and AAM), along with central and eastern India through peninsular SEA, overlaps with the pre-Holocene modern human settlement in Sunda and has been hypothesized as the corridor of first modern human migration to Sahul [6, 59]. It has been proposed in independent studies of mtDNA, which infer the AAs to be the earliest inhabitants residing in their respective lands ~ 60,000 YBP, both in India [7–9] and peninsular Malaysia [30]. The genetic connection is also emphasized by the AA specific Y-chromosome haplotype O-M95, present in high frequency among both AAI and AAM [23]. Although multiple studies of uniparental DNA connect the AAI and the AAM, the contentious issue remains in the identification of demographic movements, dating the migration events, and understanding the mosaic of admixture events with adjacent population groups.

From our initial population structure analysis (PCA, ADMIXTURE), the existence of a genetic relationship between AAI and AAM is not clearly apparent. Rather, we find clear and separate clustering of AAI and AAM. Our findings are consistent with the inferences drawn by Chaubey et al. [25], who found that despite their linguistic affinities, AAs in different geographical regions have distinct admixture history. If we consider a model where two population groups are separated for a long period of time, where both groups get subsequently fragmented and each population fragment isolated into small subpopulations, long-term random genetic drift would generate different and unique genomic signatures for the subpopulations. The smallest of the populations and the ones which are the earliest to separate, resulting from the fission, would be the most drastically affected. On the contrary, this very process is likely to increase the allele frequency, mimicking a “founder effect,” of the most “abundant” haplogroup for both the mtDNA and Y-chromosome. Hence, although the PCA and ADMIXTURE analysis on autosomal data identifies the AAI and AAM as separate clusters, the apparent contradiction with the inferences from uniparental data can be reconciled by the model of a deep separation of the population groups, a fission process described above and subsequent local admixtures.

Our analysis of autosomal data indicates that in pre-Neolithic times, the ancestors to today’s Austroasiatic speakers had a widespread distribution, as tentatively claimed by Lipson et al. [49], possibly extending from Central India across SEA before being fragmented and isolated to small pockets as we see them today. This claim is now supported by multiple lines of evidence: (1) early connection between the populations as inferred from TreeMix, (2) large amount of IBD sharing in absence of any evidence of recent admixture, (3) strong genetic affinity of the Indian AAs (who are relatively unadmixed with the East) with the pre-Neolithic Hòabìnhian hunter-gatherers. However, we acknowledge that the absence of ancient genomes from Central and Northeastern parts of India limits the validation of our claim. This study also reveals that other populations, like the TB speakers Jamatia and Tripuri, who live in the intermediate geographical region separating AAI and AAM, also have a deep underlying genetic similarity with both AAI and AAM. However, they have a much higher similarity with EA populations from Southern China.

Our estimate of the separation time of AAI and AAM lineage, approximately 470 generations ago, predates the advent of agriculture in this region, suggesting Austroasiatic ancestry as a pre-Neolithic phenomenon and not associated with the spread of agriculture as has been widely suggested [49, 60–62]. Our results also disprove the claim that the AA ancestry was introduced by migrating East Asian farmers [50], clearly because we observe negligible EA ancestry in the genetic component of AAI. We also see a decline in the effective population size (after around 350 generations ago) of some AAI who had an initial rise in *N*_e_ post-separation. The period of decline coincides with the advent of agriculture in this region and corroborates with findings of Chakraborty et al. [63]. This further strengthens our argument that peopling of this region by AAs was not a result of migrating East Asian farmers. It is also possible that contrary to previous claims of EA farmer migration beginning around 5KYA [64], the southward migration of EA farmers had begun much earlier. This migration led to the fragmentation of the initial populations of AAs ultimately resulting in their current restricted distribution in remote habitats as isolated foraging groups. The role of geography in shaping the genomes of populations is quite apparent when we consider the geographical positioning of the AAI populations. If we consider their contemporary location, they are cordoned off from the hypothetical South and South-Western migration route of the early farmers of South China, whereas the locations of the AAM in MSEA, or the TB speakers in the North Eastern fringe of contemporary India falls on their migration route.

Demographic factors are known to have a huge impact on culture and behavior of populations [65]. Languages are dynamic and admixture between populations can increase similarity in languages or can result in the replacement of one language by another [66]. Phonemic diversity correlates positively with speaker population size as well as antiquity [67]. Like other cultural traits, languages in isolated populations borrow less and hence evolve slowly [68]. This suggests that phonemic diversity changes over longer time scales in isolated populations. Using this phonemic diversity, the time estimates of the origin of AA languages are pre-Holocene [69]. The isolation of the AA speaking populations has largely insulated them from influences of population groups that surrounded them, especially on their language. The relative similarity of Khasi-Khmuic and Mon-Khmer (spoken by AAM) with languages spoken by populations of EA [70] and the distance of the Mundari group of languages (spoken by AAI) from the EA languages mirror the genetic admixture pattern observed between the AAI, AAM, and EA. From our RFMix analysis, we conclude that the TB speakers not only have significantly higher admixture with EAs but also harbor long EA-specific ancestry segments. This indicates that EAs continued to admix with TB long after admixture between EA and AAM had ceased. The language of the TB speakers, whom we have shown to share a deep common ancestry with the ancestors of AAI and AAM but have a substantially different history of admixture with EAs both in extent and in duration, belongs to the family of the languages spoken by people of South China and Tibet. We postulate that the consistent gradual decline in *N*_e_ of AAM and AAI 350 generations ago onwards (~ 7.7 KYA, assuming a 22-year generation time) points towards forced isolation of foraging societies.

We also found clear differences in the genomic relatedness of ancient genomes to that of present-day AAs, before and after agricultural expansion. The oldest ancient genome (La368) in our study, nearly 8000 years old, was more similar to present-day AAI while the genomes from the period around 4 KYA and later bore more resemblance to present-day AAM. The agricultural expansion brought the EA farmers whose genetic ancestry got incorporated into the genomes of the original inhabitants of SEA. La368, however, lived in times when the migration of EA farmers had only just begun and therefore had minimum East Asian ancestry. Our results, on the other hand, show that present-day AAI also has negligible EA ancestry. This negligible EA ancestry (2.77% Southern Eastern ancestry and 0.41% Northern East Asian estimated using ADMIXTURE) can be a residual of the ancient admixture of Southern EA-like ancestry that we find in La368 and other ancient genomes. We also note the conspicuous similarity between AAI and AAM in the absence of Northern EA component in their genomes unlike the ATB. However, the EA component of AAI can also be derived from more recent admixtures with the contemporary TB populations living in close geographical proximity with the contemporary AAI populations. Thus, the similarity between the oldest genome in our study and contemporary AAI can be attributed to the fact that genomes of both were not influenced by East Asians. On the other hand, the genomes of the remaining ancient individuals were most likely influenced by gene flow from the incoming EA farmers to a varying degree, contributing to the similarity observed with genomes of present-day AAM who have a substantial EA ancestry. We also find that with a decline in the age of our ancient samples, there is an increase in EA-like ancestry indicative of a temporal shift in ancestry. Even previous analyses comparing AA-like and EA-like ancestry in ancient genomes [49, 50] revealed more AA-like ancestry in older samples and more EA-like ancestry in newer ones, thereby bolstering our inferences. Thus, there is clear evidence of ancestry shift in SEA pre- and post-Neolithic expansion.

Our findings agree with previous reports [49, 50] that continuous migration from East Asia did not completely replace the indigenous ancestry; instead, the incoming populations extensively admixed with the indigenous ones. Moreover, there has been archeological, linguistic and genetic evidence supporting movement of people from southern China, across South East Asia to Melanesia and Polynesia.

## Conclusion

The current study emphasizes the importance of both geography and language in reconstructing the population structure of India and SEA. Our study suggests that the ancestors to present-day AA speakers were the resident native population extending from Central India to mainland Southeast Asia. They were hunter-gatherers and spoke possibly some proto-AA language that has given rise to the present-day AA language family. The present-day Indian Austroasiatics and Malaysian Austroasiatics shared a common ancestor until about 10.5 KYA. Post-separation they had a disparate genetic history. Around 7 KYA, with the advent of agriculture, there was an ancestry shift in Southeast Asia. The distribution of AA hunter-gatherers started to shrink and their population size kept declining. As farmers from EA began migrating southwards to Mainland Southeast Asia, the population size of AA hunter-gatherers residing in SEA decreased further. Moreover, the East Asians interbred with local AA hunter-gatherers introducing “East Asian ancestry” in SEA. With subsequent migration waves, substantial EA ancestry was added to the native Austroasiatics residing in MSEA, including AAM in our study. The continuous migration also resulted in rapid fragmentation and isolation of the AA hunter-gatherer population. On the other hand, since the East Asians did not enter peninsular India, the “East Asian ancestry” was not introduced in AAs in India, who mostly retained their genetic ancestry while interbreeding locally with populations of ASI ancestry. The incoming wave of East Asians in SEA may not only have influenced the language of present-day TBs but also contributed to the temporal change in the genetic ancestry of Southeast Asia.

We also report a unique phenomenon where, despite notable changes in genetic identity of individuals, the linguistic identity remains intact. The shared genetic ancestry of the AAI and the AAM that predates the arrival of East Asians and their isolation post local admixture with linguistically distinct neighbors, have contributed to their linguistic similarity. However, the language of AAI and AAM may have changed over time following their separation and isolation giving rise to the Mundari and Mon-Khmer branch of the Austroasiatic language respectively. Thus, this study not only sheds light on genetic history but also provides new insights to the linguistic history of India and Southeast Asia.

## Materials and methods

### Dataset

We obtained genotype data on 367 unrelated individuals belonging to Indian populations from the archives of National Institute of Biomedical Genomics [8]. This dataset referred to as Indian dataset henceforth was genotyped on Illumina Omni 1 Quad version 1.0 and was in hg18 assembly. We then converted this to hg19 assembly. Using PLINK [38] version 1.07 (http://pngu.mgh.harvard.edu/purcell/plink/), we merged this dataset with genotype data of 144 unrelated Malaysian individuals. The data was generated on Illumina Human Omni 2.5 array and was already in hg19 assembly [34]. This is referred to as Malaysian dataset henceforth. We also converted the genotypes of 940 individuals from HGDP [53], genotyped on Illumina HumanHap650K Beadchips, to hg19 prior to merging with the rest of the data. All conversions to hg19 were performed using LiftOver tool of UCSC [71]. We included only autosomal SNPs in our study. The Indian dataset comprises of individuals belonging to five different previously reported [8, 32] genetic ancestries namely Ancestral North Indian (ANI), Ancestral South Indian (ASI), Ancestral Tibeto-Burman (ATB), Ancestral Austroasiatics (AAI), and Island ancestry (Jarwa and Onge from the Andaman and Nicobar Islands). The population subgroups belonging to these ancestries are listed in Supplementary Table 1a in Additional file 2. Apart from this genetic classification, the populations belonging to the Indian dataset can be broadly classified into four major language families based on the language spoken by the individuals belonging to these populations [8]. These are (a) Indo-European linguistic group which comprises of Tharu subpopulation of ATB ancestry and all the subpopulations of ANI ancestry except the Iyer and Pallan subpopulations, (b) Dravidian linguistic group which comprises of all the subpopulations of ASI ancestry and the Iyer and Pallan subpopulation of ANI ancestry, (c) Tibeto-Burman linguistic group which comprises of all the subpopulations of ATB ancestry except Tharu subpopulation, and (d) Austroasiatic linguistic group which comprises of all the populations of AAI ancestry. The populations belonging to the Island ancestry speak a different language which is not very well classified. The Malaysian dataset comprises individuals belonging to two linguistic families namely Austroasiatic (AAM) and Austronesian (ANS) (Supplementary Table 1b in Additional file 2). Our total dataset [72] comprised of 1451 individuals (Supplementary Table 1c in Additional file 2). Most of the abbreviations are consistent with the publications where they first appeared (Supplementary Table 1c).

### Quality control

We included only biallelic loci in our analysis. We removed all monomorphic variants and SNPs with alleles A/T and G/C from our analysis. To address the issue of insufficient data, we removed SNPs with missingness of more than 5% in the entire dataset, or SNPs that were missing in more than 25% of individuals in any of the 14 subpopulations listed in Supplementary Table 1c in Additional file 1. We also excluded SNPs which were out of Hardy Weinberg equilibrium (*p* < 10^− 6^) in any of the 14 subpopulations and were out of Hardy Weinberg equilibrium (*p* < 10^− 2^) in 2 or more subpopulations. The final dataset [72] had 324,253 SNPs.

### Population structure analysis

In order to understand the overall population structure and the genetic affinities of the individuals in our dataset, we performed principal component analyses (PCAs) using smartpca program of EIGENSOFT package [31]. We performed an initial PCA on all the mainland Indian and Malaysian populations and two populations from the HGDP dataset, namely, Central South Asia (CSA) and East Asia (EA). Another PCA was done on only the mainland Indian and Malaysian populations.

In order to compare the EIGENSOFT result with other Population Structure visualization methods, we did fineSTRUCTURE(v0.0.2) [36] analysis. For this analysis, we phased the genotype data of Indian mainland populations and Malaysian populations using SHAPEIT (v2.r790) [73]. We then converted this phased data to fineSTRUCTURE format using the program impute2chromopainter.pl bundled with the fineSTRUCTURE package. We then fed this data into the fineSTRUCTURE algorithm. The results were visualized as dendrograms.

### IBD-based demographic inference

To further explore the genetic relatedness, we used Beagle FastIBD (v4.1) [42] to identify segments that are identity by descent (IBD). IBD segments are long haplotype blocks that have been inherited from a single common ancestor without recombination. We searched for segments that were IBD between each individual of subgroup belonging to AAI and each individual of subgroup belonging to AAM and estimated the length of each such segment. This was done for all pairs of AAI-AAM subgroups. In each pairwise comparison, we removed those segments which were outliers. We also fetched the maximum length of IBD segment shared between an individual of AAI and of AAM subgroups. After removing the outliers, for the remaining IBD segments, we computed the “normalized IBD segment” length as follows:

$$ \mathrm{Normalized}\ \mathrm{IBD}\ \mathrm{segment}\ \mathrm{length}=\frac{\mathrm{Length}\ \mathrm{of}\ \mathrm{the}\ \mathrm{IBD}\ \mathrm{segment}}{\mathrm{Maximum}\ \mathrm{length}\ \mathrm{of}\ \mathrm{IBD}\ \mathrm{segment}\ \mathrm{shared}\ \mathrm{between}\ \mathrm{AAI}\ \mathrm{and}\ \mathrm{AAM}}\times 100 $$

Then we looked at the distribution of these “normalized IBD segment lengths.” We also computed the number of IBD segments shared between each pair of subpopulation. The same method was applied for estimating “normalized IBD segments” between AAI and ATB and between AAM and ATB.

The detailed results of the pairwise sharing between individuals from these disparate subgroups is available in Supplementary Table 3 in Additional file 2 and Supplementary Figure 7–9 in Additional file 1.

### TreeMix

In order to understand how populations were related to each other through a common ancestor, the impact of drift and to obtain evidence of gene flow, we built ancestry graphs using TreeMix [41] version 1.12. Such graphs were created with AAI and ATB subpopulations and all Malaysian subpopulations using the Mbuti Pygmies from Africa (from HGDP dataset) as outgroup under the assumption of possible gene flow.

### Admixture

To infer the different ancestral components present in admixed populations and the proportions of each such component in an individual’s genome, we performed ADMIXTURE [33] (v1.3.0). Using the Maximum Likelihood Estimation (MLE) and cross-validation approach, ADMIXTURE determines the best fitting model. By increasing the number of *K* possible ancestries in each run of the analysis on a given dataset, ADMIXTURE computes a cross-validation error (CVE) and estimates the proportion of each of the *K* ancestry in the genome of each individual of the dataset. The run with the minimum CVE error is considered to be the optimum number of *K* ancestries that best explains the data. We ran ADMIXTURE with all Mainland Indian and Malaysian populations along with HGDP dataset populations namely EA and CSA. This was done in 3 different ways (a) without LD pruning of SNPs, (b) with LD pruning of SNPs at *r*^2^ = 0.1, and (c) with LD pruning at *r*^2^ = 0.5. For each SNP set (i.e., (a), (b), and (c)), we estimated the Standard error of the CVE estimate by running ADMIXTURE multiple times. Minimum CVE error in each case was observed at *K* = 9 but the lowest CVE was when *r*^2^ = 0.1 and *K* = 9. Plots were generated with results of LD pruned dataset at *r*^2^ = 0.1. Standard error was estimated for the ancestry proportion estimates at *K* = 9 using the moving block bootstrap approach implemented in ADMIXTURE. Standard error of each ancestry proportion estimate was generated by running 1000 replicates with *K* = 9 and *r*^2^ = 0.1.

### F_st_ estimates

Using PLINK [38] version 1.9, the weighted *F*_st_ between each subpopulations of mainland India and Malaysia was estimated. These values were rounded to the third decimal place.

### Runs of homozygosity (ROH) and identity by state (IBS)

Using PLINK, we estimated identity by state between individuals and homozygosity runs in the genome of each individual for 17 subpopulations. These subpopulations included all the five AAI subgroups, six AAM subgroups, and three ANS subgroups. Two subgroups of Andaman Island (Jarwa and Onge) who were known to have highly inbred and another subgroup from ANI (WBR) who were outbred from previous reports [8, 74] were used as a reference.

### Outgroup *f*3 statistics

Outgroup *f3* statistics measures the shared drift between two populations relative to an extremely diverged population outgroup. Using ADMIXTOOLS (v5.1) [31], we calculated outgroup *f3* statistics of the form *f3* (Mbuti Pygmy; X, Y) where Mbuti Pygmy was the outgroup. Keeping X as an ATB subpopulation, Y was AAI or AAM subpopulation. With X being AAI subpopulation, Y was an AAM subpopulation. This allowed all pairwise comparison between subpopulations of ATB and AAM, ATB and AAI, and AAI and AAM.

### Admixture time

Segments resulting from admixture follow an exponential distribution from which number of generations since admixture can be estimated [75]. To date events of admixture between different populations, we generated coancestry curves using MOSAIC (v1.2) [45]. To detect 2-way admixture events in EA and AAM, we chose MahMeri and Yakut, two extremely diverged but homogeneous populations (*F*_st_ = 0.067), as source populations of AAM- and EA-like ancestry respectively. We chose Mendriq, CheWong, Cambodian, Tu, and Jamatia as recipients (having ancestry from each of the source populations as a result of admixture) and estimated the admixture time for each of them by creating coancestry curves. The rate of decay in the curves was calculated which was equal to the number of generations since admixture took place.

The variation in dates of East Asian admixture with AAM was further estimated by keeping the recipient population the same (Cambodians). For this, MahMeri and Jehai, two homogeneous AAM populations, were chosen as source for AAM ancestry. Yakut, Dai, Naxi, Japanese, and Tu who were located in different geographical locations were chosen as EA ancestry source.

To estimate admixture between AAI and ASI, Birhor or Korwa and Paniya were selected respectively as references and Kadar or Ho as recipient. For AAI and TB admixture, Birhor and Jamatia were chosen as references for AAI and ATB ancestry respectively and Tharu as the admixed recipient.

### D statistics analysis

The tree topology of our D statistics analysis is shown in Fig. 3, where we wanted to identify if there was gene flow between test populations W, Y, and Z. We used Mbuti Pygmies from the HGDP dataset as X which had no evidence of gene flow with any of the other three populations used in the tree and therefore would not influence the test statistics estimate. A negative *Z* score value was indicative of gene flow between W and Z while a positive *Z* score would indicate gene flow between W and Y. Absolute *Z* score value greater than 3 was considered to be significant. We estimated D statistics using ADMIXTOOLS [41]. First we computed D statistics values keeping all subpopulations of AAI as Y, all subgroups of AAM and TB as Z and Cambodian as W. We repeated the same by replacing Cambodians with Dai subgroups while keeping the rest of the branches unchanged.

### Admixed segment length calculation

Local ancestry estimation was done using RFMix [44] version 1.5.4, to identify regions of genomes of both AAM and TB, representing different ancestries. The ancestry was estimated in three representative TB populations (Tripuri, Tharu, and Manipuri-Brahmin) and five representative AAM populations (CheWong, Bateq, Mendriq, Kintaq, and Jehai). Jamatia of ATB were used as a reference to infer the ATB like ancestry within ATB while MahMeri of AAM was used as a reference to infer the AAM-like ancestry within AAM. A few Southern EA subpopulations (Yizu, Han, Dai, Cambodian, and Naxi) were used as reference to infer Southern EA ancestry in each of the TB and AAM subgroups under investigation. The method identifies tracts of reference ancestry in each phased individual chromosome of the populations under investigation (here 3 TB and 5 AAM subgroups). Once segments belonging to these reference populations were identified in AAM and ATB, their length was calculated and the distribution of these lengths was observed by plotting histograms.

### Genetic geographic correlation test

The longitude and latitude coordinates of each subgroup of mainland India and Malaysia were obtained. Haversine method was used to determine the geographical distance between each subgroup. The weighted *F*_st_ values and the geographic distances were used in the form of a matrix to estimate the Pearson correlation using the Mantel test.

In a separate analysis, the non-AAI ancestry was masked using RFMix from the AAI individuals. For this, Birhors were kept as reference for AAI ancestry while TB and ASI represented their respective ancestry. The genomes of the rest of the AAI subgroups (Korwa, Gond, Ho, and Santhal) were masked for the ASI and TB like ancestry using RFMix. Similarly EA ancestry was masked in AAM subgroups (Bateq, Kintaq, and Mendriq), considering MahMeri, CheWong, and Jehai as AAM-like ancestry and all EA population as EA ancestry. The genomes of the rest of the subgroups from mainland India and Malaysia remained same. For the unmasked and newly masked genomes, new pairwise weighted *F*_st_ was calculated using PLINK. These new weighted *F*_st_ values and the same geographic distance were used to perform the Mantel test and to calculate the Pearson correlation.

### Effective population and population split time

Using “McEvoy” method implemented in NeON [46], effective population size (N_e_) over time was estimated for all subgroups of AAI, AAM, ANS, TB, and EA. *F*_st_ values previously calculated using PLINK version 1.9, between subgroups of AAI and AAM, were subsequently used to estimate population divergence time between them using the method described by McEvoy et al. [48] and UPGMA tree was generated on these population divergence times using R package “phangorn” [76].

### Ancient genome analysis

We also analyzed 43 ancient genomes from Southeast Asia [49, 50] spanning a period of nearly 8000 years ago (YA) to 200 YA. Out of these, 18 individuals were obtained from Lipson et al. [49], and the remaining 25 from McColl et al. [50]. We merged the genotypes of this dataset with the genotypes of our 1451 individuals. This dataset had 315,392 SNPs. We first performed PCA on these 43 ancient genomes along with AAI, AAM, TB, and a few East Asian populations (Han, Dai, Naxi, Yizu, and Cambodians) using EIGENSOFT package.

Using AAI, AAM, TB, and the same East Asian populations, we ran ADMIXTURE (v1.3.0) to calculate the CVE. Once the best *K* value was obtained, we projected the 43 ancient genomes on the inferred ancestries (using the *–P* parameter).

In a separate D statistics analysis of the form *D(((Z,Y)W)X*), we used all AAM subpopulations as Z while W and X remained East Asian and Mbuti Pygmies respectively. For Y, we used 5 ancient genome groups (the details of the groups are in the main text) namely Anc_8K, Anc_4K, Anc_3K, Anc_2K, and Anc_R one at a time.

## Supplementary Information

**Additional file 1:** **Supplementary Figure 1.** ADMIXTURE analysis on populations from mainland India, Malaysia, East Asians (EA) of HGDP and Central South Asians (CSA) of HGDP. **Supplementary Figure 2.** IBS and ROH distribution. **Supplementary Figure 3.** Haplotype based clustering using fineSTRUCTURE on the mainland Indian and Malaysian population. **Supplementary Figure 4.** F_st_ estimation. **Supplementary Figure 5.** Genetic distance and geographic distance correlation **Supplementary Figure 6.** Population separation, drift and geneflow. **Supplementary Figure 7.** IBD estimation between AAM and AAI. **Supplementary Figure 8.** IBD estimation between TB and AAI. **Supplementary Figure 9.** IBD estimation between AAM and TB. **Supplementary Figure 10.** Admixed segment length. **Supplementary Figure 11.** Change in effective population size. **Supplementary Figure 12.** The age distribution of the ancient genomes, **Supplementary Figure 13.** ADMIXTURE analysis on all subgroups of AAI, AAM and TB and a few subpopulations of EA. **Supplementary Figure 14.** Ancestry correlation . **Supplementary Figure 15.** D statistics value estimation.

**Additional file 2: Supplementary Table 1.** Classification of populations used in the study. **Supplementary Table 2.** Values of outgroup *f3*(Mbuti Pygmy; X, Y) statistics. **Supplementary Table 3.** Minimum maximum and total number of bases and total number of discrete segments of IBD shared. **Supplementary Table 4.** Ancestry proportion estimation. **Supplementary Table 5.** D statistics with AAM, AAI, Southern-East Asians and an African population. **Supplementary Table 6.** Admixture time estimation. **Supplementary Table 7.** Metadata on the 43 ancient genomes. **Supplementary Table 8.** D statistics with Ancient genomes, AAM, Southern-East Asians and an African population.

## Data Availability

The dataset used in the study comprising of 1451 individuals (367 from India, 144 from Malaysia, and 940 from Human Genome Diversity Project) is deposited at NIBMG repository under the directory “BMCB2021” [76]. The HGDP dataset from the original study [53] is also separately available at [77]. Data of 18 ancient genomes from the original study [49] are available in [78] and of 25 ancient genomes from the original study [50] are available through the European Nucleotide Archive [79] under the accession number (PRJEB26721).

## Abbreviations

AA: Austroasiatic
AAI: Austroasiatics of India
AAM: Austroasiatics of Malaysia
AMH: Anatomically modern humans
ANI: Ancestral North Indian
ASI: Ancestral South Indian
ATB: Ancestral Tibeto-Burman
CSA: Central South Asia
EA: East Asia
HGDP: Human Genome Diversity Project
IBD: Identity by descent
IBS: Identity by state
MSEA: Mainland Southeast Asia
PC: Principal Component
PCA: Principal Components Analysis
SNP: Single Nucleotide polymorphism
SEA: Southeast Asia
YA: Years ago
YO: Years old
