# Publisher Correction to: Insights into the demographic history of Asia from common ancestry and admixture in the genomic landscape of present-day Austroasiatic speakers

**DOI:** 10.1186/s12915-021-01174-2

**Published:** 2021-11-04

**Authors:** Debashree Tagore, Farhang Aghakhanian, Rakesh Naidu, Maude E. Phipps, Analabha Basu

**Affiliations:** 1 National Institute of Biomedical Genomics, Kalyani, 741251 India; 2 Oklahoma Medical Research Foundation, Genes and Human Disease Program, 825 NE 13th Street, Oklahoma City, OK 73104 USA; 3 Genomics Facility, School of Science, Monash University Malaysia, Jalan Lagoon Selatan, 47500 Bandar Sunway, Selangor Malaysia; 4 Jeffrey Cheah School of Medicine and Health Sciences, Monash University Malaysia, Jalan Lagoon Selatan, 47500 Bandar Sunway, Selangor Darul Ehsan Malaysia

**Publisher Correction to: BMC Biol 19, 61 (2021)**

**https://doi.org/10.1186/s12915-021-00981-x**

Following publication of the original article [1], an error was identified in the ‘Availability of data and materials’ section due to a typesetting mistake: in the first sentence of this section, a citation to reference 76 was present, instead of a citation to reference 72.

The original article [1] has been updated. The publisher apologises to the authors and readers for the inconvenience caused by this mistake.
