# Supplementary material for: Insights into the demographic history of Asia from common ancestry and admixture in the genomic landscape of present-day Austroasiatic speakers
Source: BMC Biol. 2021 Mar 29;19:61. doi: 10.1186/s12915-021-00981-x (PMC8008685; doi:10.1186/s12915-021-00981-x)
Supplement: Supplementary file 1 — Additional file 1: Supplementary Figure 1. ADMIXTURE analysis on populations from mainland India, Malaysia, East Asians (EA) of HGDP and Central South Asians (CSA) of HGDP. Supplementary Figure 2. IBS and ROH distribution. Supplementary Figure 3. Haplotype based clustering using fineSTRUCTURE on the mainland Indian and Malaysian population. Supplementary Figure 4. Fst estimation. Supplementary Figure 5. Genetic distance and geographic distance correlation Supplementary Figure 6. Population separation, drift and geneflow. Supplementary Figure 7. IBD estimation between AAM and AAI. Supplementary Figure 8. IBD estimation between TB and AAI. Supplementary Figure 9. IBD estimation between AAM and TB. Supplementary Figure 10. Admixed segment length. Supplementary Figure 11. Change in effective population size. Supplementary Figure 12. The age distribution of the ancient genomes, Supplementary Figure 13. ADMIXTURE analysis on all subgroups of AAI, AAM and TB and a few subpopulations of EA. Supplementary Figure 14. Ancestry correlation . Supplementary Figure 15. D statistics value estimation. [file 12915_2021_981_MOESM1_ESM.pdf]

Table of contents in Additional file 1:

Supplementary Figure 1: ADMIXTURE analysis on populations from mainland India, Malaysia, East Asians (EA) of HGDP and Central South Asians (CSA) of HGDP: (a) Cross Validation Error graph on unpruned data and LD pruned data (SNPs pruned at $r^2 = 0.1$ and $r^2 = 0.5$) . **(b)** ADMIXTURE cluster graph on the same set of population for K=2 to K=9. The abbreviations used are according to Supplementary Table 1 in Additional file 2. **(c)** Scatter plot for the ancestry proportion of each individual versus standard error for each estimate. The colors correspond to ancestry colors as in (b)

Supplementary Figure 2: IBS and ROH distribution: Distribution of **(a)** Proportion of Identity by state (IBS) shared between individuals of different subpopulations denoted in the x axis. **(b)** Proportion of Runs of Homozygosity (ROH) within each individual of the same set of subpopulations

Supplementary Figure 3: Haplotype based clustering using fineSTRUCTURE on the mainland Indian and Malaysian population: The populations included are: ANI (blue), ASI (green), AAI (purple) and ATB (orange) and Malaysian population(both AAM and ANS ; color brown).

Supplementary Figure 4: F_{st} estimation: Heatmap generated on the estimated weighted F_{st} values between subpopulations belonging to mainland Indian and Malaysian populations. The different subgroups are labelled on the right and the bottom of the heatmap. Light yellow colour represents low F_{st} value while dark red represents high F_{st} value.

Supplementary Figure 5: Genetic distance and geographic distance correlation: (a) for Indian mainland and Malaysian population (b) for AAI and AAM without masking non Austroasiatic ancestry within them and (c) for AAI and AAM

after masking non Austroasiatic ancestry within them

Supplementary Figure 6: Population separation, drift and geneflow: (a) Maximum likelihood tree generated by Treemix on the subpopulations of AAI, ATB, AAM and ANS with Mbuti pygmies as outgroup and assuming 3 migration events. **(b)** Residuals of the **m**aximum likelihood tree generated by Treemix in (a)

Supplementary Figure 7: IBD estimation between AAM and AAI: Estimated IBD segment length distribution between each AAM subgroup and **(a)** Birhors, **(b)** Gond **(c)** Ho **(d)** Korwa and **(e)** Santhal. Each figure has six subplots one for each AAM subgroup.

Supplementary Figure 8: IBD estimation between TB and AAI: Estimated IBD segment length distribution between each TB subgroup and **(a)** Birhors, **(b)** Gond **(c)** Ho **(d)** Korwa and **(e)** Santhal. Each figure has four subplots one for each TB subgroup.

Supplementary Figure 9: IBD estimation between AAM and TB: Estimated IBD segment length distribution between each AAM subgroup and **(a)** Jamatia, **(b)** Tripuri **(c)** Manipuri Brahmin **(d)** Tharu. Each figure has six subplots one for each AAM subgroup.

Supplementary Figure 10: Admixed segment length: Distribution of length of contiguous segment of EA ancestry in TB and AAM.

Supplementary Figure 11: Change in effective population size: N_e was estimated for over 1000 generations in all subgroups of **(a)** AAM **(b)** AAI, **(c)**ANS, **(d)** TB and **(e)** EA

Supplementary Figure 12: The age distribution of the ancient genomes: Each dot represents an ancient genome, with the standard error of the estimate (age of one sample, La898, not available).

Supplementary Figure 13: ADMIXTURE analysis on all subgroups of AAI, AAM and TB and a few subpopulations of EA: (a) Cross validation error **(b)** ADMIXTURE cluster graph for K=2 to K=4. The East Asian populations included in this analysis are: Han, Dai, Naxi, Yizu and Cambodian

Supplementary Figure 14: Ancestry correlation: Correlation graph of **(a)** proportion of EA ancestry with age of the ancient samples and their longitudinal positions **(b)** proportion of AAI ancestry with latitudinal and longitudinal positions of the ancient samples

Supplementary Figure 15: D statistics value estimation: Distribution of D statistics values of the form *D(((Z,Y)Cambodian)Mbuti Pygmies)*. The x axis represents the D values. The y axis represents the pair of populations belonging to Z and Y. The y axis labels and data points are colored as dark green, orange, cyan, blue and red for Y group belonging to Anc_R, Anc_2K, Anc_3K, Anc_4K and Anc_8K respectively.

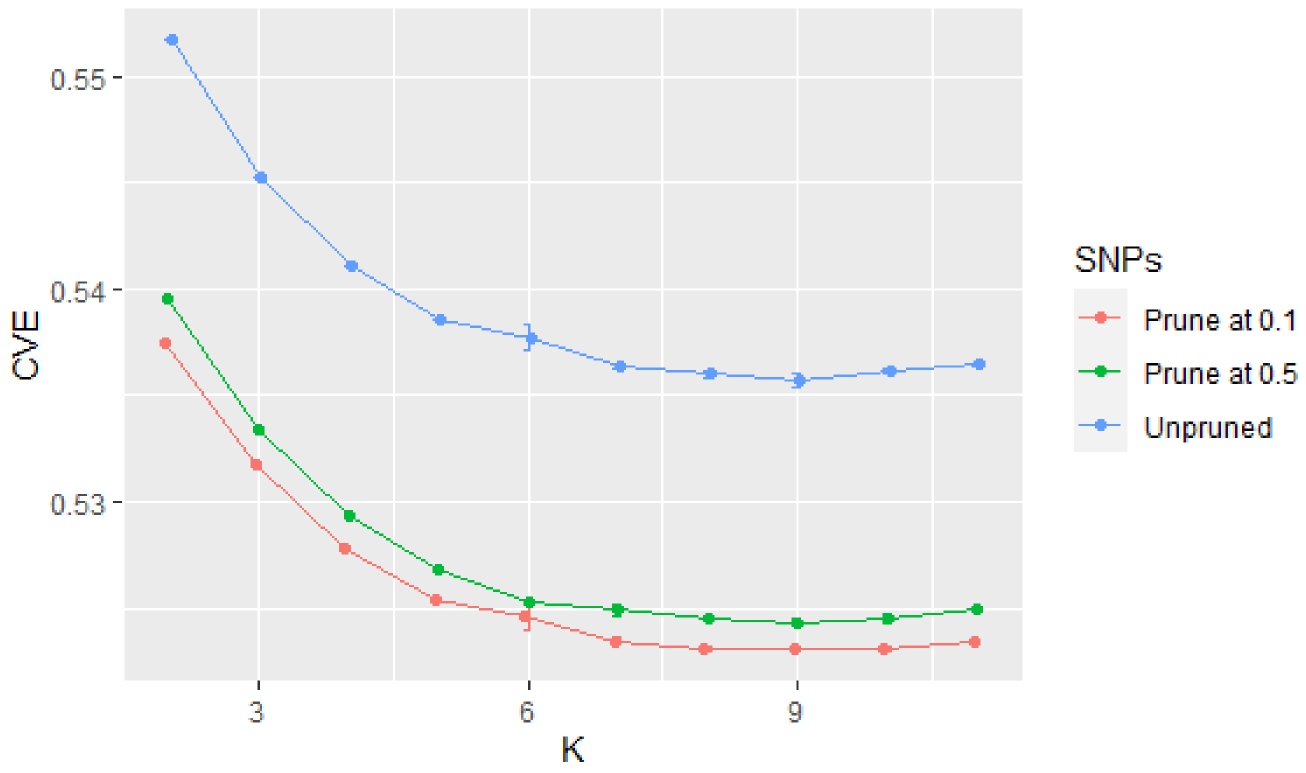

Supplementary Figure 1a: ADMIXTURE analysis on populations from mainland India, Malaysia, East Asians (EA) of HGDP and Central South Asians (CSA) of HGDP: Cross Validation Error graph on unpruned data and LD pruned data (SNPs pruned at r^2 = 0.1 and r^2 = 0.5) .

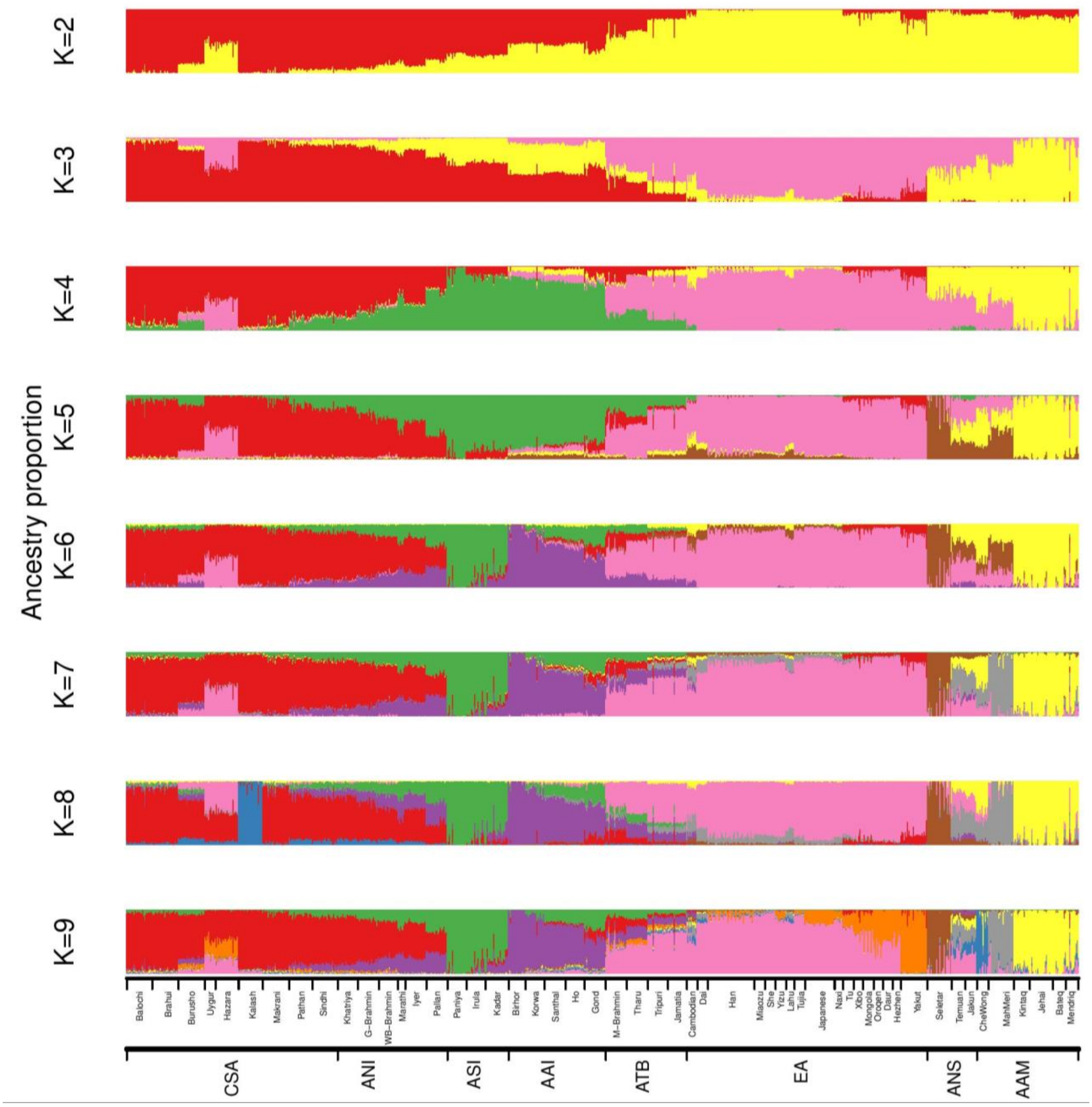

Supplementary Figure 1b: ADMIXTURE analysis on populations from mainland India, Malaysia, East Asians (EA) of HGDP and Central South Asians (CSA) of HGDP: ADMIXTURE cluster graph on the same set of population as in (a) for K=2 to K=9. The abbreviations used are according to Supplementary Table 1 in Additional file 2.

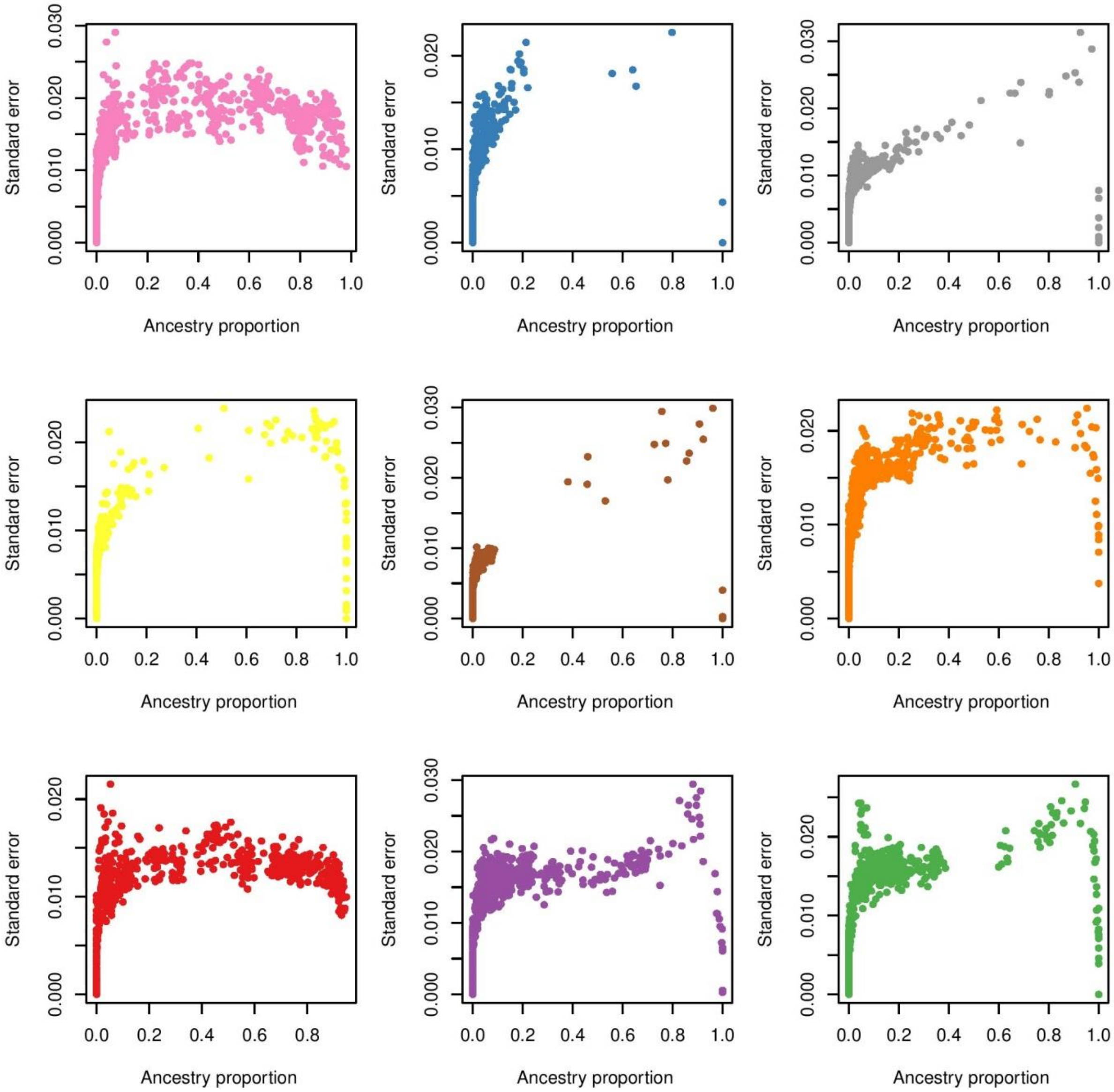

Supplementary Figure 1c: ADMIXTURE analysis on populations from mainland India, Malaysia, East Asians (EA) of HGDP and Central South Asians (CSA) of HGDP: Scatter plot for the ancestry proportion of each individual versus standard error of each estimate. The colors correspond to ancestry colors as in Supplementary Figure 1b

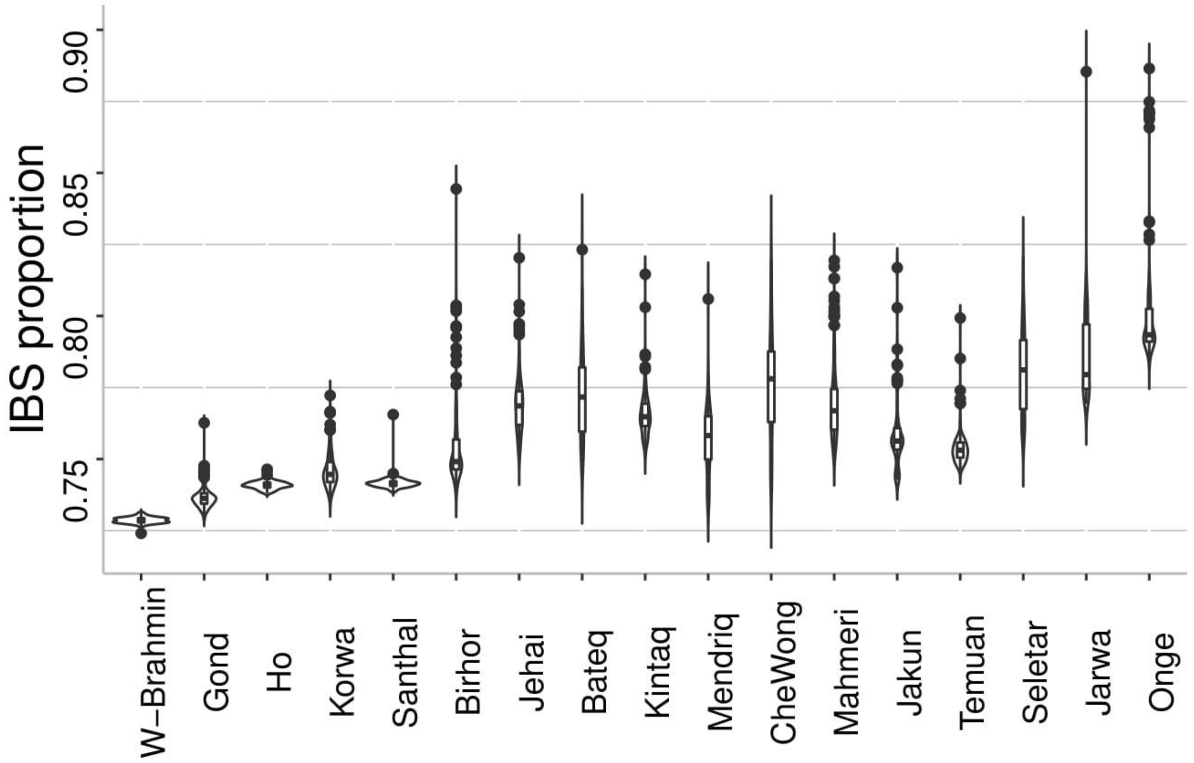

Supplementary Figure 2a: IBS and ROH distribution: Distribution of proportion of Identity by state (IBS) shared between individuals of different subpopulations denoted in the x axis.

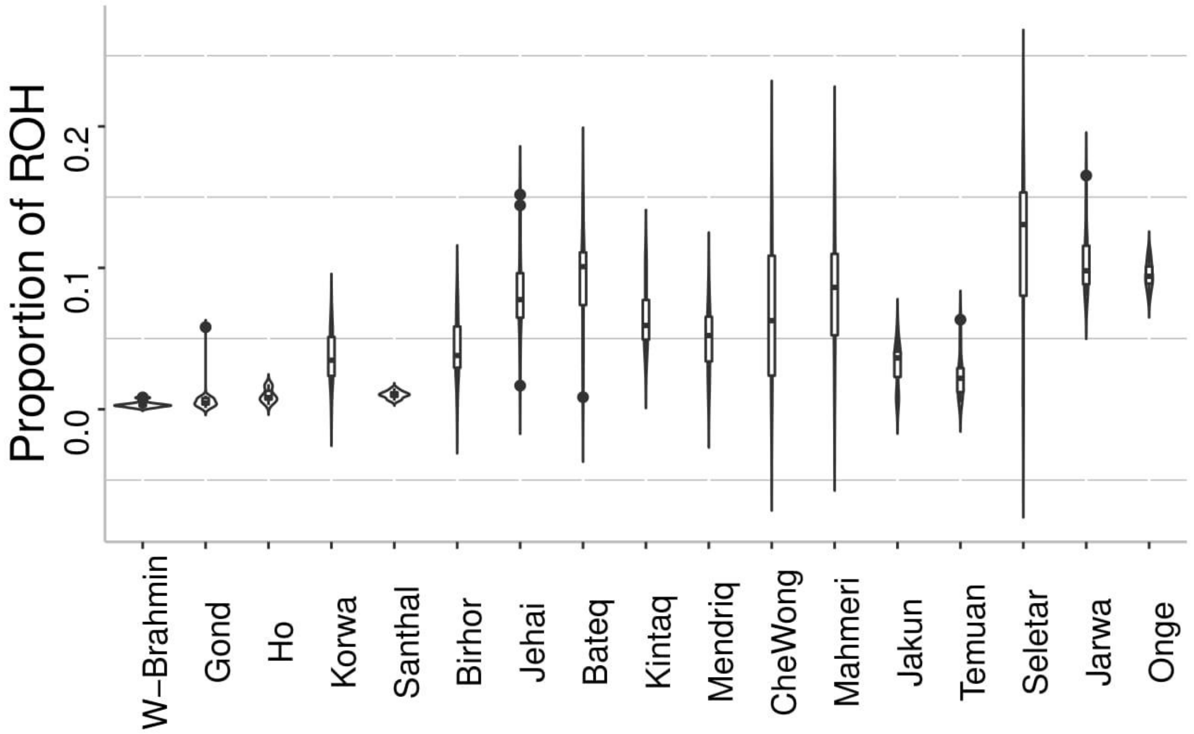

Supplementary Figure 2b: IBS and ROH distribution: Proportion of Runs of Homozygosity (ROH) within each individual of the same set of subpopulations.

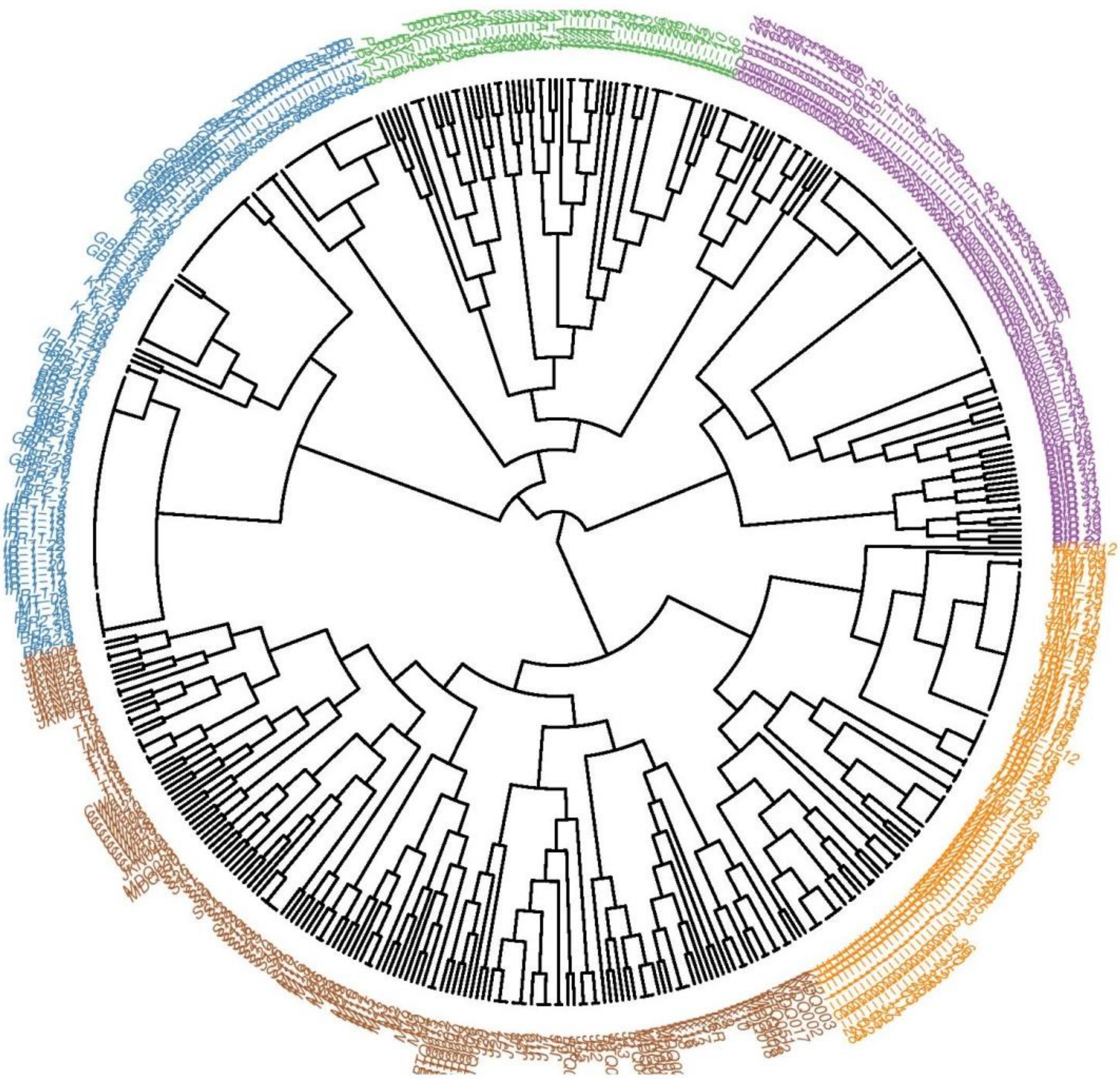

Supplementary Figure 3: Haplotype based clustering using fineSTRUCTURE on the mainland Indian and Malaysian population: The populations included are: ANI (blue), ASI (green), AAI (purple) and ATB (orange) and Malaysian population(both AAM and ANS ; color brown).

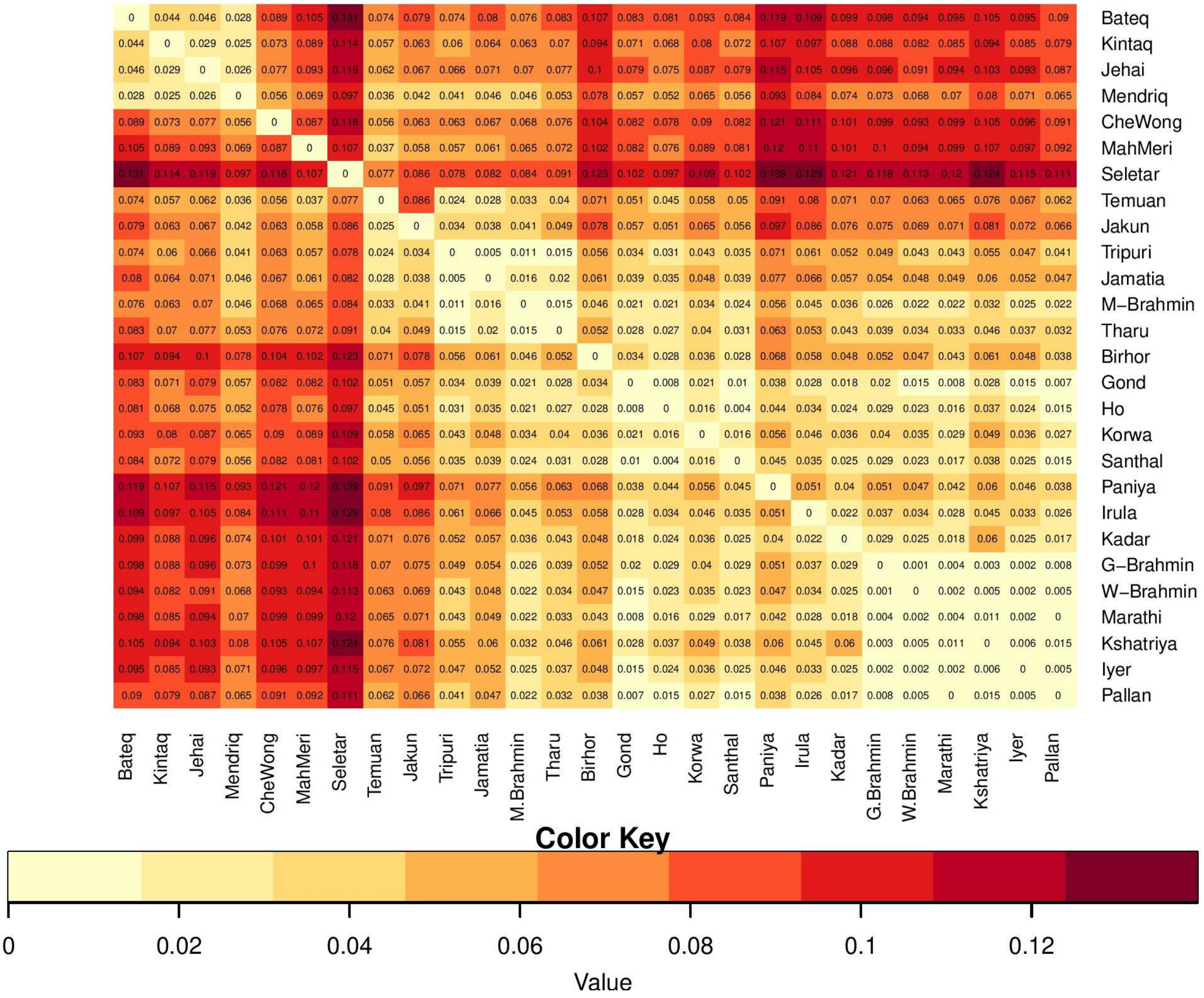

Supplementary Figure 4: F_{st} estimation: Heatmap generated on the estimated weighted F_{st} values between subpopulations belonging to mainland Indian and Malaysian populations. The different subgroups are labelled on the right and the bottom of the heatmap. Light yellow colour represents low F_{st} value while dark red represents high F_{st} value.

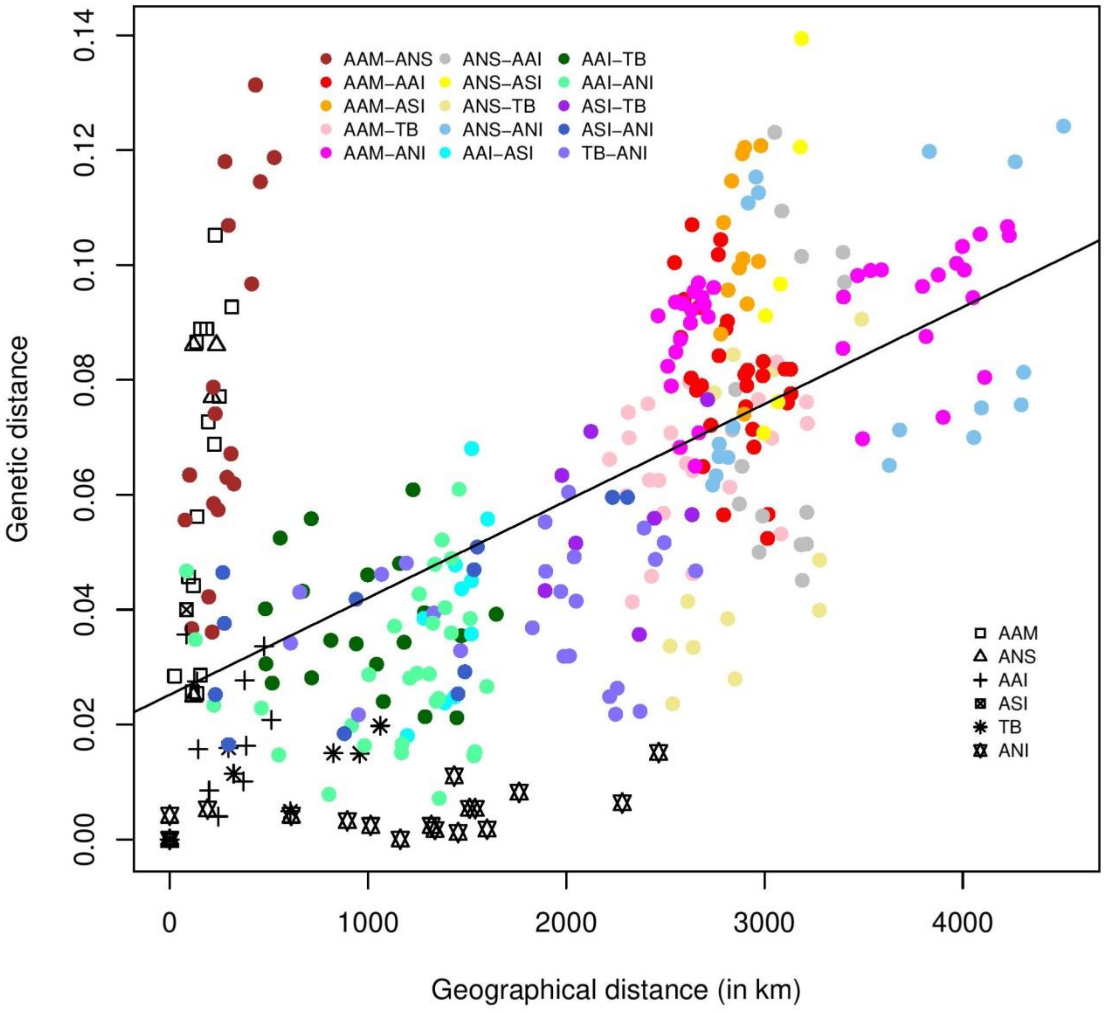

Supplementary Figure 5a: Genetic distance and geographic distance correlation for Indian mainland and Malaysian population

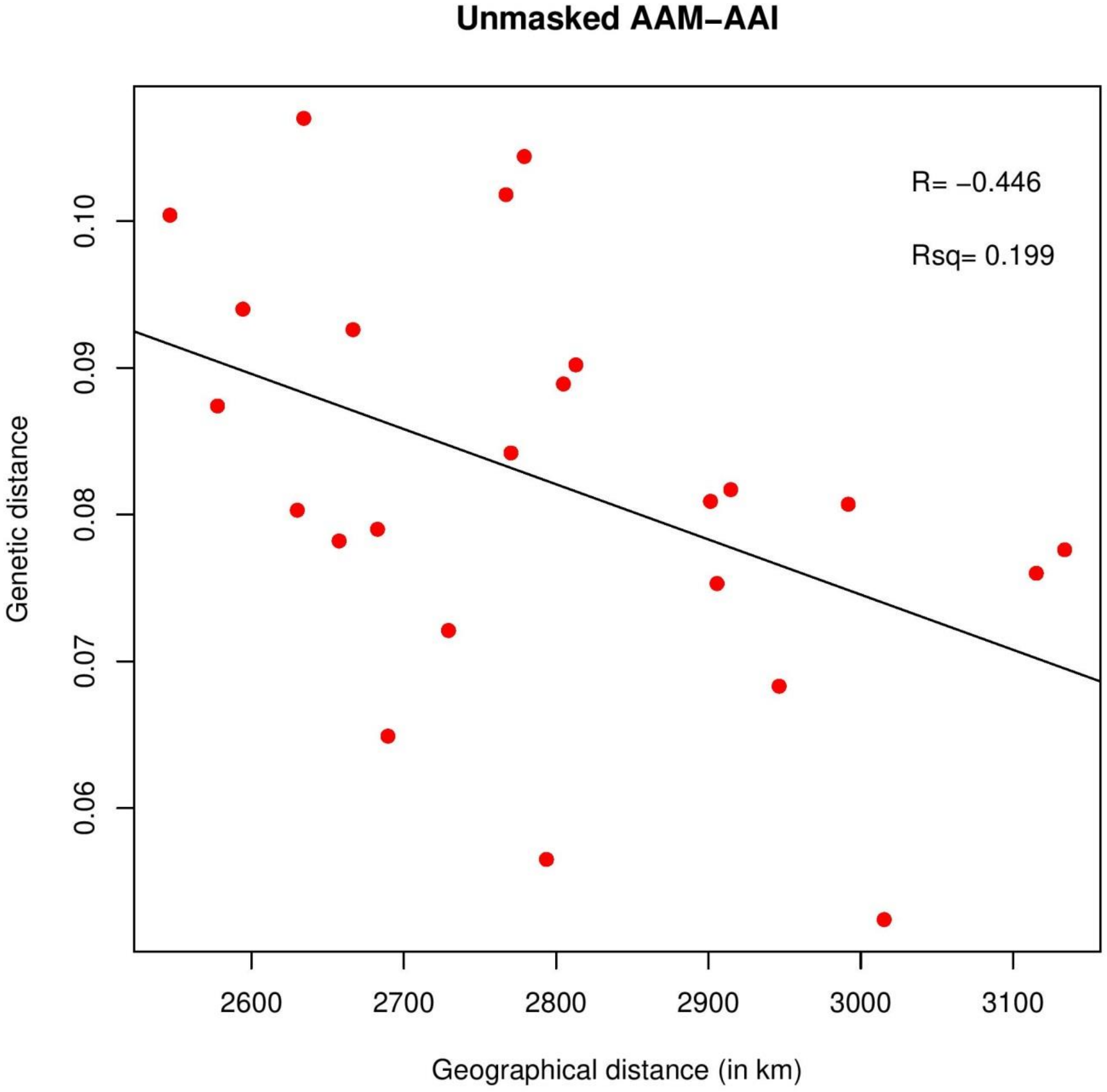

Supplementary Figure 5b: Genetic distance and geographic distance correlation: for AAI and AAM without masking non Austroasiatic ancestry within them.

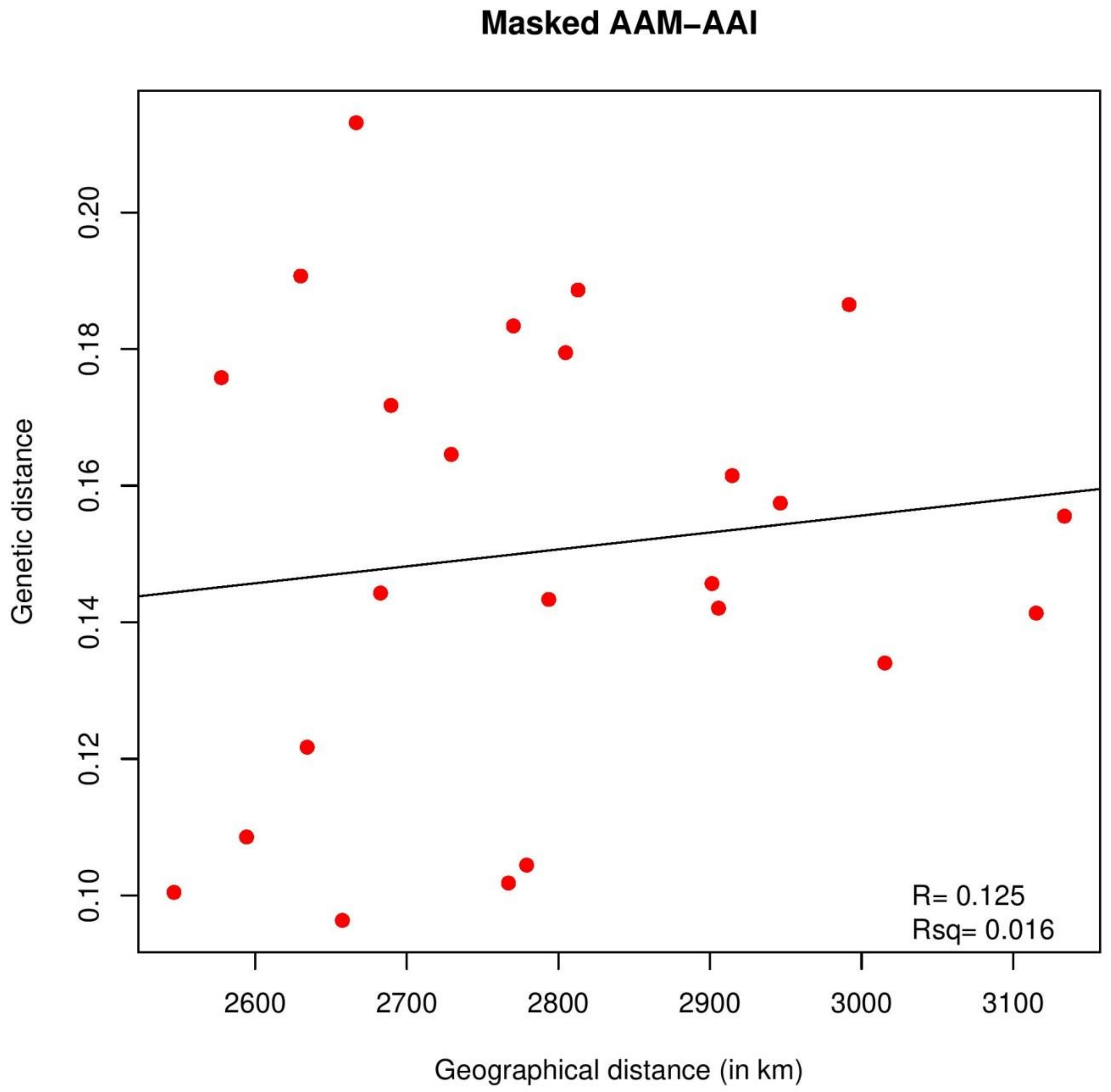

Supplementary Figure 5c: Genetic distance and geographic distance correlation: for AAI and AAM after masking non Austroasiatic ancestry within them

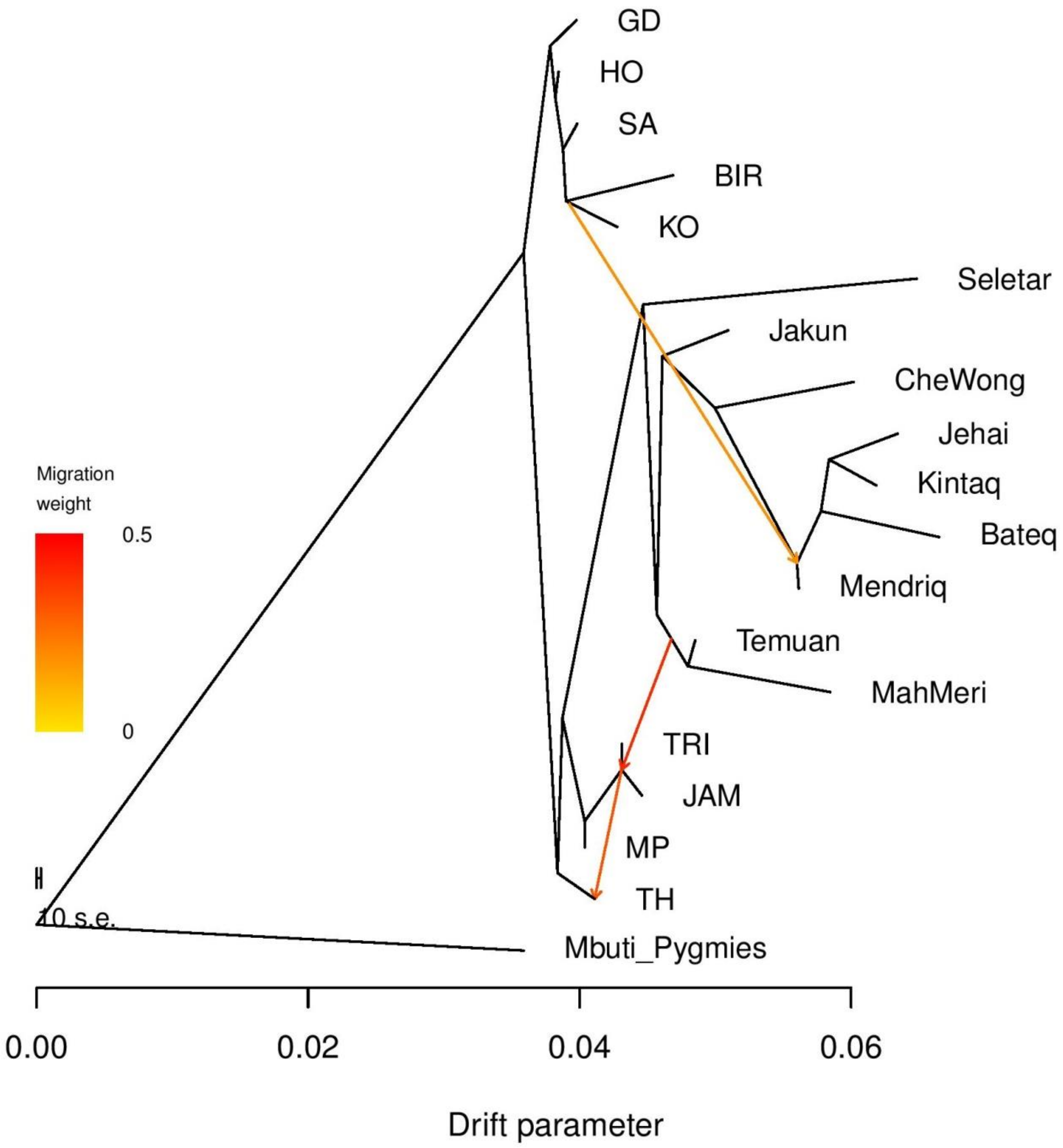

Supplementary Figure 6a: Population separation, drift and geneflow: Maximum likelihood tree generated by Treemix on the subpopulations of AAI, ATB, AAM and ANS with Mbuti pygmies as outgroup and assuming 3 migration events.

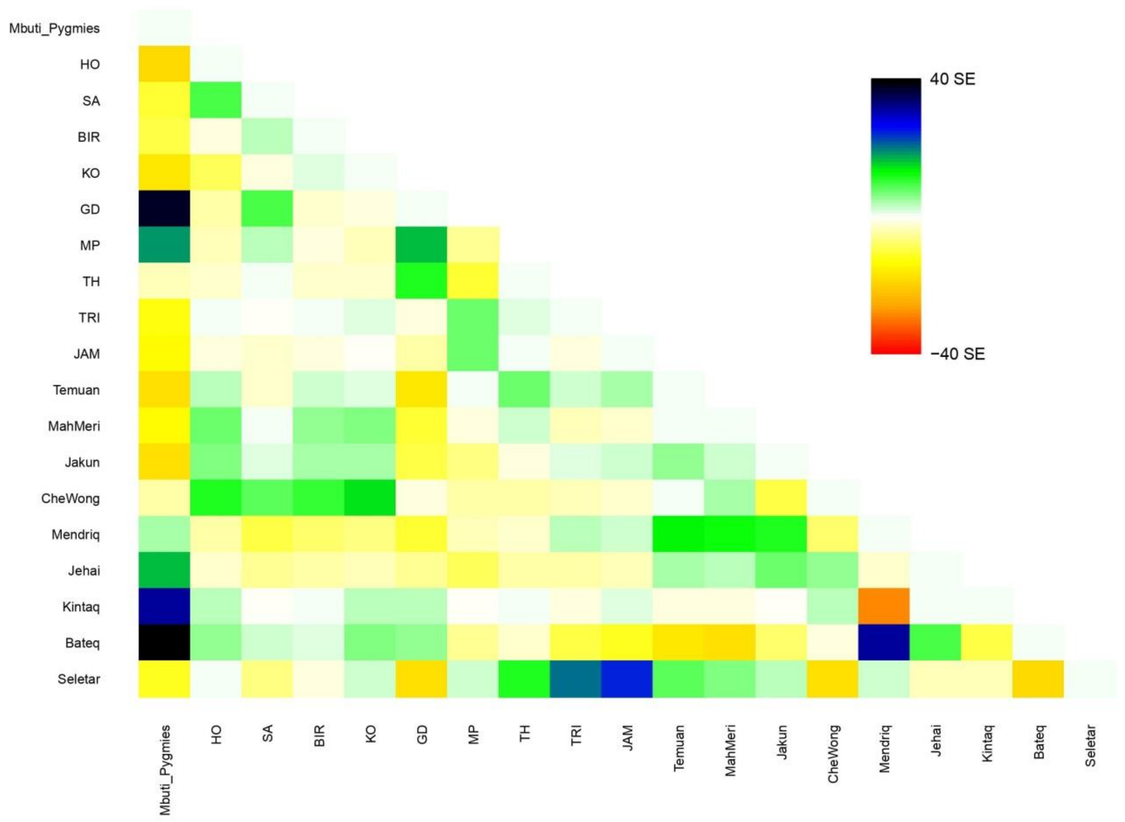

Supplementary Figure 6b: Population separation, drift and geneflow: Residuals of the **m**aximum likelihood tree generated by Treemix on the subpopulations of AAI, ATB, AAM and ANS with Mbuti pygmies as outgroup and assuming 3 migration events.

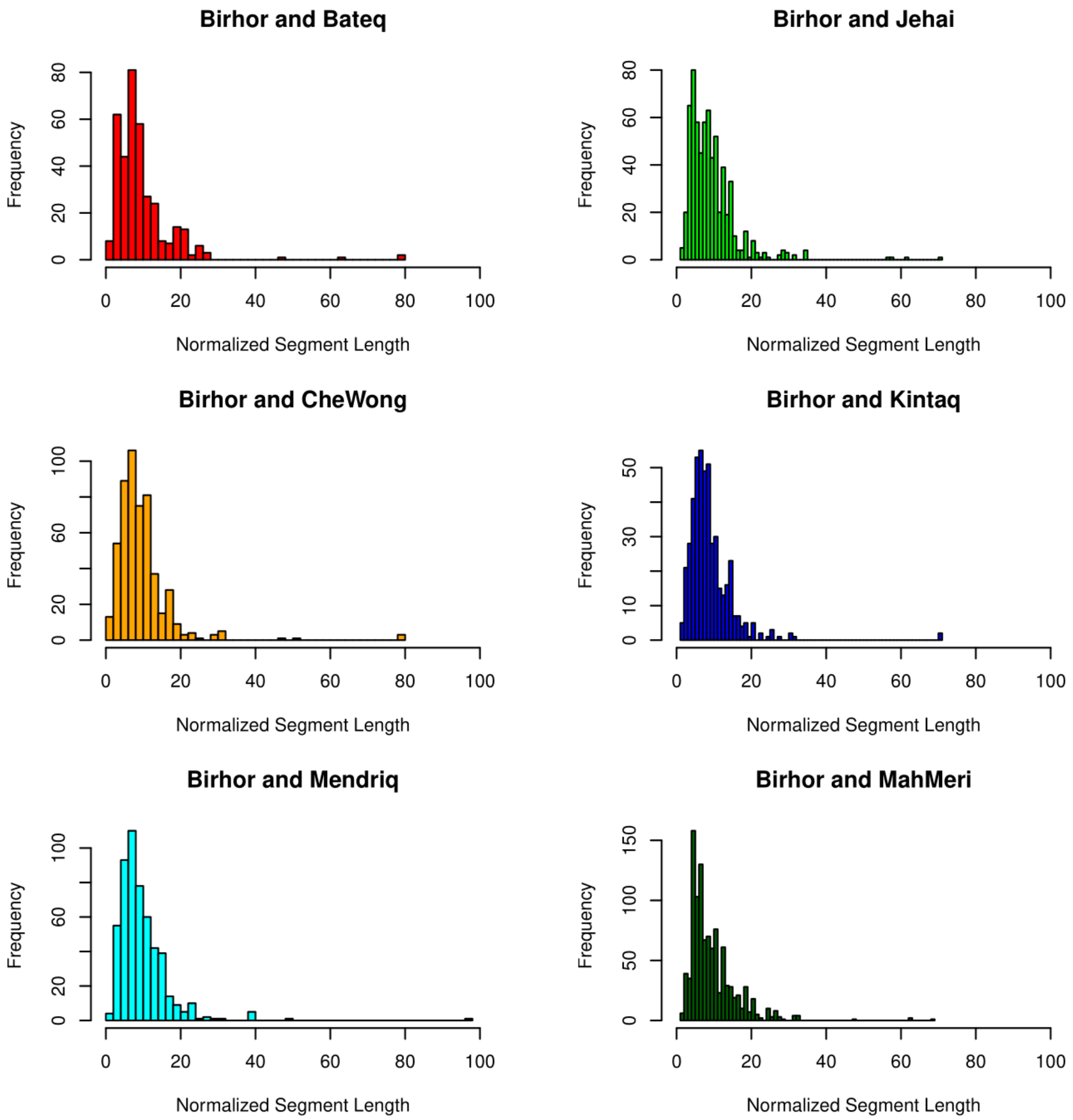

Supplementary Figure 7a: IBD estimation between AAM and AAI: Estimated IBD segment length distribution between each AAM subgroup and Birhor.

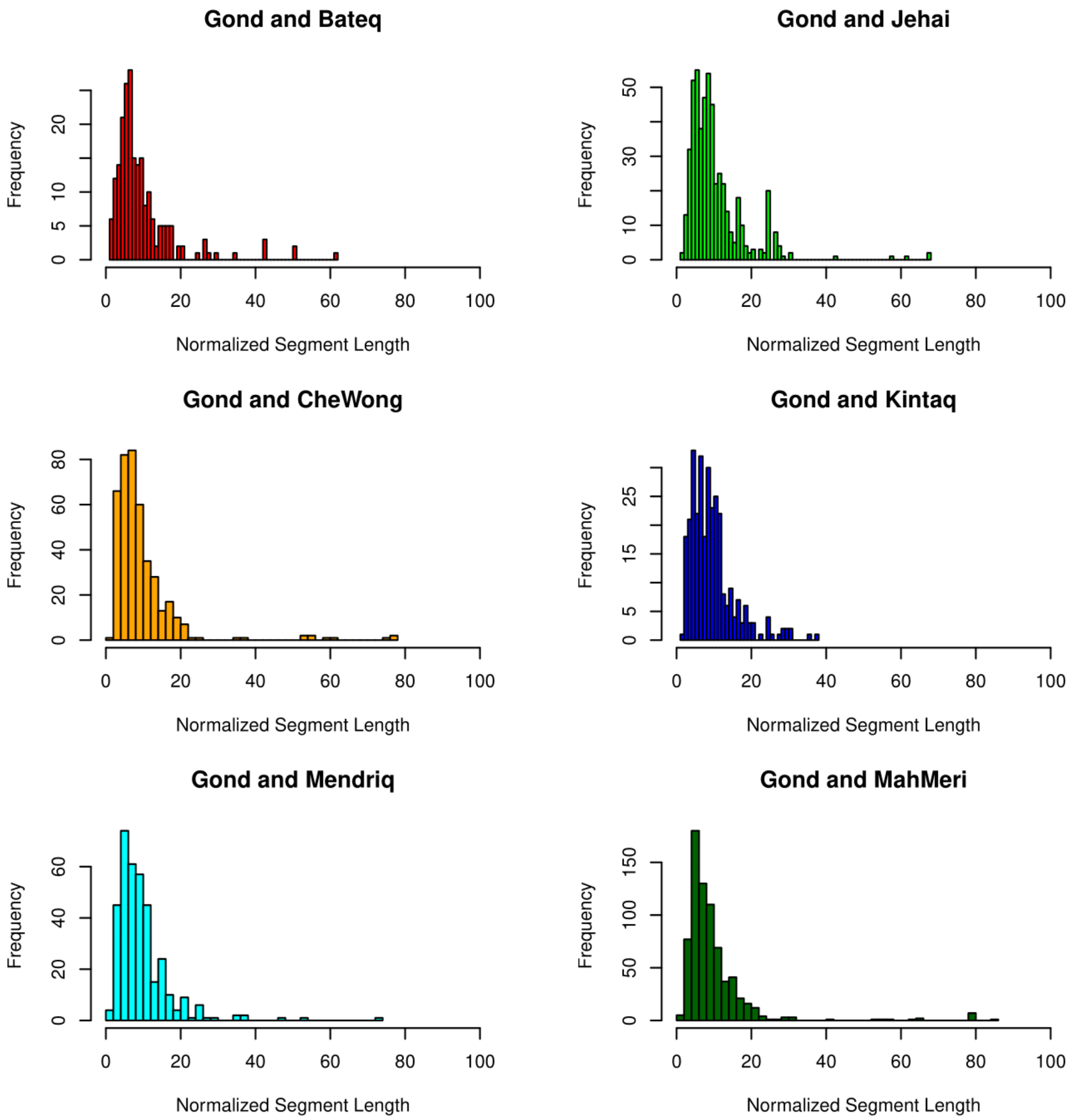

Supplementary Figure 7b: IBD estimation between AAM and AAI: Estimated IBD segment length distribution between each AAM subgroup and Gond.

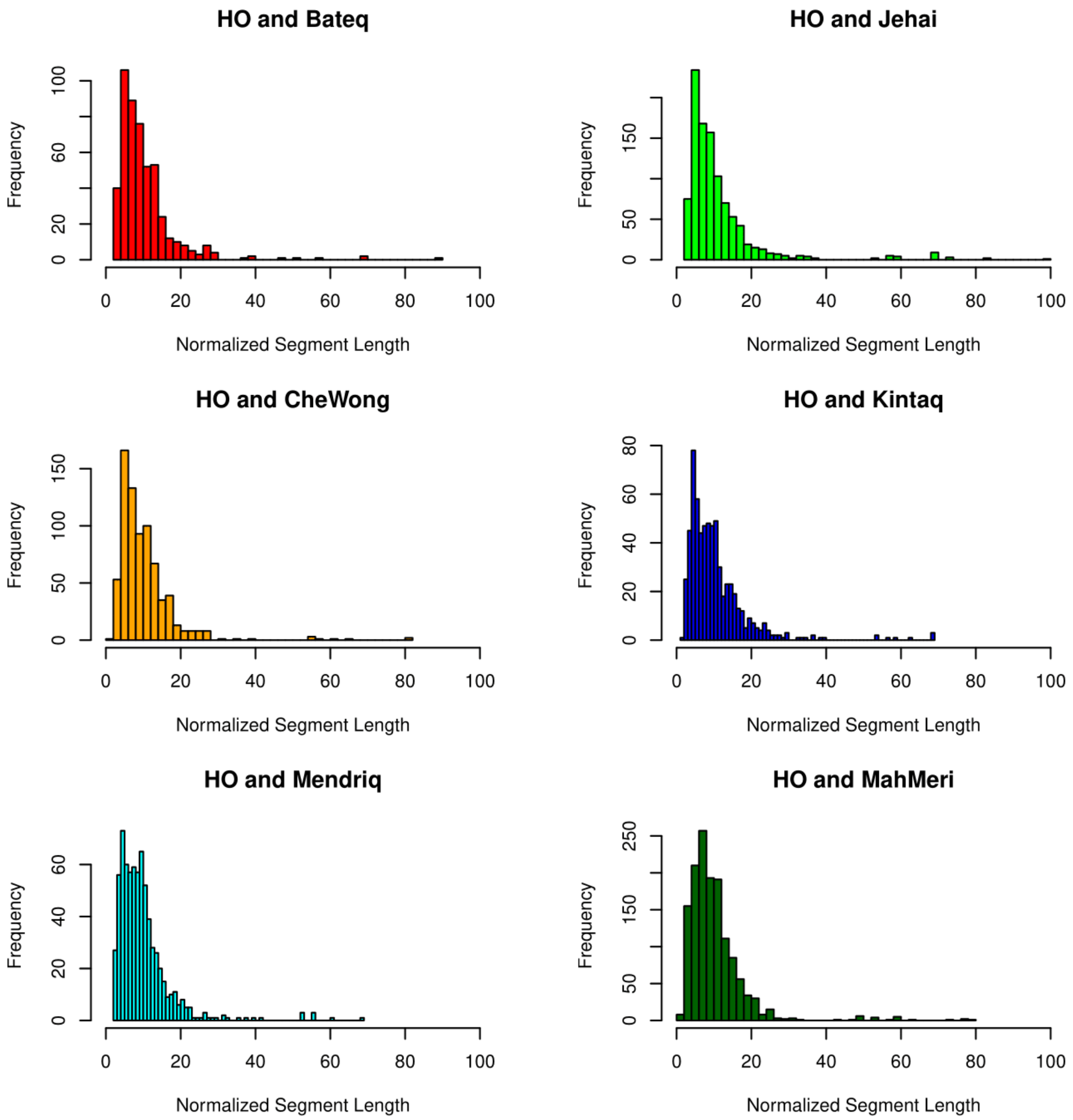

Supplementary Figure 7c: IBD estimation between AAM and AAI: Estimated IBD segment length distribution between each AAM subgroup and Ho.

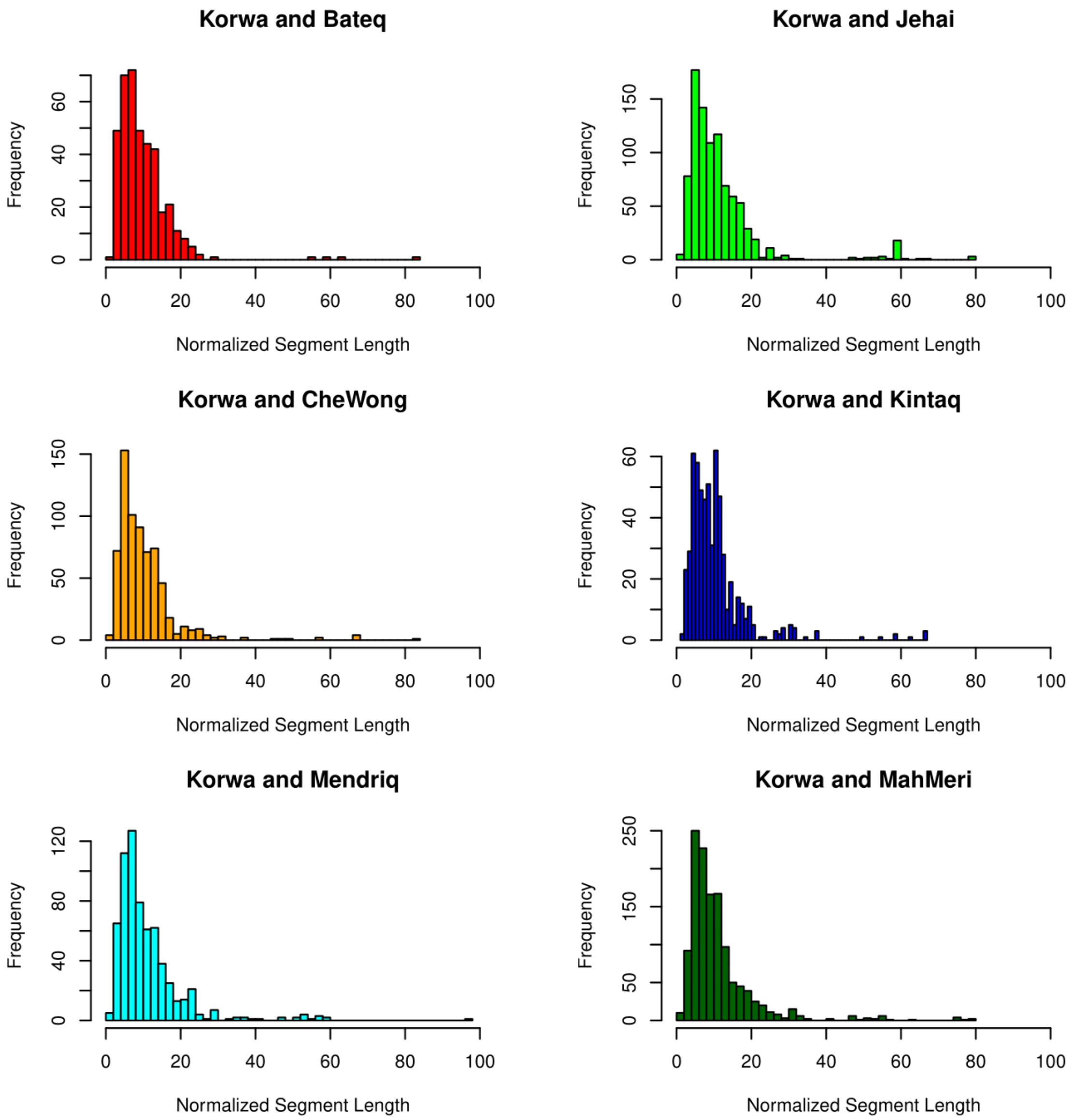

Supplementary Figure 7d: IBD estimation between AAM and AAI: Estimated IBD segment length distribution between each AAM subgroup and Korwa

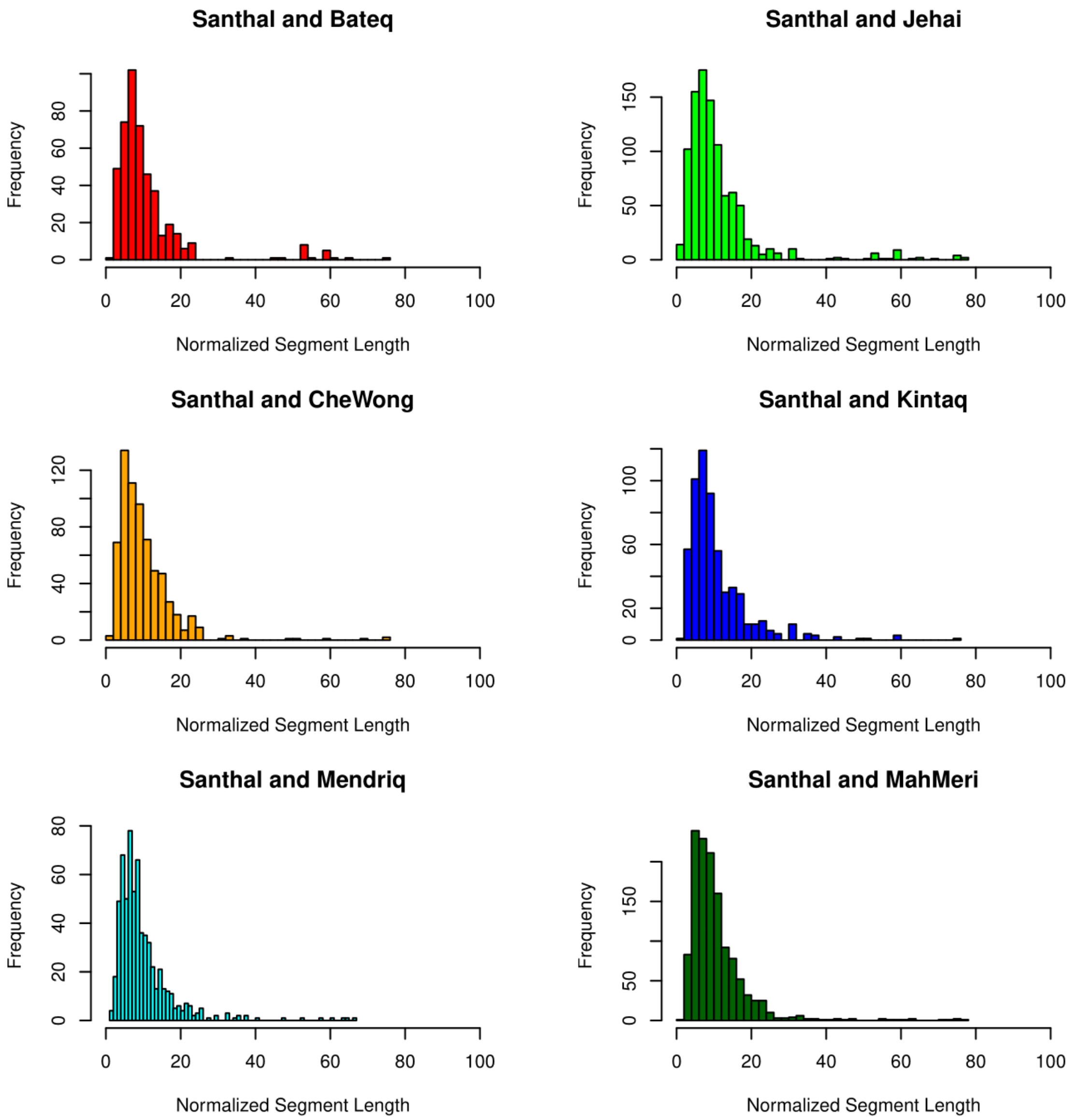

Supplementary Figure 7e: IBD estimation between AAM and AAI: Estimated IBD segment length distribution between each AAM subgroup and Santhal.

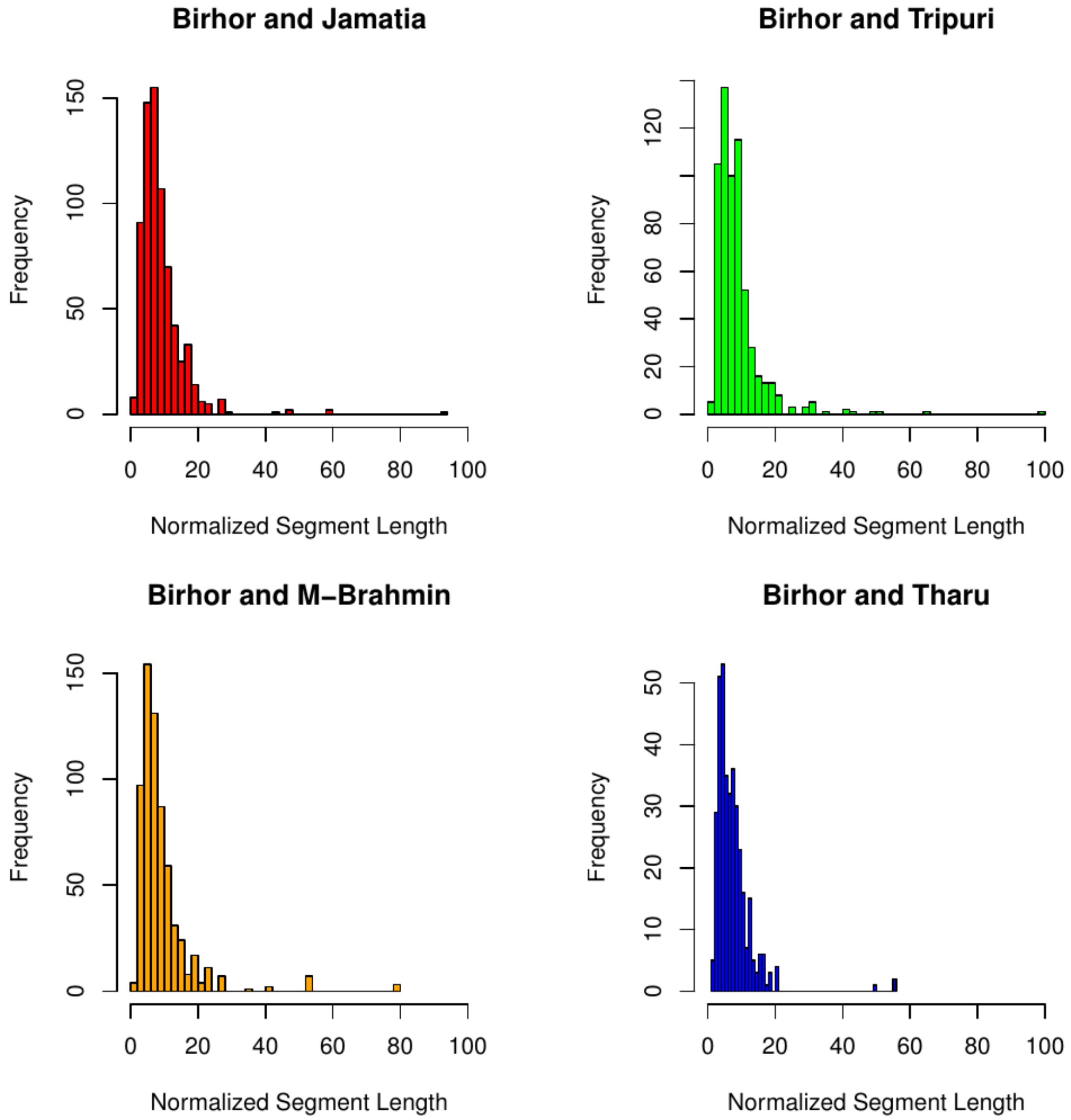

Supplementary Figure 8a: IBD estimation between TB and AAI: Estimated IBD segment length distribution between each TB subgroup and Birhor.

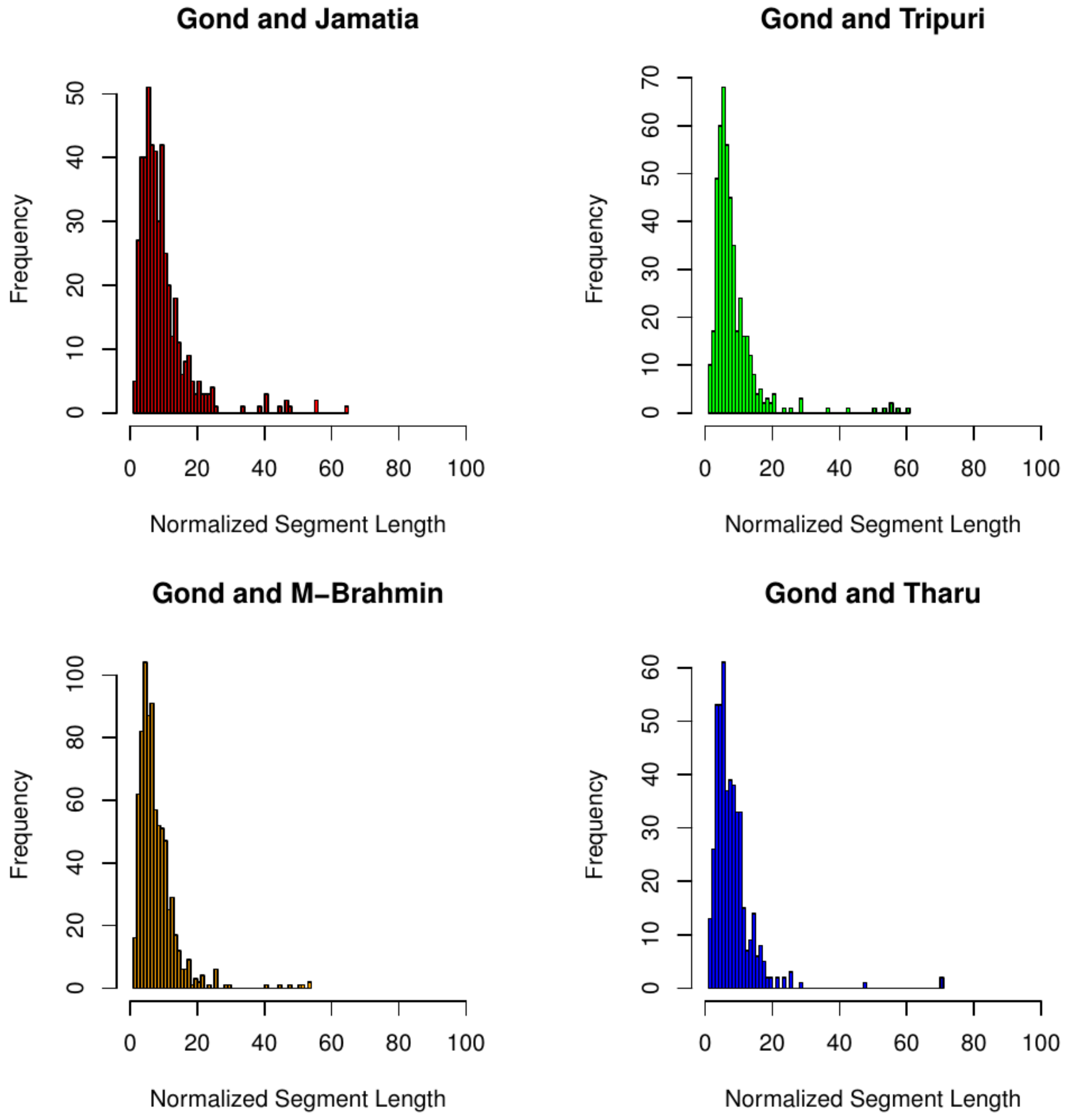

Supplementary Figure 8b: IBD estimation between TB and AAI: Estimated IBD segment length distribution between each TB subgroup and Gond.

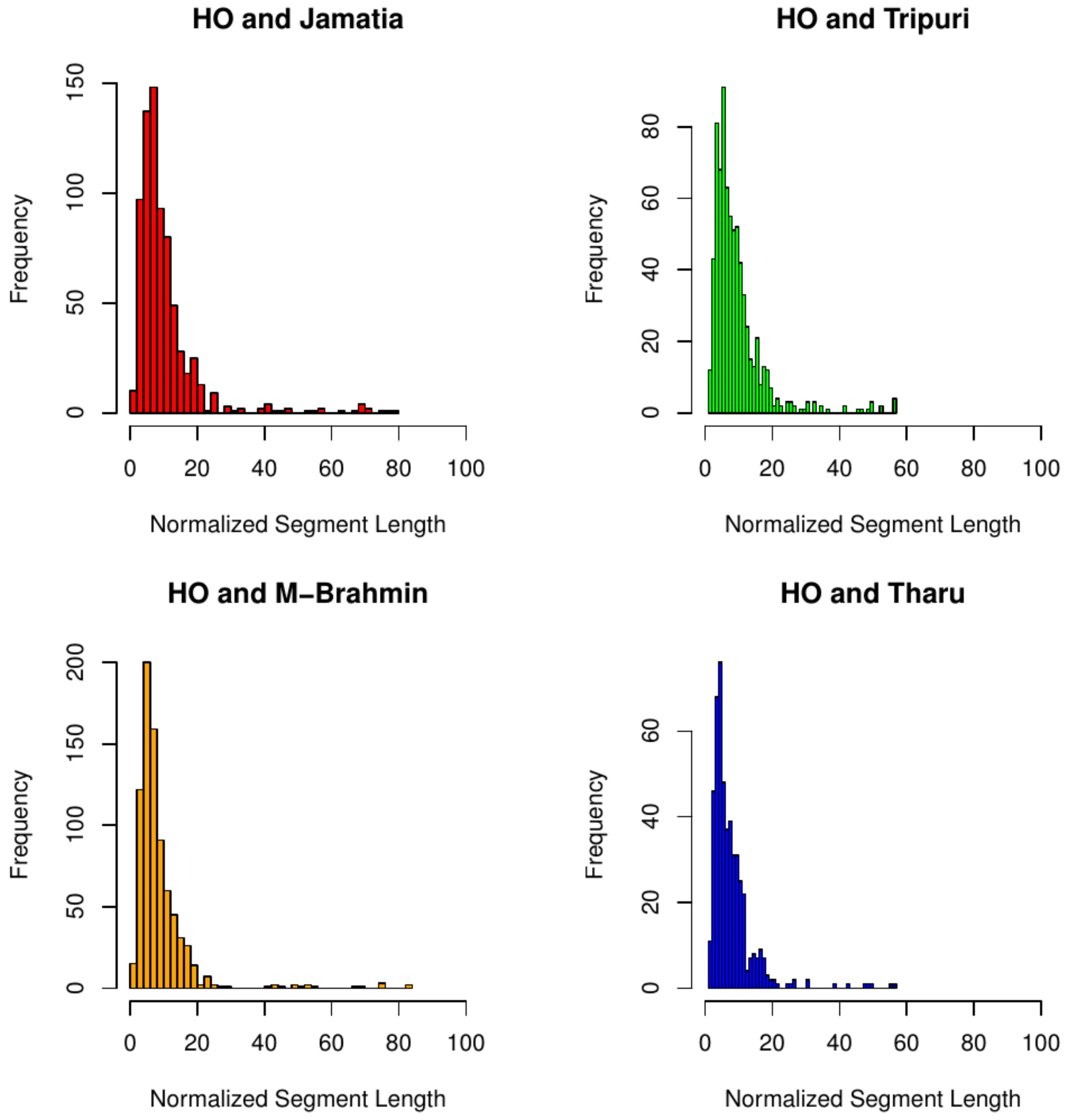

Supplementary Figure 8c: IBD estimation between TB and AAI: Estimated IBD segment length distribution between each TB subgroup and Ho.

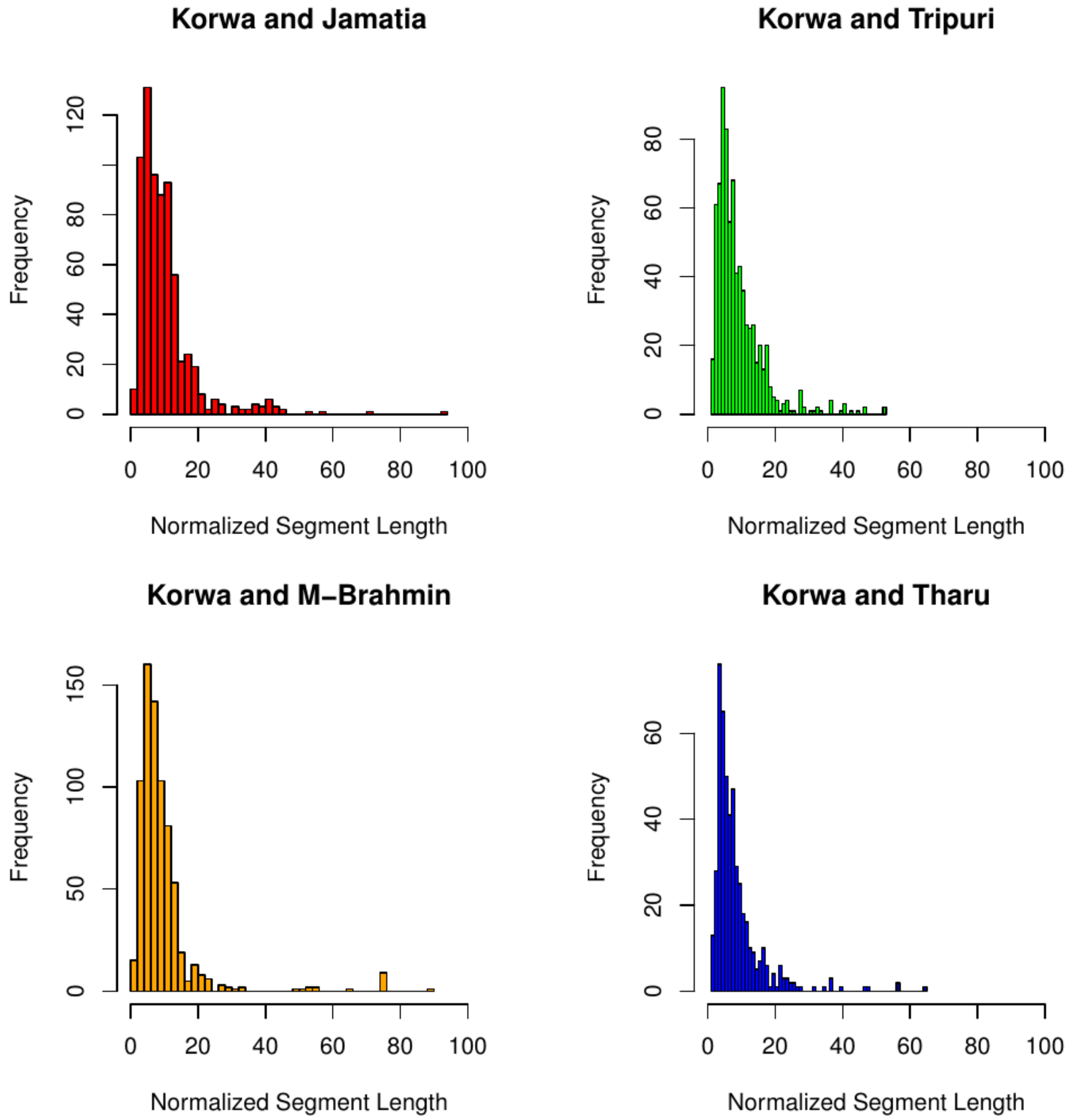

Supplementary Figure 8d: IBD estimation between TB and AAI: Estimated IBD segment length distribution between each TB subgroup and Kowa.

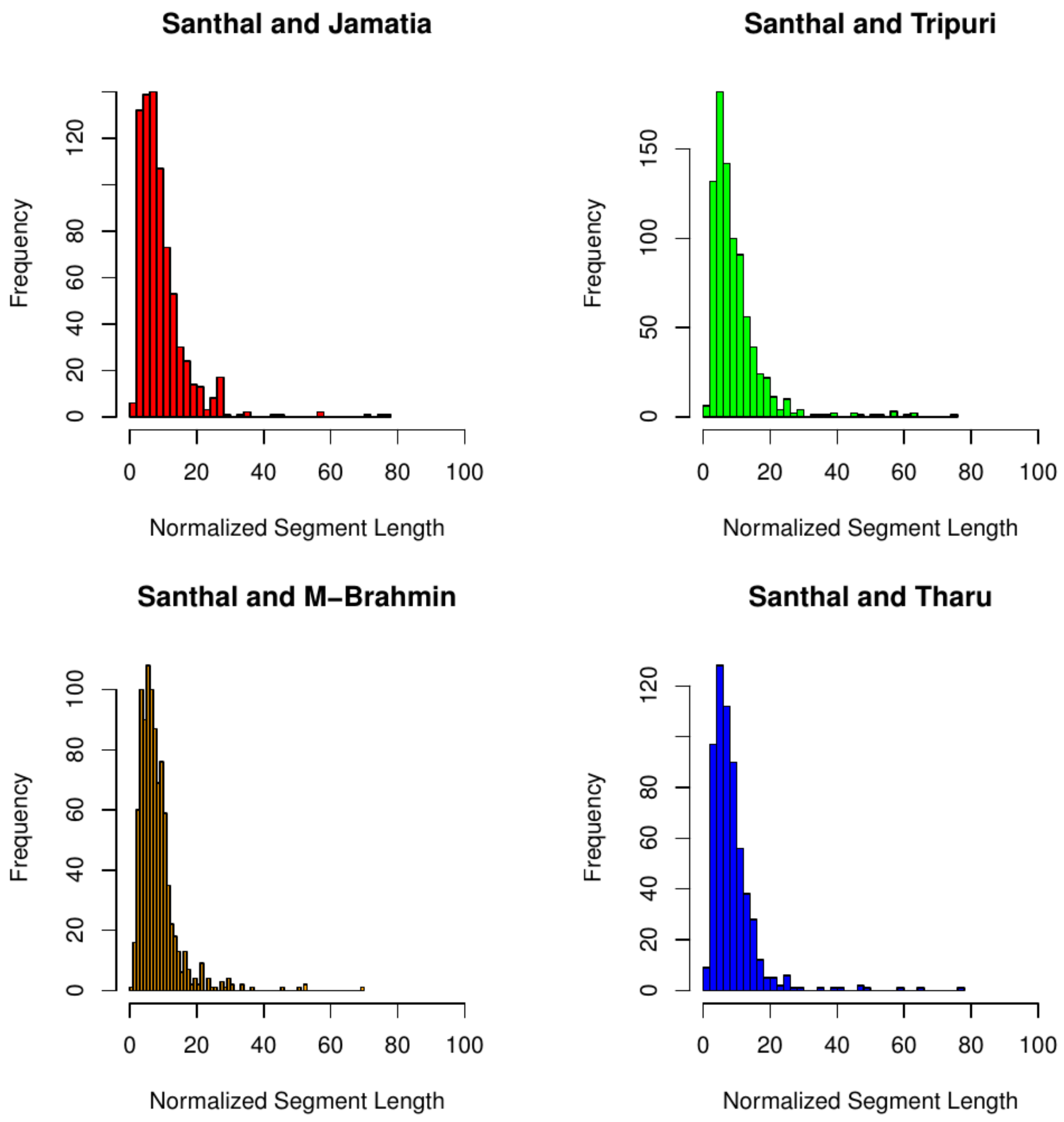

Supplementary Figure 8e: IBD estimation between TB and AAI: Estimated IBD segment length distribution between each TB subgroup and Santhal.

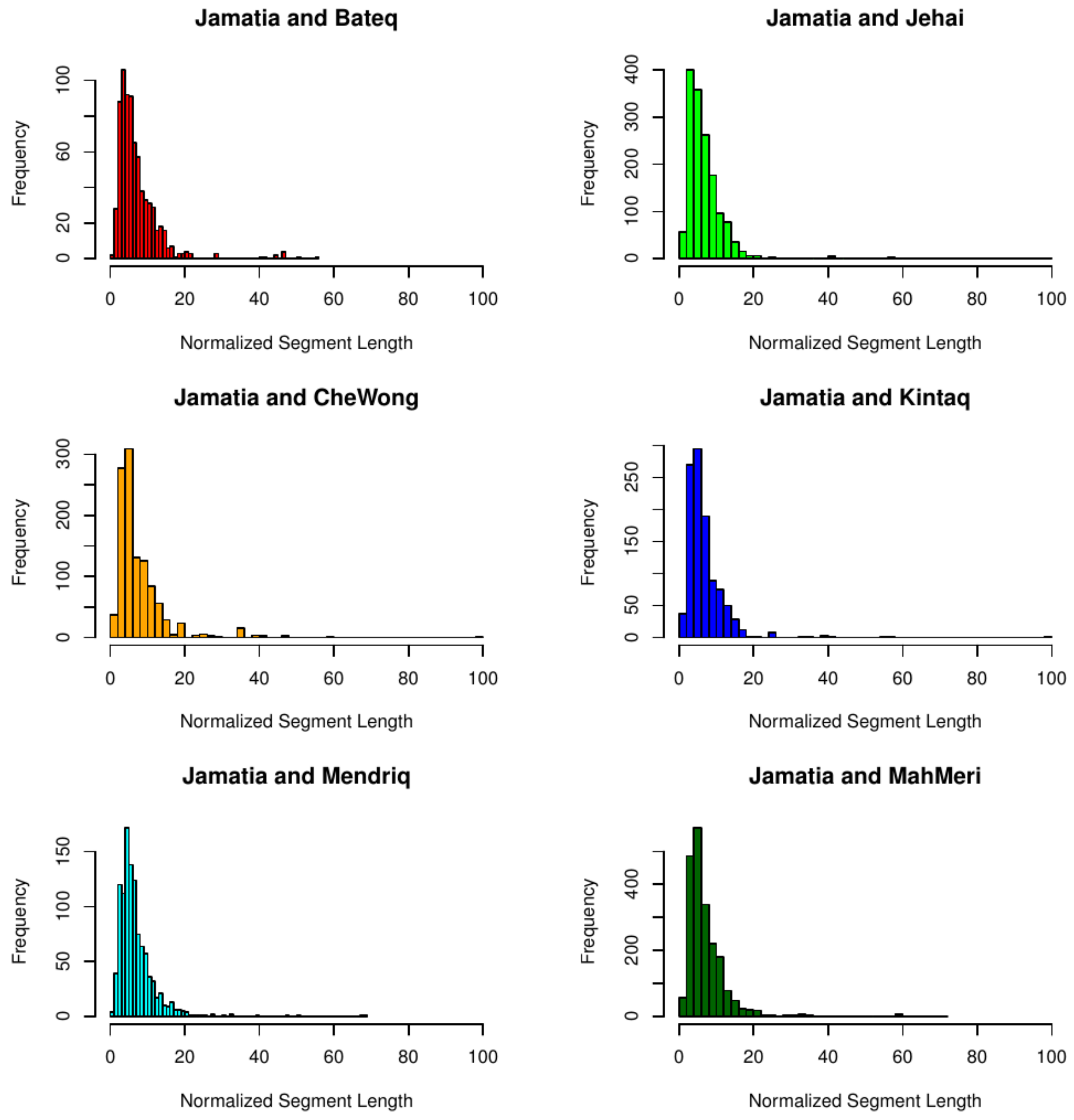

Supplementary Figure 9a: IBD estimation between AAM and TB: Estimated IBD segment length distribution between each AAM subgroup and Jamatia.

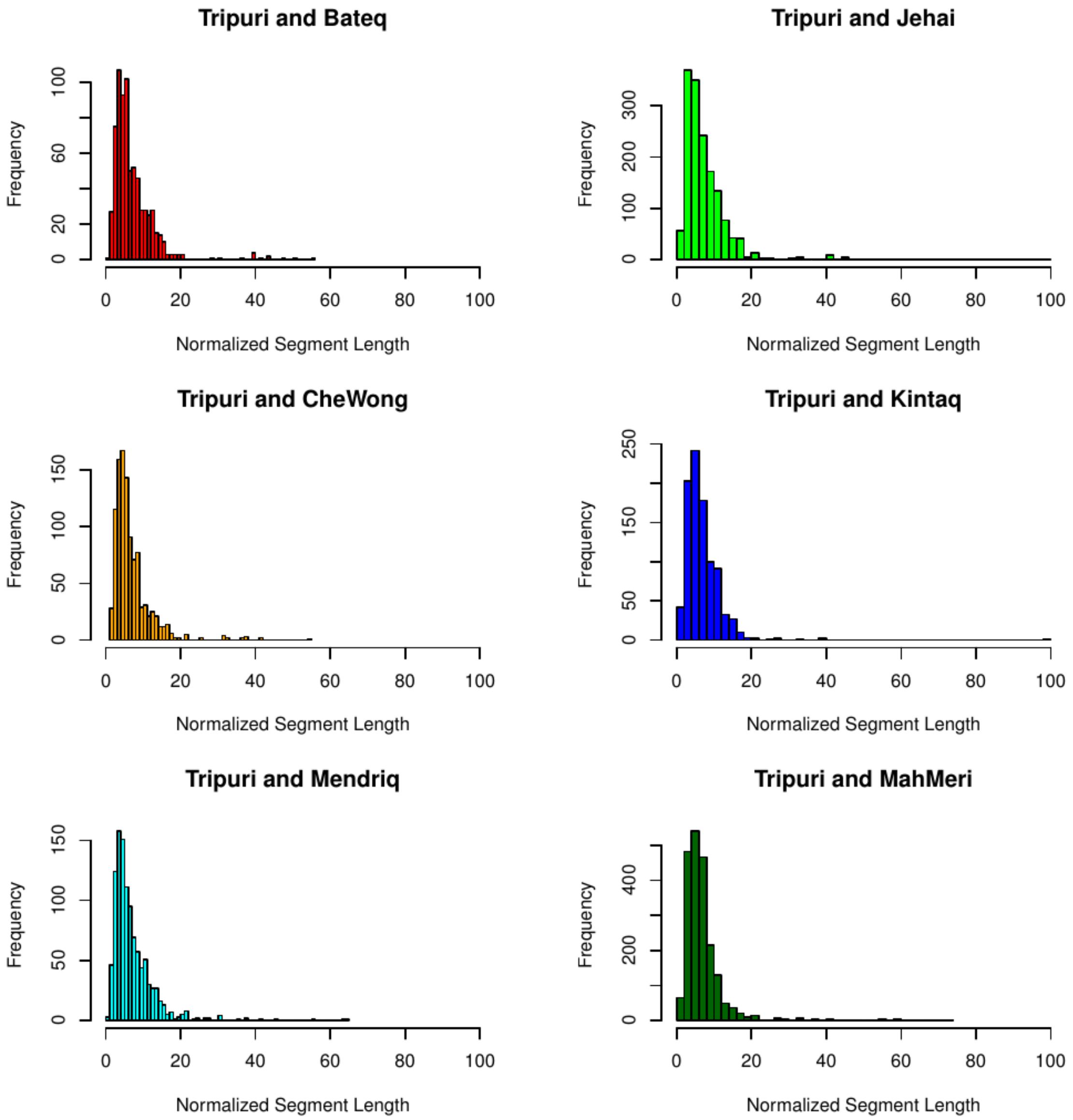

Supplementary Figure 9b: IBD estimation between AAM and TB: Estimated IBD segment length distribution between each AAM subgroup and Tripuri.

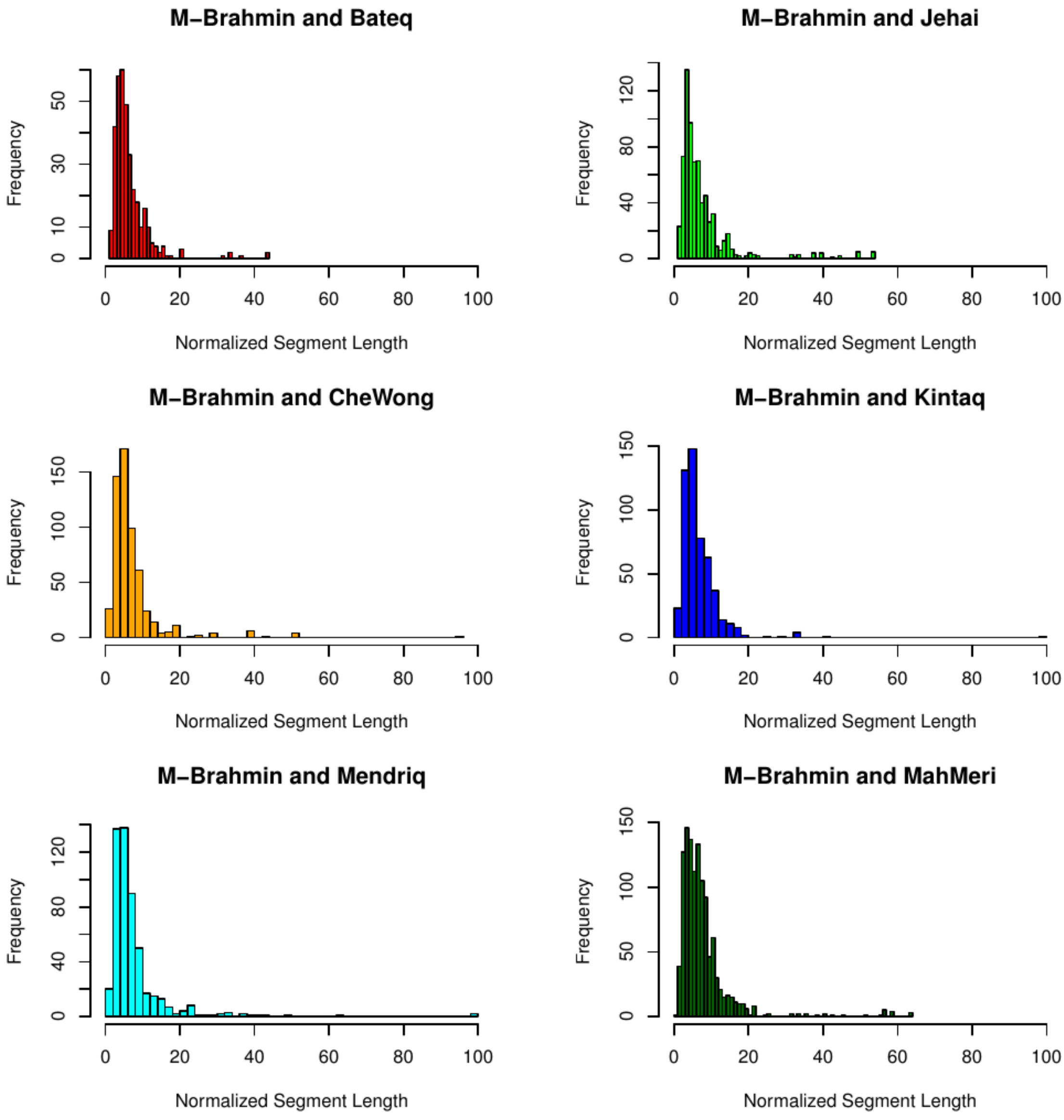

Supplementary Figure 9c: IBD estimation between AAM and TB: Estimated IBD segment length distribution between each AAM subgroup and Manipuri Brahmin.

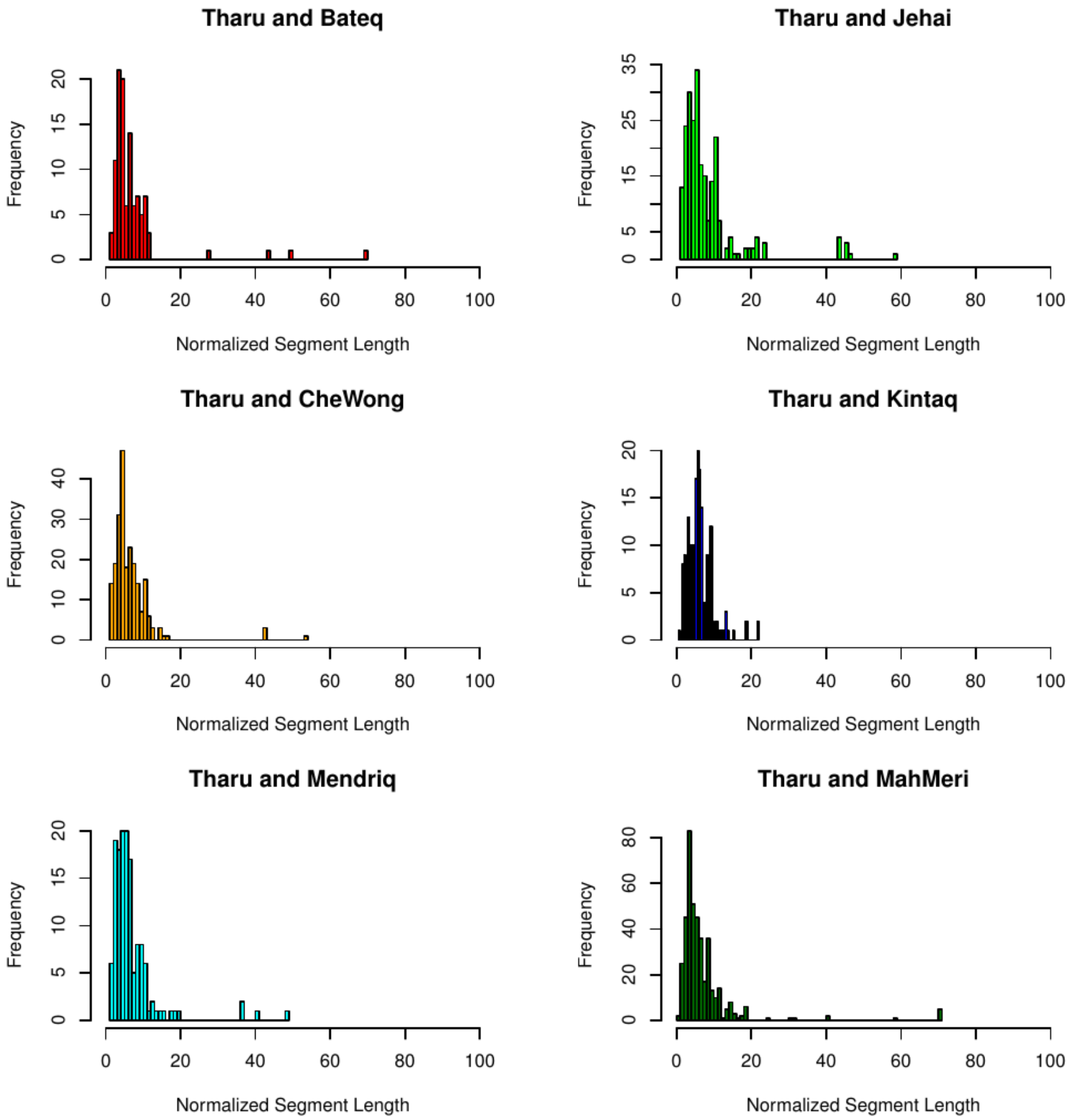

Supplementary Figure 9d: IBD estimation between AAM and TB: Estimated IBD segment length distribution between each AAM subgroup and Tharu.

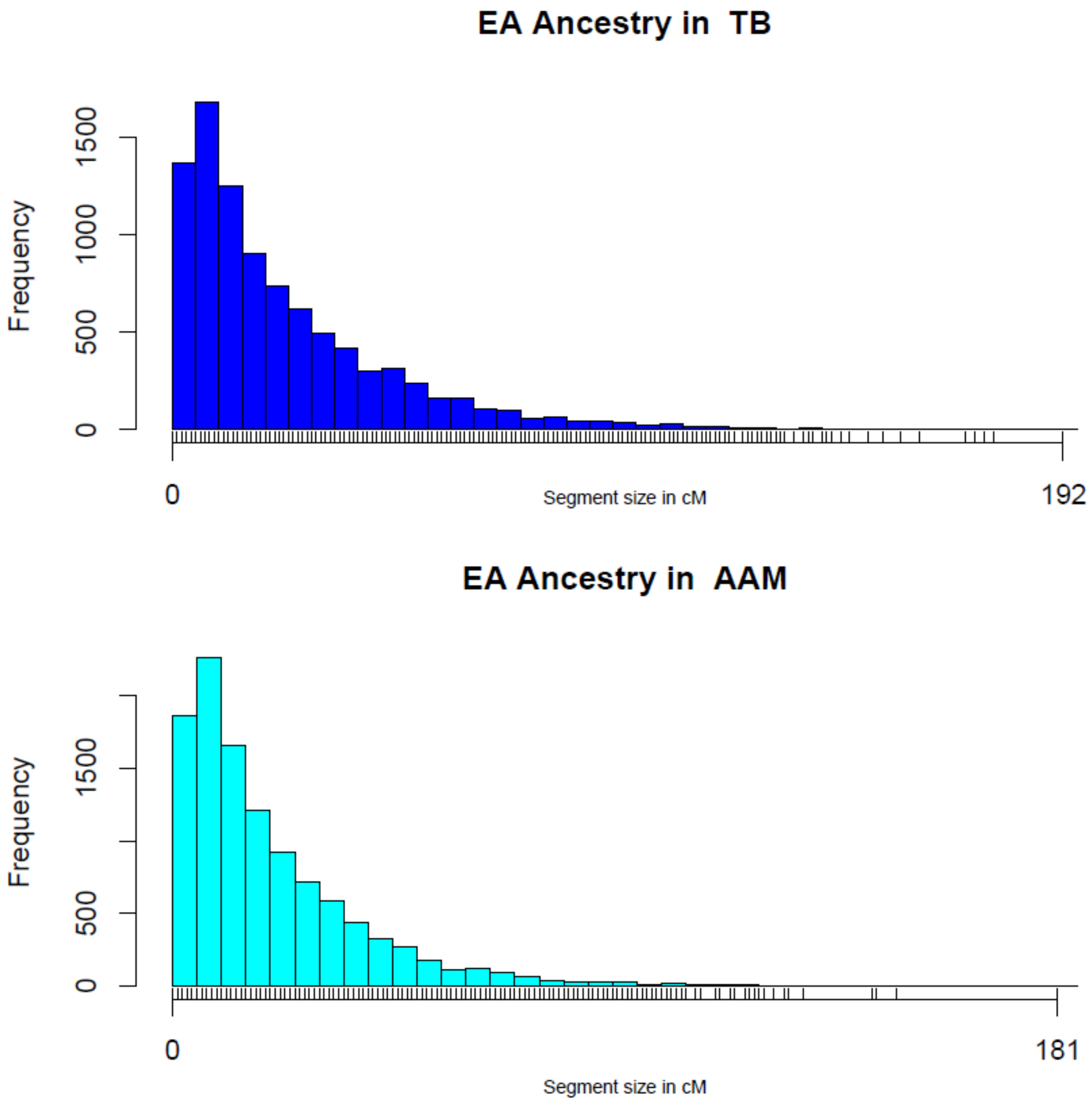

Supplementary Figure 10: Admixed segment length: Distribution of length of contiguous segment of EA ancestry in TB and AAM.

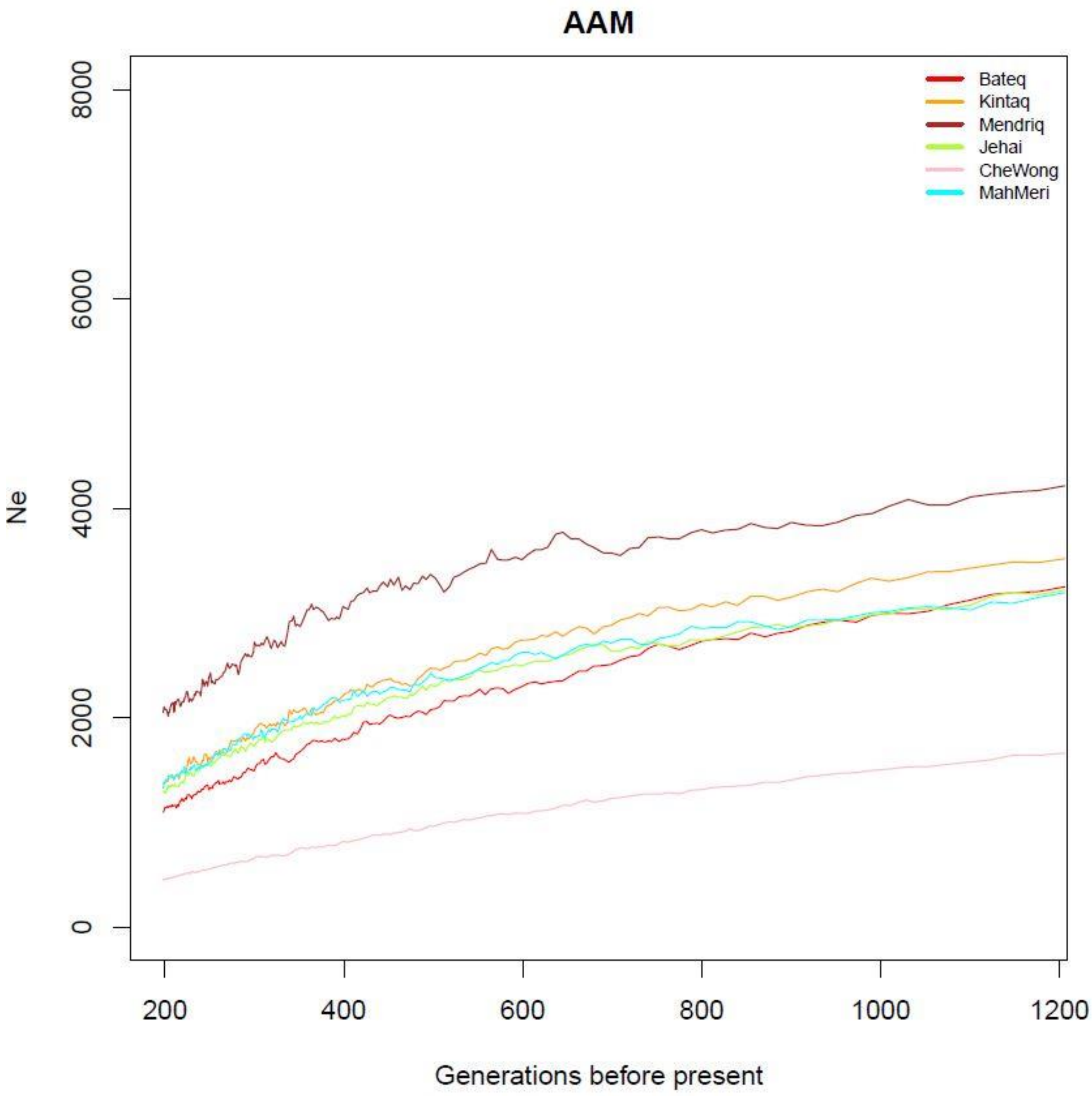

Supplementary Figure 11a: Change in effective population size: N_e was estimated for over 1000 generations in all subgroups of AAM.

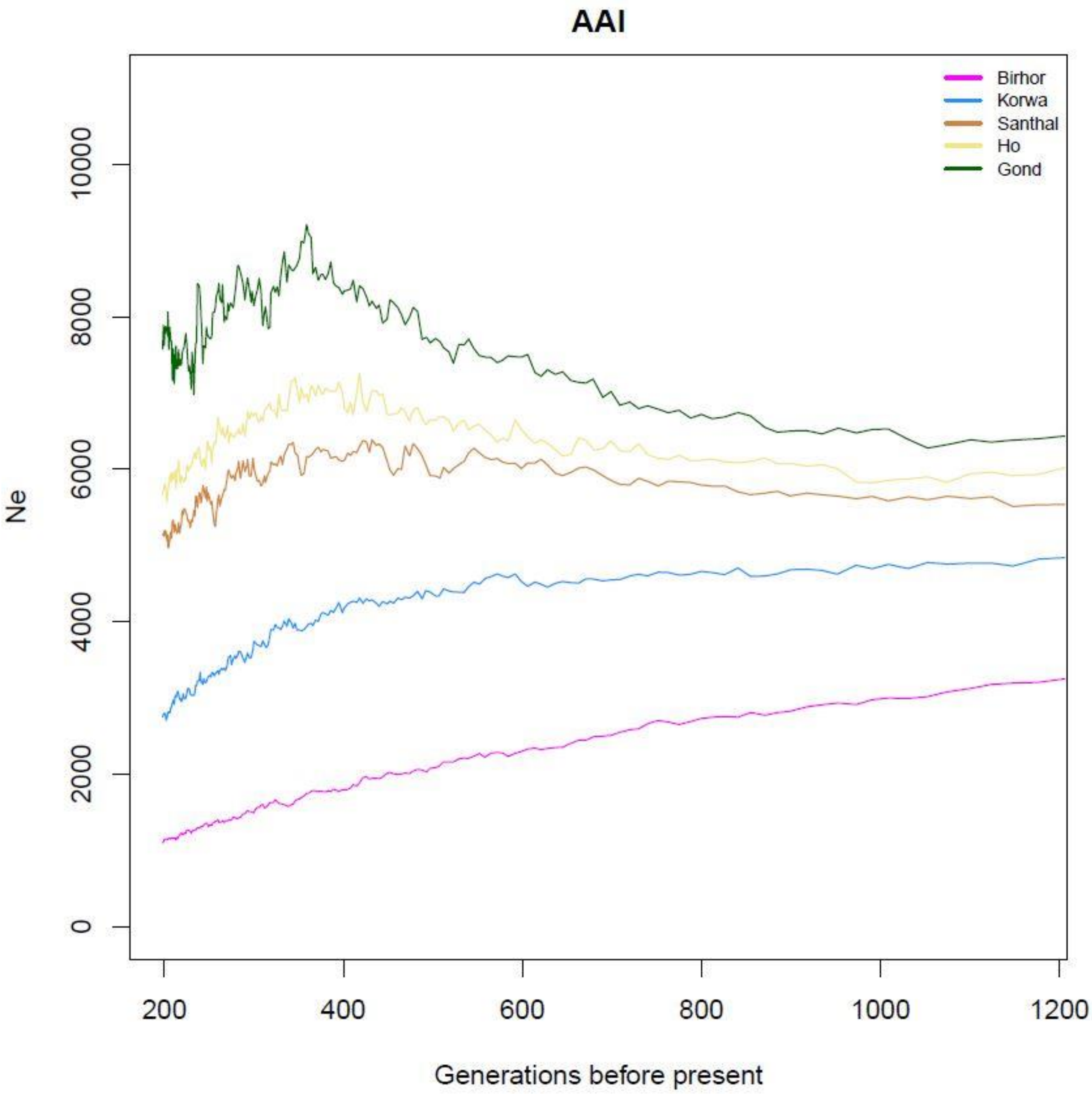

Supplementary Figure 11b: Change in effective population size: N_e was estimated for over 1000 generations in all subgroups of AAI.

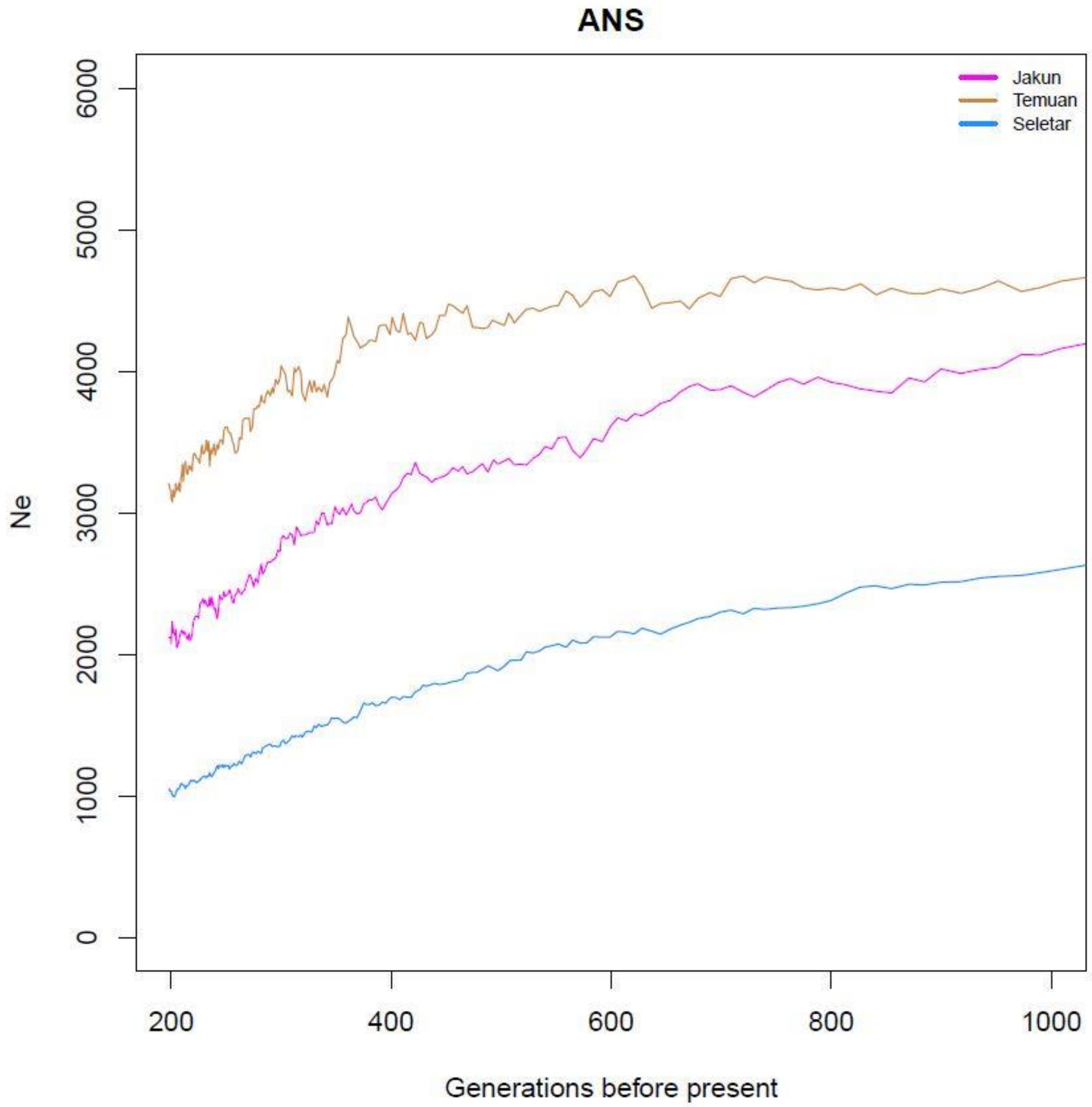

Supplementary Figure 11c: Change in effective population size: N_e was estimated for over 1000 generations in ANS.

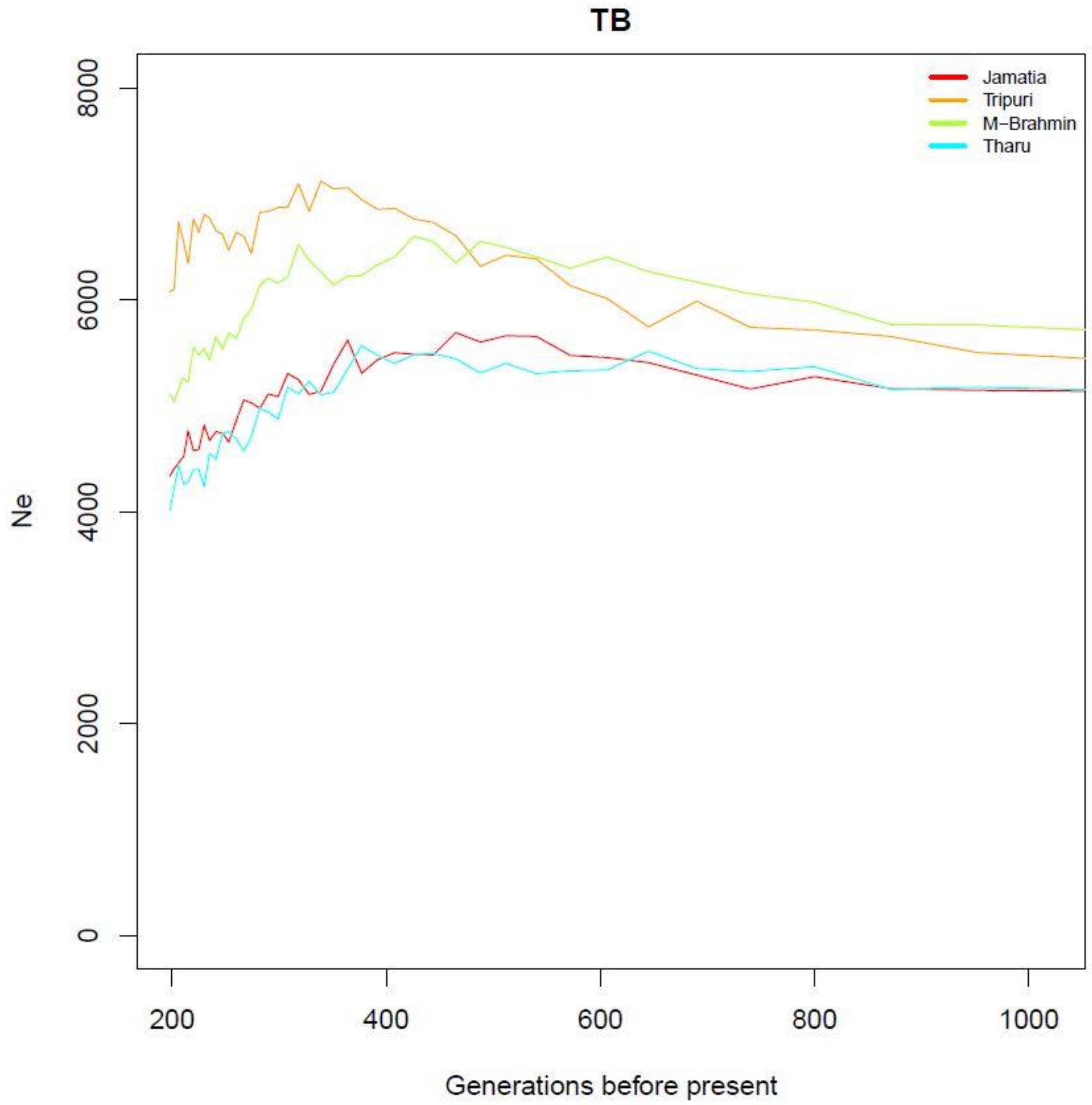

Supplementary Figure 11d: Change in effective population size: N_e was estimated for over 1000 generations in all subgroups of TB.

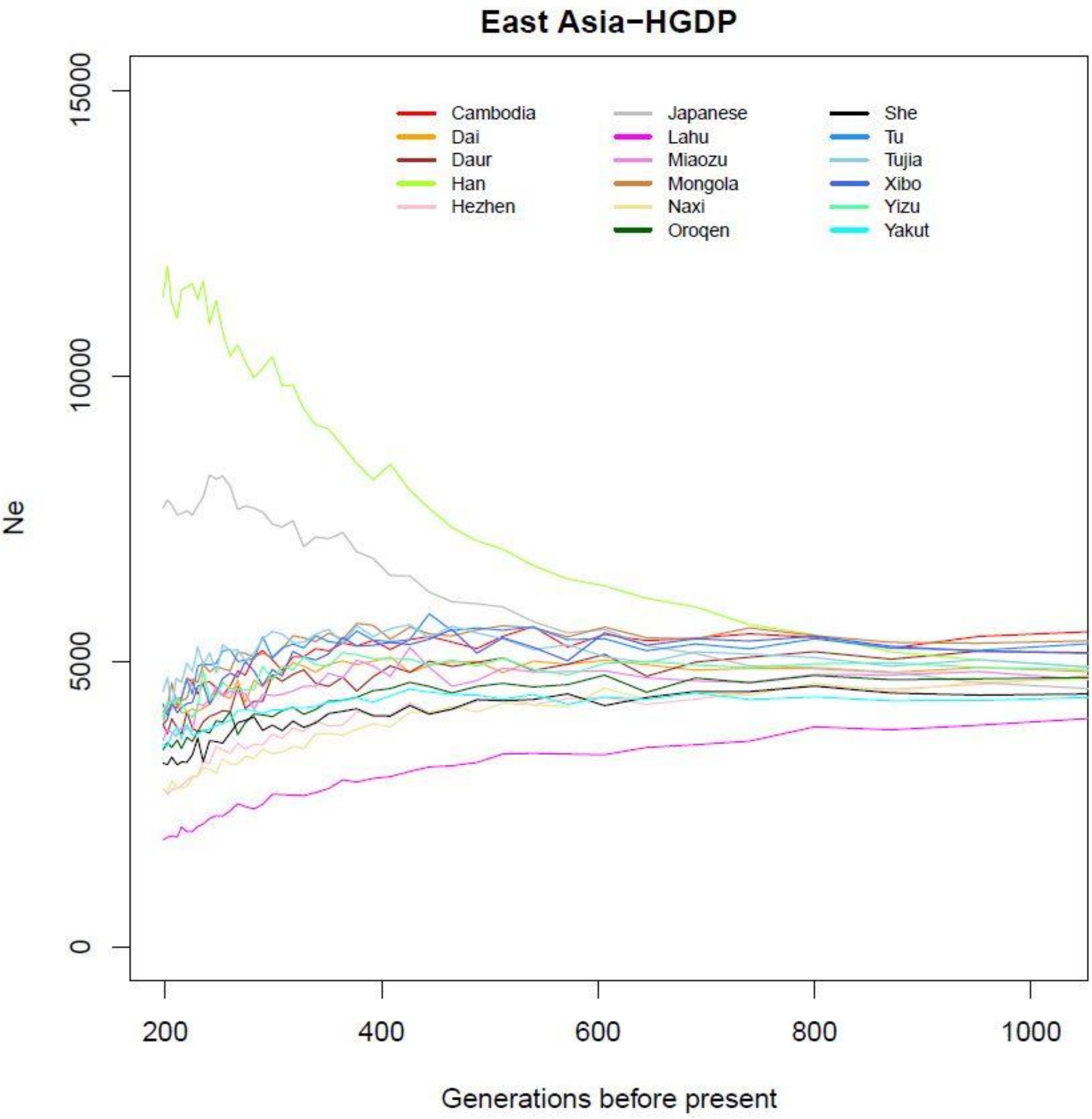

Supplementary Figure 11e: Change in effective population size: N_e was estimated for over 1000 generations in all subgroups of EA.

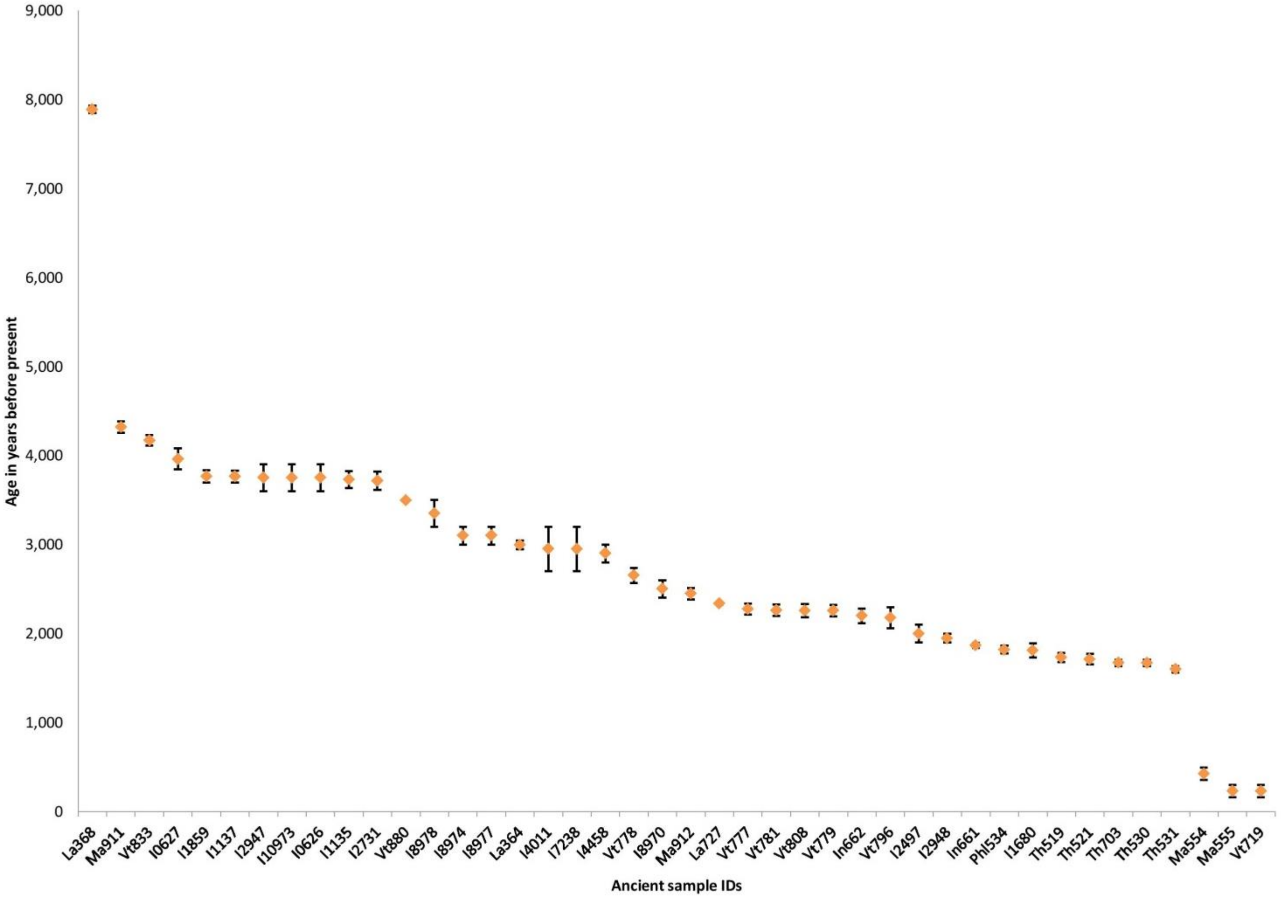

Supplementary Figure 12: The age distribution of the ancient genomes. Each dot represents an ancient genome, with the standard error of the estimate (age of one sample, La898, not available).

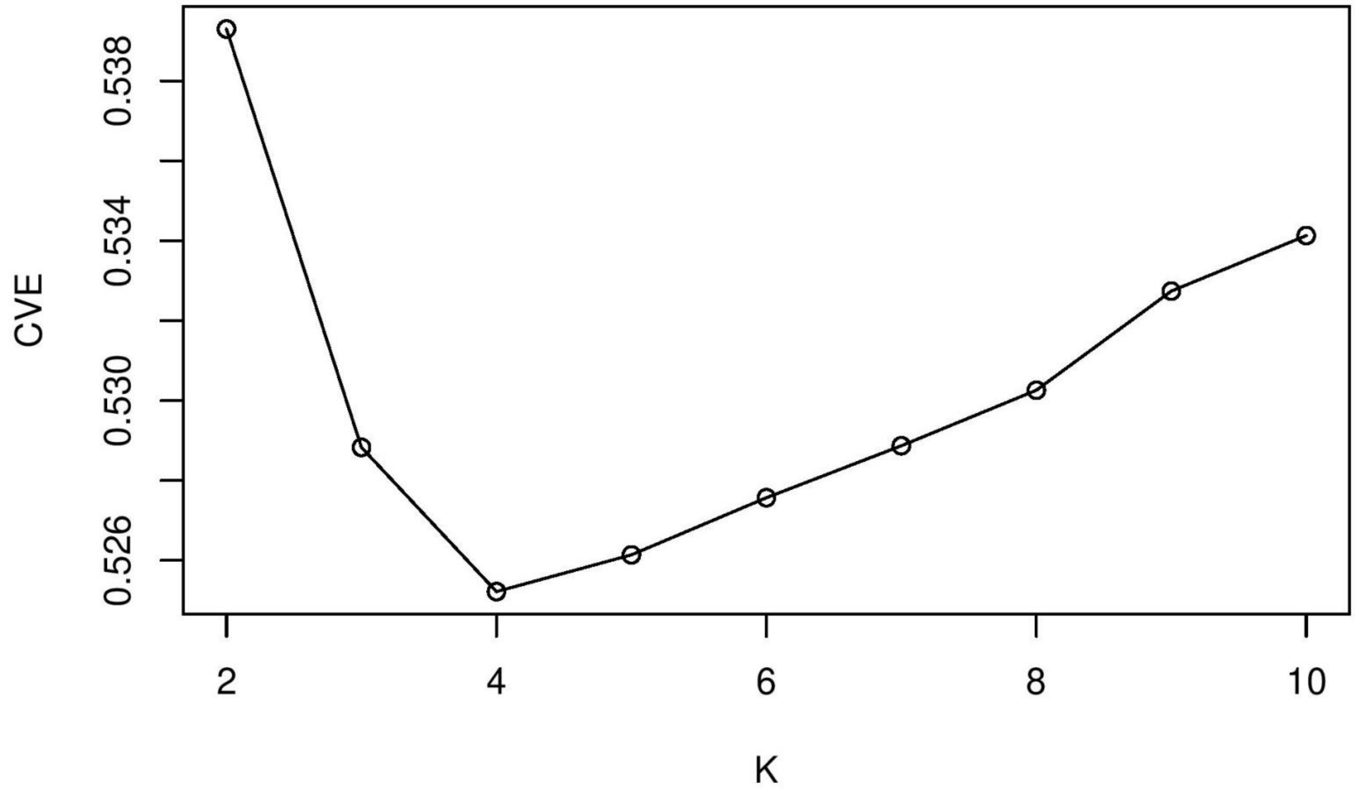

Supplementary Figure 13a: ADMIXTURE analysis on all subgroups of AAI, AAM and TB and a few subpopulations of EA: Cross validation error

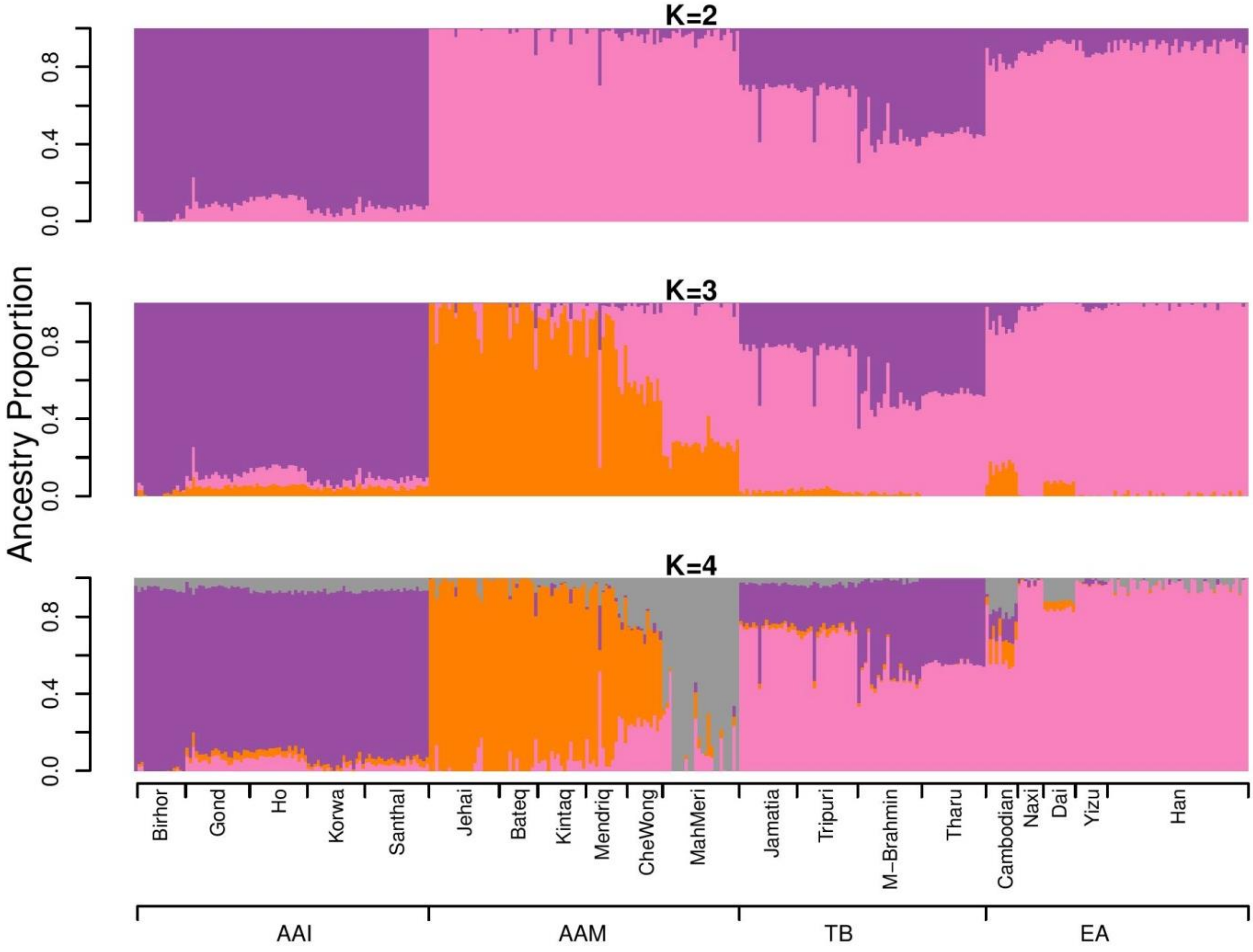

Supplementary Figure 13b: ADMIXTURE analysis on all subgroups of AAI, AAM and TB and a few subpopulations of EA: ADMIXTURE cluster graph for K=2 to K=4. The East Asian populations included in this analysis are: Han, Dai, Naxi, Yizu and Cambodian.

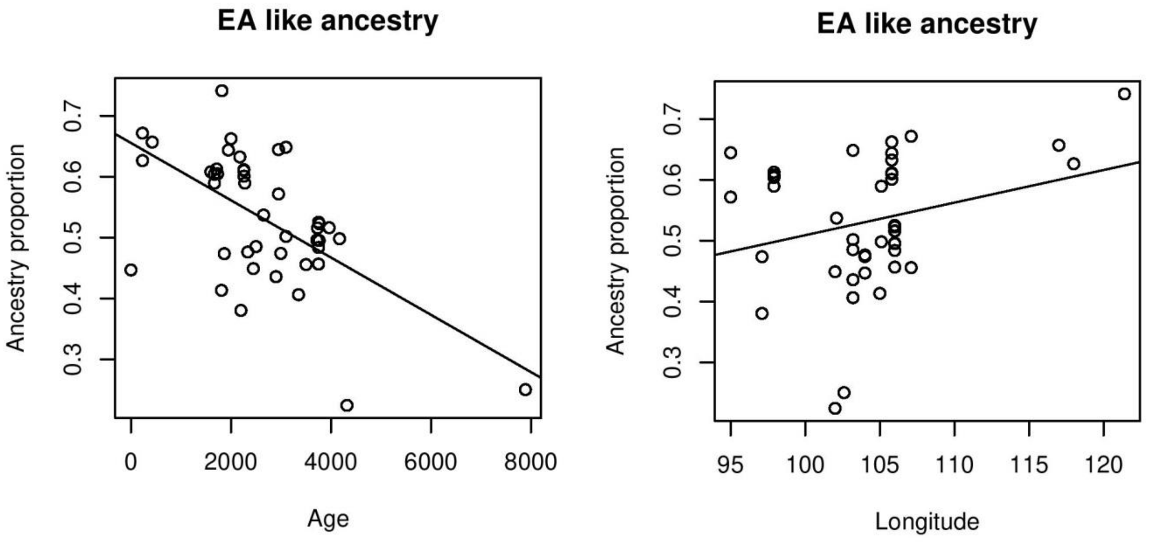

Supplementary Figure 14a: Ancestry correlation: Correlation graph of proportion of EA ancestry with age of the ancient samples (left panel) and longitudinal positions of the ancient samples (right panel)

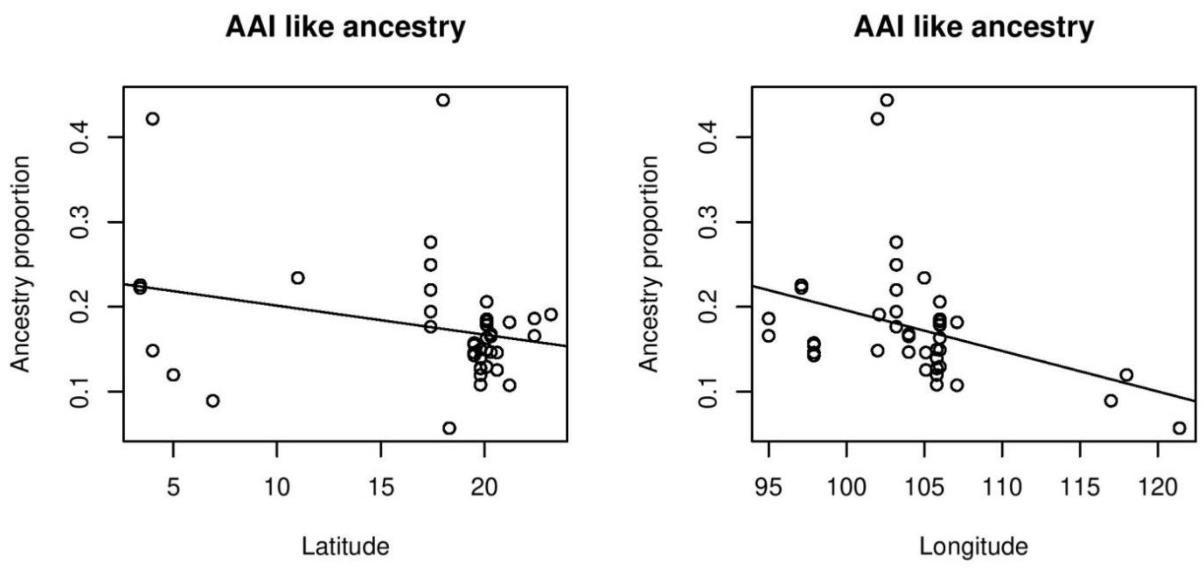

Supplementary Figure 14b: Ancestry correlation: Correlation graph of proportion of AAI ancestry with latitudinal positions of the ancient samples (left panel) and longitudinal positions of the ancient samples (right panel)

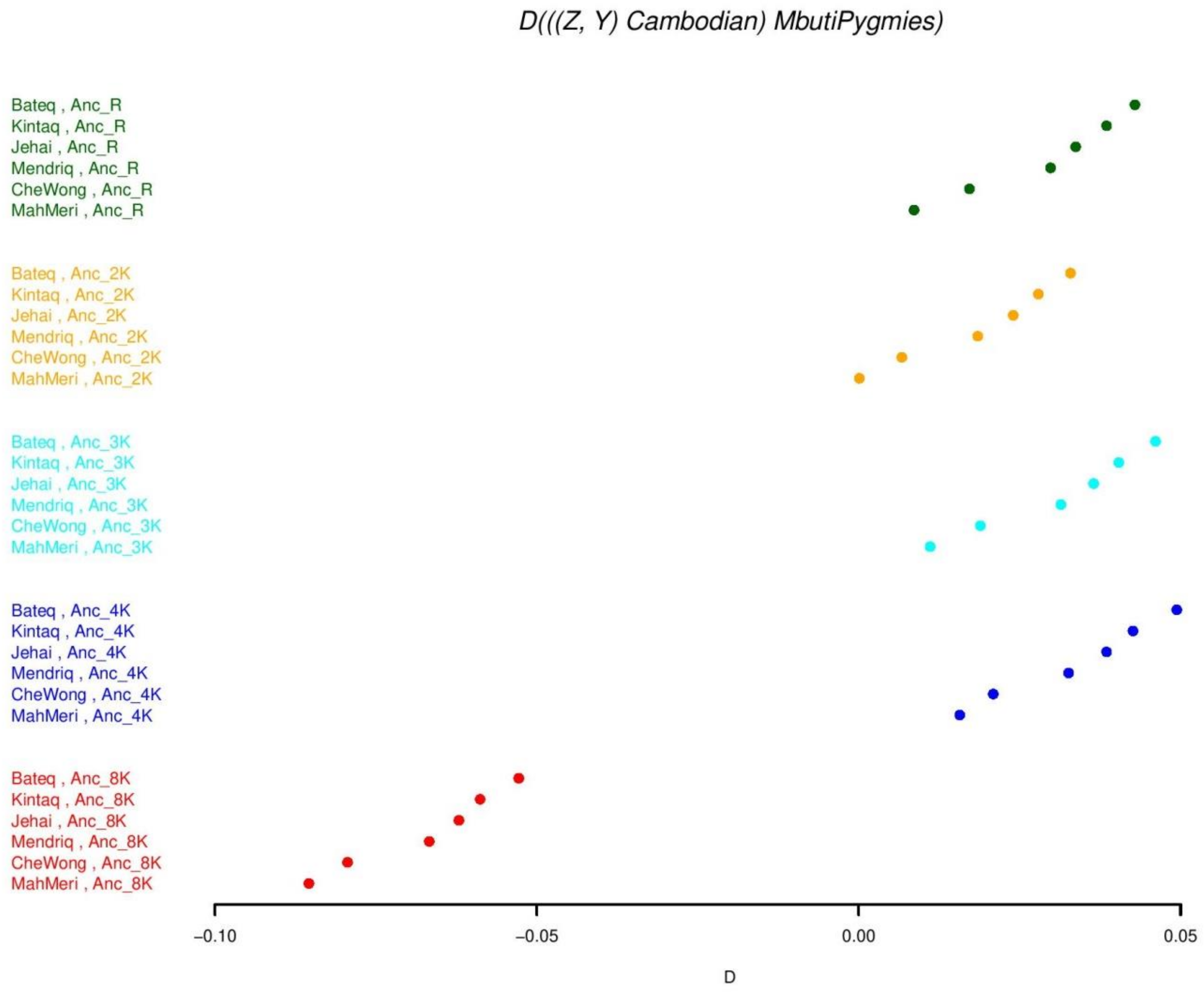

Supplementary Figure 15: D statistics value estimation: Distribution of D statistics values of the form *D(((Z,Y)Cambodian)Mbuti Pygmies)*. The x axis represents the D values. The y axis represents the pair of populations belonging to Z and Y. The y axis labels and data points are colored as dark green, orange, cyan, blue and red for Y group belonging to Anc_R, Anc_2K, Anc_3K, Anc_4K and Anc_8K respectively.
