# Supplementary material for: Insights into the demographic history of Asia from common ancestry and admixture in the genomic landscape of present-day Austroasiatic speakers
Source: BMC Biol. 2021 Mar 29;19:61. doi: 10.1186/s12915-021-00981-x (PMC8008685; doi:10.1186/s12915-021-00981-x)
Supplement: Supplementary file 2 — Additional file 2: Supplementary Table 1. Classification of populations used in the study. Supplementary Table 2. Values of outgroup f3(Mbuti Pygmy; X, Y) statistics. Supplementary Table 3. Minimum maximum and total number of bases and total number of discrete segments of IBD shared. Supplementary Table 4. Ancestry proportion estimation. Supplementary Table 5. D statistics with AAM, AAI, Southern-East Asians and an African population. Supplementary Table 6. Admixture time estimation. Supplementary Table 7. Metadata on the 43 ancient genomes. Supplementary Table 8. D statistics with Ancient genomes, AAM, Southern-East Asians and an African population. [file 12915_2021_981_MOESM2_ESM.pdf]

Table of contents in Additional file 2:

Supplementary Table 1: Classification of populations used in the study: (a) Subpopulations of the Indian dataset and their corresponding genetic ancestry, linguistic affiliation and lifestyle. **(b)** Subpopulations of the Malaysian dataset and their corresponding linguistic affiliation and lifestyle. **(c)** The different populations broadly classified, the dataset from which these populations have been obtained, the number of individuals and the abbreviations of the populations used in original publication. The original publication is referenced according to the reference in the manuscript.

Supplementary Table 2: Values of outgroup f3(Mbuti Pygmy; X,Y) statistics. **(a)** X is AAI subgroup and Y is AAM subgroup, **(b)** X is TB subgroup and Y is AAI subgroup. **(c)** X is TB subgroup and Y is AAM subgroup.

Supplementary Table 3: Minimum, maximum and total number of bases and total number of discrete segments of IBD shared. Pairwise comparison between subgroups belonging to **(a)** AAI and AAM, **(b)** AAI and TB and **(c)** AAM and TB.

Supplementary Table 4: Ancestry proportion estimation: Based on ADMIXTURE analysis on Mainland Indian, Malaysian, HGDP-Central South Asian and HGDP-East Asian populations, estimation of: **(a)** Population cluster ancestry proportion. **(b)** Individual subpopulation ancestry proportion.

Supplementary Table 5: D statistics with AAM, AAI, Southern-East Asians and an African population. Here tree topology is of the form *D(((Y,Z)W)X)* where X is Mbuti Pygmies (an African population from HGDP dataset), W is either Cambodian or Dai (Southern East Asian populations from HGDP), Y belongs to AAI subgroups (Ho, Korwa, Birhor, Santhal, Gond,) and **(a)** Z belongs to AAM subgroups (Bateq,

Kintaq,Jehai, Mendriq, CheWong, MahMeri) **(b)** Z belongs to TB subgroups (Tripuri, Jamatia, M-Brahmin, Tharu).

Supplementary Table 6: Admixture time estimation: Source population 1 and Source population 2 were representatives of respective ancestries. Using two relatively homogenous AAM populations (Jehai and MahMeri) as source for AAM ancestry, East Asian populations from different geographical locations as EA ancestry source, and different admixed population as reference, admixture between the two ancestries was estimated. Using Birhor as source for AAI ancestry, Paniya for ASI and Jamatia for ATB ancestry, admixture dates for these ancestries were estimated.

Supplementary Table 7: Metadata on the 43 ancient genomes. This includes sample ID, excavation site location and country.

Supplementary Table 8: D statistics with Ancient genomes, AAM, Southern-East Asians and an African population. Here tree topology is of the form *D(((Y,Z)W)X)* where X is Mbuti Pygmies (an African population from HGDP dataset), W is Cambodian (Southern East Asian populations from HGDP), Z belongs to AAM subgroups (Bateq, Kintaq,Jehai, Mendriq, CheWong, MahMeri) and Y belongs to Ancient genomes grouped into 5 time periods.

Supplementary Table 1a: Classification of populations used in the study: Subpopulations of the Indian dataset and their corresponding genetic ancestry genetic ancestry, linguistic affiliation and lifestyle.

Ancestral group	Population subgroups and associated abbreviations	Linguistic group	Lifestyle
Ancestral North Indian (ANI)	Kshatriya (K or KSH)	Indo European	Non hunter-gatherers
	Gujrati Brahmins (G-Brahmin)		
	West Bengal Brahmins (WB-Brahmin or W-Brahmin)		
	Marathi (MT)		
	Iyer (IR)	Dravidian	Non hunter-gatherers
	Pallan (PLN)		
Ancestral South Indian (ASI)	Kadar (KA)		Hunter-gatherers / foragers
	Irula(IL)		
	Paniya(PY)		
Ancestral Tibeto Burman (ATB)*	Manipuri Brahmin (MP or M-Brahmin)	Tibeto Burman	Non hunter-gatherers
	Jamatia (JAM)		
	Tripuri (TRI)		
	Tharu(TH)	Indo European	
Ancestral Austroasiatic (AAI)	Bihor (BIR)	Austroasiatic	Hunter-gatherers / foragers
	Gond (GD)		
	Ho (HO)		
	Korwa(KO)		
	Santhal (SA)		
Island ancestry	Jarwa(JW)	Unclassified	Hunter-gatherers / foragers
	Onge (ONG)		

*Often interchangeably used with TB

Supplementary Table 1b: Classification of populations used in the study: Subpopulations of the Malaysian dataset and their corresponding linguistic affiliation and lifestyle.

Population subgroups	Linguistic group	Lifestyle
Bateq (BTQ)	Austroasiatic (AAM)	Hunter-gatherers/foragers
CheWong (CW)		
Jehai (Jeh)		
Kintaq (Kin)		
Mendriq (Men)		
MahMeri (Mah)		
Jakun(JKN)	Austronesian (ANS)	Hunter-gatherers/foragers
Temuan(T)		
Seletar(S)		

Supplementary Table 1c: Classification of populations used in the study: The different populations broadly classified, the dataset from which these populations have been obtained, the number of individuals and the abbreviations of the populations used in original publication. The original publication is referenced according to the reference in the manuscript.

Dataset	Population(abbreviations used in this publication)	Number of individuals	Abbreviation used in original publication
HGDP	Africa	101	-
	America	64	-
	Central South Asia (CSA)	200	C.S.Asia[53]
	East Asia (EA)	228	E.Asia [53]
	Europe (EUR)	157	-
	Middle East (ME)	163	Mid.East[50]
	Oceania (OCE)	27	-
Indian population	Ancestry North India(ANI)	104	ANI[32,8]
	Ancestry South India (ASI)	58	ASI [32,8]
	Tibeto Burman (TB or ATB)	77	ATB[8]
	Indian Austroasiatics (AAI)	92	AAA[8]
	Island	36	
Malaysian population	Malaysian Austroasiatics(AAM)	97	OA[34]
	Austronesians (ANS)	47	OA[34]
	TOTAL	1451	

Supplementary Table 2a: Values of outgroup f3(Mbuti Pygmy; X,Y) statistics: X is AAI subgroup and Y is AAM subgroup.

X	Y	f3	Z
Korwa	Mendriq	0.048135	53.355
Ho	Mendriq	0.048271	54.388
Birhor	Mendriq	0.04898	52.741
Santhal	Mendriq	0.049279	54.132
Gond	Mendriq	0.054865	59.185
Korwa	Kintaq	0.06518	62.41
Ho	Kintaq	0.06557	62.67
Birhor	Kintaq	0.066312	60.87
Santhal	Kintaq	0.066522	62.57
Gond	Kintaq	0.071797	65.494
Korwa	Jehai	0.074123	64.684
Ho	Jehai	0.074368	64.165
Birhor	Jehai	0.075016	62.025
Santhal	Jehai	0.075344	65.31
Ho	MahMeri	0.077721	67.842
Korwa	MahMeri	0.077998	66.509
Birhor	MahMeri	0.078834	64.019
Santhal	MahMeri	0.07946	68.593
Gond	Jehai	0.080993	67.499
Korwa	Bateq	0.082357	56.913
Ho	Bateq	0.082929	55.984
Santhal	Bateq	0.083762	56.977
Birhor	Bateq	0.083766	55.594
Korwa	CheWong	0.085402	61.415
Ho	CheWong	0.085592	61.124
Gond	MahMeri	0.08575	72.056
Birhor	CheWong	0.086575	58.579
Santhal	CheWong	0.086847	61.47
Gond	Bateq	0.089201	58.439
Gond	CheWong	0.093084	63.54

Supplementary Table 2b: Values of outgroup f3(Mbuti Pygmy; X,Y) statistics: X is TB subgroup and Y is AAI subgroup.

X	Y	f3	Z
Tharu	Gond	0.006832	15.457
Tripuri	Gond	0.007224	14.654
Jamatia	Gond	0.007436	14.428
M-Brahmin	Gond	0.008385	20.065
Tripuri	Ho	0.008643	17.829
Jamatia	Ho	0.00881	17.371
Tharu	Ho	0.009806	22.271
M-Brahmin	Ho	0.012054	27.292
Tripuri	Santhal	0.012062	22.796
Jamatia	Santhal	0.012236	22.064
Tharu	Santhal	0.012879	26.724
M-Brahmin	Santhal	0.014669	31.914
Tripuri	Korwa	0.022141	36.235
Jamatia	Korwa	0.022264	34.771
Tharu	Korwa	0.023494	39.97
M-Brahmin	Korwa	0.025656	43.712
Tripuri	Birhor	0.03546	46.903
Jamatia	Birhor	0.035649	46.087
Tharu	Birhor	0.036733	51.691
M-Brahmin	Birhor	0.038841	54.787

Supplementary Table 2c: Values of outgroup f3(Mbuti Pygmy; X,Y) statistics: X is TB subgroup and Y is AAM subgroup.

X	Y	f3	Z
Tripuri	Mendriq	0.034082	43.757
Jamatia	Mendriq	0.034106	42.008
Tharu	Mendriq	0.044086	52.601
M-Brahmin	Mendriq	0.045805	54.552
Jamatia	Kintaq	0.052017	52.056
Tripuri	Kintaq	0.052378	54.016
Jamatia	MahMeri	0.057762	57.887
Tripuri	MahMeri	0.057954	60.098
Jamatia	Jehai	0.060464	54.448
Tripuri	Jehai	0.060599	57.458
Tharu	Kintaq	0.061807	61.034
M-Brahmin	Kintaq	0.063577	62.346
Jamatia	CheWong	0.067362	52.841
Tripuri	CheWong	0.067478	53.423
Tripuri	Bateq	0.070329	49.45
Tharu	Jehai	0.070444	63.452
Jamatia	Bateq	0.070468	48.237
Tharu	MahMeri	0.070822	65.51
M-Brahmin	Jehai	0.072516	64.856
M-Brahmin	MahMeri	0.07273	68.183
Tharu	Bateq	0.07954	54.738
Tharu	CheWong	0.079686	60.378
M-Brahmin	CheWong	0.081225	59.827
M-Brahmin	Bateq	0.08152	56.906

Supplementary Table 3a: Minimum, maximum and total number of bases and total number of discrete segments of IBD shared. Pairwise comparison between subgroups belonging to AAI and AAM.

AAI	AAM	Minimum bases shared	Maximum bases shared	Total IBD (in bp)	Total no of IBD segments
**Birhor**	Bateq	186888	9671443	427676963	361
	Jehai	214936	8594835	760837816	666
	CheWong	167207	9650568	622574439	528
	Kintaq	173477	8594835	510408810	469
	Mendriq	229683	12006064	626560819	531
	MahMeri	210054	8441474	1202914725	1032
**Gond**	Bateq	148743	7526050	253506265	214
	Jehai	154895	8313037	649390310	516
	CheWong	233975	9494118	490264419	416
	Kintaq	236600	4545037	356694776	309
	Mendriq	176670	9045142	427595901	364
	MahMeri	184416	10462011	878141024	725
**Ho**	Bateq	271131	10930100	631258257	499
	Jehai	275337	12255331	1368663376	1008
	CheWong	208935	9919086	929635744	743
	Kintaq	173477	8437517	823824222	646
	Mendriq	246911	8359693	860293482	712
	MahMeri	192037	9657407	1745051942	1385
**Korwa**	Bateq	211489	10123515	479260234	397
	Jehai	178420	9667131	1295282317	913
	CheWong	211279	10123515	845841626	684
	Kintaq	190811	8098343	766693232	602
	Mendriq	178420	12002831	895711585	656
	MahMeri	170367	9609828	1711853681	1262
**Santhal**	Bateq	220323	9087307	620368408	462
	Jehai	206806	9555360	1320482385	967
	CheWong	149468	9276198	827055081	669
	Kintaq	239803	9305290	762884003	585
	Mendriq	210436	8182402	777001444	638
	MahMeri	215974	9445944	1669677839	1276

Supplementary Table 3b: Minimum, maximum and total number of bases and total number of discrete segments of IBD shared. Pairwise comparison between subgroups belonging to AAI and TB

AAI	TB	Minimum bases shared	Maximum bases shared	Total IBD (in bp)	Total no of IBD segments
**Birhor**	Jamatia	176142	12008210	826946838	718
	Tripuri	216140	12905541	684441900	611
	Manipuri Brahmin	188090	10108173	757466071	647
	Tharu	196190	7221143	348047946	363
**Gond**	Jamatia	208136	8313037	576149358	466
	Tripuri	129929	7778964	496827650	466
	Manipuri Brahmin	158211	6913208	780644507	779
	Tharu	155017	9039725	480340001	465
**Ho**	Jamatia	144931	10104006	993465442	739
	Tripuri	202143	7314723	896697124	745
	Manipuri Brahmin	125919	10774950	910140013	793
	Tharu	147939	7341654	502521038	497
**Korwa**	Jamatia	125370	12004977	897397282	690
	Tripuri	199209	6824313	894159040	766
	Manipuri Brahmin	123185	11502604	888639734	733
	Tharu	145902	8261206	531329633	490
**Santhal**	Jamatia	127615	9861379	929439235	770
	Tripuri	148676	9653754	1024909580	842
	Manipuri Brahmin	125919	8935185	962519711	921
	Tharu	159966	9967474	669631489	599

Supplementary Table 3c: Minimum, maximum and total number of bases and total number of discrete segments of IBD shared. Pairwise comparison between subgroups belonging to AAM and TB.

TB	AAM	Minimum bases shared	Maximum bases shared	Total IBD (in bp)	Total no of IBD segments
**Jamatia**	Bateq	140799	979185	954857240	750
	Jehai	174559	17634661	1902535895	1517
	CheWong	196512	17511369	1515205093	1120
	Kintaq	203986	17634771	1296739813	1070
	Mendriq	140799	12026611	1308780187	1079
	MahMeri	200968	12585213	2609673941	2078
**Tripuri**	Bateq	175534	9808489	927813960	729
	Jehai	189007	17657734	2062013452	1539
	CheWong	197252	9545157	1249525326	1047
	Kintaq	153689	17657734	1128293711	938
	Mendriq	153689	11362768	1327176588	1070
	MahMeri	173010	12752159	2578050970	2076
**Manipuri Brahmin**	Bateq	179453	7714748	404401918	353
	Jehai	194904	9492776	962251322	707
	CheWong	162358	16810331	727780316	580
	Kintaq	211333	17399030	618052665	523
	Mendriq	176799	17415393	699955800	518
	MahMeri	137535	11133244	1600936742	1170
**Tharu**	Bateq	269410	12306005	134179941	107
	Jehai	276828	10375954	355627466	238
	CheWong	195453	9381729	262493829	225
	Kintaq	173477	3815490	201352253	185
	Mendriq	272455	8528859	170707865	518
	MahMeri	141375	12381331	525534963	414

Supplementary Table 4a: Ancestry proportion estimation: Based on ADMIXTURE analysis on Mainland Indian, Malaysian, HGDP-Central South Asian and HGDP-East Asian populations, estimation of population cluster ancestry proportion.

	Southern EA-major	CheWong-major	MahMeri-major	AAM-major	Seletar-major	Northern EA-major	AAI-major	CSA-major	ASI-major
**ANI**	0.21	0.22	0.39	0.36	0.33	0.97	21.55	54.70	21.26
**ASI**	0.08	0.17	0.22	0.25	0.14	0.10	7.51	5.50	86.04
**AAI**	2.77	1.50	1.98	0.97	1.07	0.41	69.99	4.57	16.74
**ATB**	52.98	1.62	1.69	1.04	0.35	5.64	15.33	11.85	9.50
**AAM**	5.41	11.54	23.99	57.30	0.93	0.08	0.37	0.17	0.21
**ANS**	23.63	8.37	14.22	6.31	44.48	0.04	2.77	0.12	0.07
**CSA**	5.47	0.87	0.82	0.86	0.54	5.96	4.06	76.58	4.83
**EA**	68.09	1.32	2.18	0.44	0.85	25.27	0.44	1.33	0.07

Supplementary Table 4b: Ancestry proportion estimation: Based on ADMIXTURE analysis on Mainland Indian, Malaysian, HGDP-Central South Asian and HGDP-East Asian populations, estimation of individual subpopulation ancestry proportion.

	Southern EA-major	CheWong-major	MahMeri-major	AAM-major	Seletar-major	Northern EA-major	AAI-major	CSA-major	ASI-major
Kshatriya	0.45	0.52	0.74	0.49	0.42	2.56	9.77	75.14	9.91
G-Brahmin	0.28	0.11	0.48	0.4	0.65	1.56	15.31	65.7	15.49
W-Brahmin	1.57	0.45	0.64	0.66	0.52	1.77	20.6	56.22	17.59
Marathi	0.05	0.11	0.01	0.38	0.07	0.39	30.12	39.86	29.01
Iyer	0.23	0.33	0.6	0.22	0.23	0.22	22.23	56.32	19.63
Pallan	0.01	0.04	0.03	0.32	0.22	0.01	32.31	33.05	34.02
Paniya	0.01	0.12	0.11	0.1	0.13	0.08	0.77	2.48	96.2
Irula	0.04	0.16	0.29	0.06	0.11	0.08	3.9	5.59	89.77
Kadar	0.18	0.22	0.23	0.58	0.19	0.12	17.18	8.12	73.18
Birhor	0.48	0.3	0.33	0.13	0.48	0.19	96.64	0.49	0.98
Gond	2.16	1.79	1.93	1.09	0.8	0.5	47.23	13.73	30.76
Ho	5.91	2.73	3.14	1.46	1.52	0.23	61.26	2.27	21.49
Korwa	2.39	0.84	2.07	0.95	1.07	0.51	82.62	1.25	8.31
Santhal	2.73	1.67	2.22	1.08	1.39	0.58	67.93	3.76	18.65
Tripuri	61.55	2.71	3.1	2.01	0.44	5.34	12.21	6.19	6.44
Jamatia	64.43	2.65	2.78	1.67	0.53	3.55	12.43	5.88	6.08
M-Brahmin	41.42	1.27	1.07	0.47	0.39	5.49	15.84	23.09	10.95
Tharu	46.1	0	0	0.12	0.05	7.95	20.41	11.34	14.04
Kintaq	3.28	4.32	2.82	87.51	0.7	0.3	0.42	0.18	0.46
Jehai	1.85	1.06	1.62	94.86	0.44	0	0.07	0	0.11
Bateq	2.39	1.14	1.46	93.2	0.46	0.06	0.63	0.32	0.34
Mendriq	11.02	7.16	6.88	69.78	2.47	0.2	1.31	0.78	0.4
CheWong	6.03	79.6	5.07	7.95	1.32	0	0.03	0	0
Mahmeri	8.2	2.06	86.91	1.89	0.76	0	0.12	0	0.07
Temuan	34.79	13.1	31.52	10.45	5.57	0	4.21	0.23	0.13
Jakun	33.02	18.48	20.56	13.82	7.27	0	6.51	0.22	0.12
Seletar	12.82	0.85	1.42	0.37	84.27	0.08	0.18	0	0.01
Balochi	0.87	1.28	0.73	1.14	0.54	1.02	3.16	86.34	4.91
Brahui	0.84	1.2	1.02	1.22	0.53	0.59	1.51	88.56	4.53
Burusho	0.87	1.28	0.73	1.14	0.54	1.02	3.16	86.34	4.91
Uyugur	27.42	0.3	0.91	0.69	0.21	23.71	0.06	46.04	0.66
Hazara	22.23	0.87	0.55	0.35	0.52	27.71	0.35	46.94	0.49
Kalash	0.9	1.34	0.76	1.19	0.56	1.07	3.3	90.1	5.13
Makrani	0.48	1.47	0.9	1.33	0.86	0.71	1.78	88.74	3.73
Pathan	0.65	0.64	0.79	0.77	0.48	3.8	7.58	77.4	7.89
Sindhi	0.31	0.64	0.68	0.75	0.78	1.25	10.51	74.18	10.9
Cambodian	56.18	9.33	13.79	5.01	5.32	0	7.69	2.67	0.01
Dai	80.12	6.02	8.88	1.59	3.31	0	0.07	0	0
Han	56.18	9.33	13.79	5.01	5.32	0	7.69	2.67	0.01

**Miaozu**	92.3	1.61	3.41	0.16	1.3	1.22	0	0	0
**She**	95.91	0.92	2.63	0.03	0.43	0.07	0	0	0
**Yizu**	56.18	9.33	13.79	5.01	5.32	0	7.69	2.67	0.01
**Lahu**	81.41	5.99	8.1	1.15	2.52	0.11	0.65	0	0.07
**Tujia**	91.94	0.76	1.94	0.24	0.43	4.68	0	0	0
**Japanese**	77.93	0.06	0.07	0.09	0.21	21.65	0	0	0
**Naxi**	83.84	0.28	1.14	0.65	0.29	12.37	0.46	0	0.97
**Tu**	73.05	0.19	0.09	0.05	0.32	19.59	0.29	6.13	0.29
**Xibo**	61.51	0.22	0.07	0.03	0.14	32.86	0	5	0.17
**Mongola**	56.71	0.18	0.22	0.27	0.1	38.17	0	4.32	0.03
**Oroqen**	40.7	0	0	0	0	59.29	0	0	0
**Daur**	51.76	0	0	0	0.11	47.88	0	0.24	0
**Hezhen**	52.13	0	0.01	0	0	47.75	0	0.11	0
**Yakut**	0.19	0.34	0.19	0.11	0.14	94.38	0.04	4.54	0.06

Supplementary Table 5a: D statistics with AAM, AAI, Southern-East Asians and an African population. Here tree topology is of the form *D(((Y,Z)W)X)* where X is Mbuti Pygmies (an African population from HGDP dataset), W is either Cambodian or Dai (Southern East Asian populations from HGDP), Y belongs to AAI subgroups (Ho, Korwa, Birhor, Santhal, Gond,) and Z belongs to AAM subgroups (Bateq, Kintaq,Jehai, Mendriq, CheWong, MahMeri).

W	X	Y	Z	D	Z-score
Cambodian	Mbuti Pygmies	Ho	Bateq	-0.031	-17.521
Cambodian	Mbuti Pygmies	Korwa	Bateq	-0.0311	-17.725
Cambodian	Mbuti Pygmies	Birhor	Bateq	-0.0336	-17.729
Cambodian	Mbuti Pygmies	Santhal	Bateq	-0.0341	-19.185
Cambodian	Mbuti Pygmies	Ho	Kintaq	-0.0362	-22.386
Cambodian	Mbuti Pygmies	Korwa	Kintaq	-0.0363	-22.425
Cambodian	Mbuti Pygmies	Birhor	Kintaq	-0.0388	-22.055
Cambodian	Mbuti Pygmies	Santhal	Kintaq	-0.0393	-24.319
Cambodian	Mbuti Pygmies	Ho	Jehai	-0.04	-23.766
Cambodian	Mbuti Pygmies	Korwa	Jehai	-0.0402	-23.705
Cambodian	Mbuti Pygmies	Birhor	Jehai	-0.0427	-23.705
Cambodian	Mbuti Pygmies	Santhal	Jehai	-0.0431	-25.424
Cambodian	Mbuti Pygmies	Ho	Mendriq	-0.0452	-29.351
Cambodian	Mbuti Pygmies	Korwa	Mendriq	-0.0453	-29.445
Cambodian	Mbuti Pygmies	Gond	Bateq	-0.0468	-26.189
Cambodian	Mbuti Pygmies	Birhor	Mendriq	-0.0478	-28.685
Cambodian	Mbuti Pygmies	Santhal	Mendriq	-0.0482	-30.87
Cambodian	Mbuti Pygmies	Gond	Kintaq	-0.052	-32.231
Cambodian	Mbuti Pygmies	Gond	Jehai	-0.0557	-32.933
Cambodian	Mbuti Pygmies	Ho	CheWong	-0.0574	-34.141
Cambodian	Mbuti Pygmies	Korwa	CheWong	-0.0575	-32.906
Cambodian	Mbuti Pygmies	Birhor	CheWong	-0.06	-31.989
Cambodian	Mbuti Pygmies	Santhal	CheWong	-0.0604	-34.018
Cambodian	Mbuti Pygmies	Gond	Mendriq	-0.0608	-39.238
Cambodian	Mbuti Pygmies	Korwa	MahMeri	-0.0648	-39.439
Cambodian	Mbuti Pygmies	Ho	MahMeri	-0.0648	-40.279
Cambodian	Mbuti Pygmies	Birhor	MahMeri	-0.0673	-36.894
Cambodian	Mbuti Pygmies	Santhal	MahMeri	-0.0677	-40.895
Cambodian	Mbuti Pygmies	Gond	CheWong	-0.0729	-41.76
Cambodian	Mbuti Pygmies	Gond	MahMeri	-0.08	-47.86
Dai	Mbuti Pygmies	Ho	Bateq	-0.0329	-17.829
Dai	Mbuti Pygmies	Korwa	Bateq	-0.0339	-18.654
Dai	Mbuti Pygmies	Birhor	Bateq	-0.0358	-18.41
Dai	Mbuti Pygmies	Santhal	Bateq	-0.0365	-19.867
Dai	Mbuti Pygmies	Ho	Kintaq	-0.0381	-22.527
Dai	Mbuti Pygmies	Korwa	Kintaq	-0.0391	-23.098
Dai	Mbuti Pygmies	Birhor	Kintaq	-0.041	-22.297
Dai	Mbuti Pygmies	Santhal	Kintaq	-0.0417	-24.523
Dai	Mbuti Pygmies	Ho	Jehai	-0.042	-23.616
Dai	Mbuti Pygmies	Korwa	Jehai	-0.043	-24.111
Dai	Mbuti Pygmies	Birhor	Jehai	-0.0449	-23.72
Dai	Mbuti Pygmies	Santhal	Jehai	-0.0456	-25.389
Dai	Mbuti Pygmies	Ho	Mendriq	-0.048	-29.449

Dai	Mbuti Pygmies	Korwa	Mendriq	-0.049	-29.692
Dai	Mbuti Pygmies	Gond	Bateq	-0.0501	-26.902
Dai	Mbuti Pygmies	Birhor	Mendriq	-0.0509	-28.875
Dai	Mbuti Pygmies	Santhal	Mendriq	-0.0515	-31.082
Dai	Mbuti Pygmies	Gond	Kintaq	-0.0552	-31.929
Dai	Mbuti Pygmies	Gond	Jehai	-0.0591	-32.621
Dai	Mbuti Pygmies	Ho	CheWong	-0.0607	-34.002
Dai	Mbuti Pygmies	Korwa	CheWong	-0.0616	-33.583
Dai	Mbuti Pygmies	Birhor	CheWong	-0.0635	-32.763
Dai	Mbuti Pygmies	Santhal	CheWong	-0.0642	-34.639
Dai	Mbuti Pygmies	Gond	Mendriq	-0.065	-38.873
Dai	Mbuti Pygmies	Ho	MahMeri	-0.0702	-41.615
Dai	Mbuti Pygmies	Korwa	MahMeri	-0.0711	-41.685
Dai	Mbuti Pygmies	Birhor	MahMeri	-0.073	-39.481
Dai	Mbuti Pygmies	Santhal	MahMeri	-0.0737	-43.239
Dai	Mbuti Pygmies	Gond	CheWong	-0.0775	-42.132
Dai	Mbuti Pygmies	Gond	MahMeri	-0.0868	-51.386

Supplementary Table 5b: D statistics with TB, AAI, Southern-East Asians and an African population. Here tree topology is of the form *D(((Y,Z)W)X)* where X is Mbuti Pygmies (an African population from HGDP dataset), W is either Cambodian or Dai (Southern East Asian populations from HGDP), Y belongs to AAI subgroups (Ho, Korwa, Birhor, Santhal, Gond,) and Z belongs to TB subgroups (Tripuri, Jamatia, M-Brahmin, Tharu).

W	X	Y	Z	D	Z-score
Cambodian	Mbuti Pygmies	Ho	M-Brahmin	-0.0153	-14.081
Cambodian	Mbuti Pygmies	Korwa	M-Brahmin	-0.0155	-12.685
Cambodian	Mbuti Pygmies	Birhor	M-Brahmin	-0.018	-13.44
Cambodian	Mbuti Pygmies	Santhal	M-Brahmin	-0.0185	-16.256
Cambodian	Mbuti Pygmies	Ho	Tharu	-0.0208	-17.997
Cambodian	Mbuti Pygmies	Korwa	Tharu	-0.0209	-16.528
Cambodian	Mbuti Pygmies	Birhor	Tharu	-0.0235	-16.473
Cambodian	Mbuti Pygmies	Santhal	Tharu	-0.0239	-19.678
Cambodian	Mbuti Pygmies	Gond	M-Brahmin	-0.0314	-28.69
Cambodian	Mbuti Pygmies	Gond	Tharu	-0.0369	-31.375
Cambodian	Mbuti Pygmies	Ho	Tripuri	-0.0467	-36.105
Cambodian	Mbuti Pygmies	Korwa	Tripuri	-0.0468	-35.618
Cambodian	Mbuti Pygmies	Ho	Jamatia	-0.0474	-33.892
Cambodian	Mbuti Pygmies	Korwa	Jamatia	-0.0475	-33.135
Cambodian	Mbuti Pygmies	Birhor	Tripuri	-0.0494	-33.95
Cambodian	Mbuti Pygmies	Santhal	Tripuri	-0.0498	-37.411
Cambodian	Mbuti Pygmies	Birhor	Jamatia	-0.05	-32.097
Cambodian	Mbuti Pygmies	Santhal	Jamatia	-0.0504	-34.608
Cambodian	Mbuti Pygmies	Gond	Tripuri	-0.0624	-47.927
Cambodian	Mbuti Pygmies	Gond	Jamatia	-0.063	-44.375
Dai	Mbuti Pygmies	Ho	M-Brahmin	-0.022	-18.591
Dai	Mbuti Pygmies	Korwa	M-Brahmin	-0.023	-17.815
Dai	Mbuti Pygmies	Birhor	M-Brahmin	-0.025	-17.306
Dai	Mbuti Pygmies	Santhal	M-Brahmin	-0.0257	-21.135
Dai	Mbuti Pygmies	Ho	Tharu	-0.0294	-23.936
Dai	Mbuti Pygmies	Korwa	Tharu	-0.0304	-23.028
Dai	Mbuti Pygmies	Birhor	Tharu	-0.0323	-21.559
Dai	Mbuti Pygmies	Santhal	Tharu	-0.0331	-26.283
Dai	Mbuti Pygmies	Gond	M-Brahmin	-0.0395	-34.203
Dai	Mbuti Pygmies	Gond	Tharu	-0.0468	-38.66
Dai	Mbuti Pygmies	Ho	Tripuri	-0.0573	-42.197
Dai	Mbuti Pygmies	Ho	Jamatia	-0.0579	-39.512
Dai	Mbuti Pygmies	Korwa	Tripuri	-0.0582	-42.892
Dai	Mbuti Pygmies	Korwa	Jamatia	-0.0589	-39.226
Dai	Mbuti Pygmies	Birhor	Tripuri	-0.0601	-39.221
Dai	Mbuti Pygmies	Birhor	Jamatia	-0.0608	-36.921
Dai	Mbuti Pygmies	Santhal	Tripuri	-0.0608	-43.994
Dai	Mbuti Pygmies	Santhal	Jamatia	-0.0615	-40.52
Dai	Mbuti Pygmies	Gond	Tripuri	-0.0742	-54.544
Dai	Mbuti Pygmies	Gond	Jamatia	-0.0749	-50.065

Supplementary Table 6: Admixture time estimation: Source population 1 and Source population 2 were representatives of respective ancestries. Using two relatively homogenous AAM populations (Jehai and MahMeri) as source for AAM ancestry, East Asian populations from different geographical locations as EA ancestry source, and different admixed population as reference, admixture between the two ancestries was estimated. Using Birhor as source for AAI ancestry, Paniya for ASI and Jamatia for ATB ancestry, admixture dates for these ancestries were estimated.

Source Population 1	Source Population 2	Admixed Population	Estimated time in generations
Mahmeri	Yakut	Mendriq	16.1
Mahmeri	Yakut	CheWong	22
Mahmeri	Yakut	Cambodian	9.5
Mahmeri	Yakut	Tu	7.2
Mahmeri	Yakut	Jamatia	8.4
MahMeri	Daur	Cambodian	8.6
MahMeri	Tu	Cambodian	7.1
MahMeri	Japanese	Cambodian	6.8
MahMeri	Naxi	Cambodian	6.6
Jehai	Yakut	Cambodian	8.8
Jehai	Daur	Cambodian	6.1
Jehai	Tu	Cambodian	8.3
Jehai	Japanese	Cambodian	8.3
Jehai	Naxi	Cambodian	8.5
Paniya	Birhor	Kadar	17.5
Paniya	Birhor	Ho	11.1
Paniya	Korwa	Ho	15.6
Paniya	Korwa	Kadar	13.5
Birhor	Jamatia	Tharu	11.3

Supplementary Table 7: Metadata on the 43 ancient genomes. This includes sample ID, excavation site location and country.

Ancient Genome ID	Site	Country
La368	Pha Fhaen	Laos
Ma911	Gua Cha Cave	Malaysia
Vt833	Mai Da Dieu	Vietnam
I0627	Man Bac	Vietnam
I2947	Man Bac	Vietnam
I10973	Man Bac	Vietnam
I0626	Man Bac	Vietnam
I1859	Man Bac	Vietnam
I1137	Man Bac	Vietnam
I1135	Man Bac	Vietnam
I2731	Man Bac	Vietnam
Vt880	Hon Hai Co Tien	Vietnam
I8978	Ban Chiang	Thailand
I4011	Oakaie	Myanmar
I7238	Oakaie	Myanmar
I8974	Ban Chiang	Thailand
I8977	Ban Chiang	Thailand
I4458	Ban Chiang	Thailand
La364	Tam Pa Ping	Laos
Vt778	Nam Tun	Vietnam
I8970	Ban Chiang	Thailand
Ma912	Gua Cha Cave	Malaysia
La727	Tam Hang	Laos
Vt777	Mai Da Dieu	Vietnam
Vt781	Nui Nap	Vietnam
Vt808	Nui Nap	Vietnam
Vt779	Nui Nap	Vietnam
In662	Loyang Ujung Cave	Indonesia
Vt796	Nui Nap	Vietnam
I2497	Nui Nap	Vietnam
I2948	Nui Nap	Vietnam
I1680	Vat Komnou	Cambodia
In661	Loyang Ujung Cave	Indonesia

Phl534	Nagsabaran	Philippines
Th519	Long Long Rak	Thailand
Th521	Long Long Rak	Thailand
Th703	Long Long Rak	Thailand
Th530	Long Long Rak	Thailand
Th531	Long Long Rak	Thailand
Ma554	Supu Hujung4	Malaysia
Ma555	Kinabatagan	Malaysia
Vt719	Hon Hai Co Tien	Vietnam
La898	Tam Hang	Laos

Supplementary Table 8: D statistics with Ancient genomes, AAM, Southern-East Asians and an African population. Here tree topology is of the form *D(((Y,Z)W)X)* where X is Mbuti Pygmies (an African population from HGDP dataset), W is Cambodian (Southern East Asian populations from HGDP), Z belongs to AAM subgroups (Bateq, Kintaq,Jehai, Mendriq, CheWong, MahMeri) and Y belongs to Ancient genomes grouped into 5 time periods.

**W**	**X**	**Y**	**Z**	**D**	**Z score**
Cambodian	Mbuti	Anc_8K	MahMeri	-0.0854	-24.755
Cambodian	Mbuti	Anc_8K	CheWong	-0.0794	-22.142
Cambodian	Mbuti	Anc_8K	Mendriq	-0.0667	-19.172
Cambodian	Mbuti	Anc_8K	Jehai	-0.0621	-17.821
Cambodian	Mbuti	Anc_8K	Kintaq	-0.0588	-16.835
Cambodian	Mbuti	Anc_8K	Bateq	-0.0528	-14.446
Cambodian	Mbuti	Anc_4K	MahMeri	0.0157	3.162
Cambodian	Mbuti	Anc_4K	CheWong	0.0209	4.145
Cambodian	Mbuti	Anc_4K	Mendriq	0.0326	6.597
Cambodian	Mbuti	Anc_4K	Jehai	0.0385	7.86
Cambodian	Mbuti	Anc_4K	Kintaq	0.0426	8.747
Cambodian	Mbuti	Anc_4K	Bateq	0.0494	9.788
Cambodian	Mbuti	Anc_3K	MahMeri	0.0111	3.7
Cambodian	Mbuti	Anc_3K	CheWong	0.0189	6.137
Cambodian	Mbuti	Anc_3K	Mendriq	0.0314	10.454
Cambodian	Mbuti	Anc_3K	Jehai	0.0365	11.842
Cambodian	Mbuti	Anc_3K	Kintaq	0.0404	13.372
Cambodian	Mbuti	Anc_3K	Bateq	0.0461	14.852
Cambodian	Mbuti	Anc_2K	MahMeri	0.0001	0.024
Cambodian	Mbuti	Anc_2K	CheWong	0.0067	1.048
Cambodian	Mbuti	Anc_2K	Mendriq	0.0185	2.964
Cambodian	Mbuti	Anc_2K	Jehai	0.024	3.793
Cambodian	Mbuti	Anc_2K	Kintaq	0.0279	4.52
Cambodian	Mbuti	Anc_2K	Bateq	0.0329	5.221
Cambodian	Mbuti	Anc_R	MahMeri	0.0086	2.939
Cambodian	Mbuti	Anc_R	CheWong	0.0172	5.585
Cambodian	Mbuti	Anc_R	Mendriq	0.0298	10.38
Cambodian	Mbuti	Anc_R	Jehai	0.0337	11.482

Cambodian	Mbuti	Anc_R	Kintaq	0.0385	12.962
Cambodian	Mbuti	Anc_R	Bateq	0.0429	14.121
